# Ecological Adaption Analysis of the Cotton Aphid (*Aphis gossypii*) in Different Phenotypes by Transcriptome Comparison

**DOI:** 10.1371/journal.pone.0083180

**Published:** 2013-12-23

**Authors:** Zhao-Qun Li, Shuai Zhang, Jun-Yu Luo, Chun-Yi Wang, Li-Min Lv, Shuang-Lin Dong, Jin-Jie Cui

**Affiliations:** 1 State Key Laboratory of Cotton Biology, Institute of Cotton Research of CAAS, Anyang, China; 2 Education Ministry Key Laboratory of Integrated Management of Crop Diseases and Pests, College of Plant Protection, Nanjing Agricultural University, Nanjing, China; Queen's University Belfast, United Kingdom

## Abstract

**Background:**

The cotton aphid, *Aphis gossypii* Glover, is a destructive insect pest worldwide; it directly or indirectly damages (virus transmission) 300 species of host plants. Knowledge of their ecologically adaptive mechanisms at the molecular level may provide an essential and urgent method to effectively control this pest. However, no transcriptome information is available for the cotton aphid and sequence data are scarce. Therefore, we obtained transcriptome data.

**Results:**

To facilitate such a study, two cotton aphid transcriptomes at different growth stages of cotton, seedling and summer, were sequenced. A total of 161,396 and 66,668 contigs were obtained and assembled into 83,671 and 42,438 transcripts, respectively. After combining the raw date for both transcriptomes, the sequences were reassembled into 66,695 transcripts, and 52,160 were annotated based on BLASTX analyses. Comparison of the transcriptomes revealed that summer presented less challenges for the cotton aphids than the seedling stage of cotton. In total, 58 putative heat shock protein genes and 66 candidate cytochrome p450 genes were identified with BLASTX.

**Conclusions:**

Our results form a basis for exploring the molecular mechanisms of ecological adaption in the cotton aphid. Our study also provides a baseline for the exploration of abiotic stress responses. In addition, it provides large-scale sequence information for further studies on this species.

## Introduction

Aside from direct damage, *Aphis gossypii* Glover is an important vector of many viral diseases in the early cotton season [1]. Moreover, high aphid densities later in the cotton season also present risks because they produce copious amounts of honeydew, which blackens the leaf, decreases photosynthetic activity, and contaminates the lint resulting in severely reduced lint quality [2]. In general, there are two cotton aphid breakouts each year, during the seedling of cotton andsummer, and exhibit phenotypic differences due to environmental heterogeneity in China. Morph I, with a larger body size and darker color (usually dark green or black) is found on seedlings and young cotton plants, where they reproduce parthenogenetically and cause direct feeding damage. Morph II are again asexual but are smaller and light green in colour and are found on older plants during the summer where they resist high temperatures and have a high fecundity resulting in high levels of feeding damage [3].

The evolutionary direction of environmental responses varies in phytophagous insects. Some species evolve towards dormancy [4] and diapause [5], and others towards migration [6]. Heat shock proteins (Hsps) play a vital role in dealing with environmental stress [7]. These proteins function as molecular chaperones that maintain or return proteins to their functional state, prevent the indiscriminate aggregation of denatured proteins, and target unfolded or aggregated proteins for degradation or removal [8]. Another important protein is the cytochrome P450 enzyme (P450), which metabolizes a wide range of endogenous and exogenous deleterious substances to protect live cells [9], [10]. P450 is involved in essential physiological functions in insects, *e.g.*, signal molecule metabolism, adaptation to host plants, xenobiotic metabolism, insecticide resistance, and odorant processing in the antennae [11]–[13]. Several P450s are inducible by phenobarbital, pesticides, and natural compounds [14]. Therefore, we are interested in studying the molecular pathways involved in cotton aphid thermotolerance and elucidating the phenotypic plasticity that allows them to occupy a wide variety of environments. Knowledge of insect ecological adaptability is also of practical importance in potential applications for pest control.

Cotton aphid control has relied largely on the use of chemical insecticides resulting in the development of high levels of resistance [15]. Aphids can very quickly become a major problem when chemical controls fail because of resistance; developing a method to effectively control this pest is essential and urgent. Deep sequencing data can provide extensive information on genomes and gene expression profiles. Such data could serve as a valuable resource ultimately leading to more effective pest control methods. Transcriptome sequencing has been successfully used in many insects [16]–[23], but no such information has been reported for cotton aphids. Therefore, studies on the ecological adaptation mechanisms at the molecular level using comparative transcriptome analysis in this species will be of great value to researchers and growers.

In this study, we constructed two whole-body cDNA libraries, from Morph I and Morph II, and obtained 83,671 and 42,438 distinct transcripts by Illumina RNA sequencing and sequence assembly, respectively. In total, 58 putative Hsps and 66 candidate P450s were identified with BLASTX. In addition, 40 Hsp gene expression profiles were compared among different morphological and developmental stages. Disruption of Hsp and P450 gene products could potentially be exploited in a novel control strategy.

## Results and Discussion

### Transcriptome sequencing and sequence assembly

Two libraries from MorphI and MorphII were constructed. A total of 26,107,304, and 29,230,678 raw reads were obtained from the two transcriptomes (Table 1), respectively. After cleaning and quality checks, the clean reads were assembled into 161,396 and 66,668 contigs with a N_50_ length of 1180 and 446 nt. The size distributions of the contigs are shown in Figure S1. The contigs were further assembly by connecting the contigs based on the paired-end reads for gap filling between each two contigs to build transcripts sequence with the least Ns. Finally 83,671 and 42,438 transcripts were obtained, with mean lengths of 953 and 561, respectively (Table 1). The length distributions of the transcripts are given in Figure S2A. After combining the transcriptomes, 66,695 transcripts were abtained, with a mean length of 860 and N_50_ 1492 (Table 1). Of these assembled transcripts, 31,704 (47.54%) were >500 bp in length. The size distributions of the combined transcripts are given in Figure S2B. The clean reads obtained in this study have been submitted and available from the NCBI/SRA database (SRA experiment accession number: SRX286372).

**Table 1:** Summary statistics of the *Aphis gossypii* transcriptomes.

	Morph I	MorphII	Combined
Total raw reads	26,107,304	29,230,678	55,337,982
Total clean reads	21,602,996	23,947,206	45,550,202
Total clean nucleotides (nt)	2,160,299,600	2,394,720,600	4,555,020,200
Q_20_ percentage	96.74%	96.94%	—
N percentage	0.00%	0.00%	—
GC percentage	33.51%	34.80%	—
Contigs	161,393	66,668	—
Total length of contigs	46,081,473	28,405,764	—
Mean length of contigs	286	426	—
N50 of contigs	446	1180	—
Transcripts	83,671	42,438	66,695
Total length of transcripts	46,931,901	40,429,861	57,364,802
Mean length of transcripts	561	953	860
N50 of transcripts	982	1914	1492
Total consensus sequences	83,671	42,438	66,695
Distinct clusters	21,280	11,981	21,272
Distinct singletons	62,391	30,457	45,423

### Gene identification and annotation

**Figure 1:**
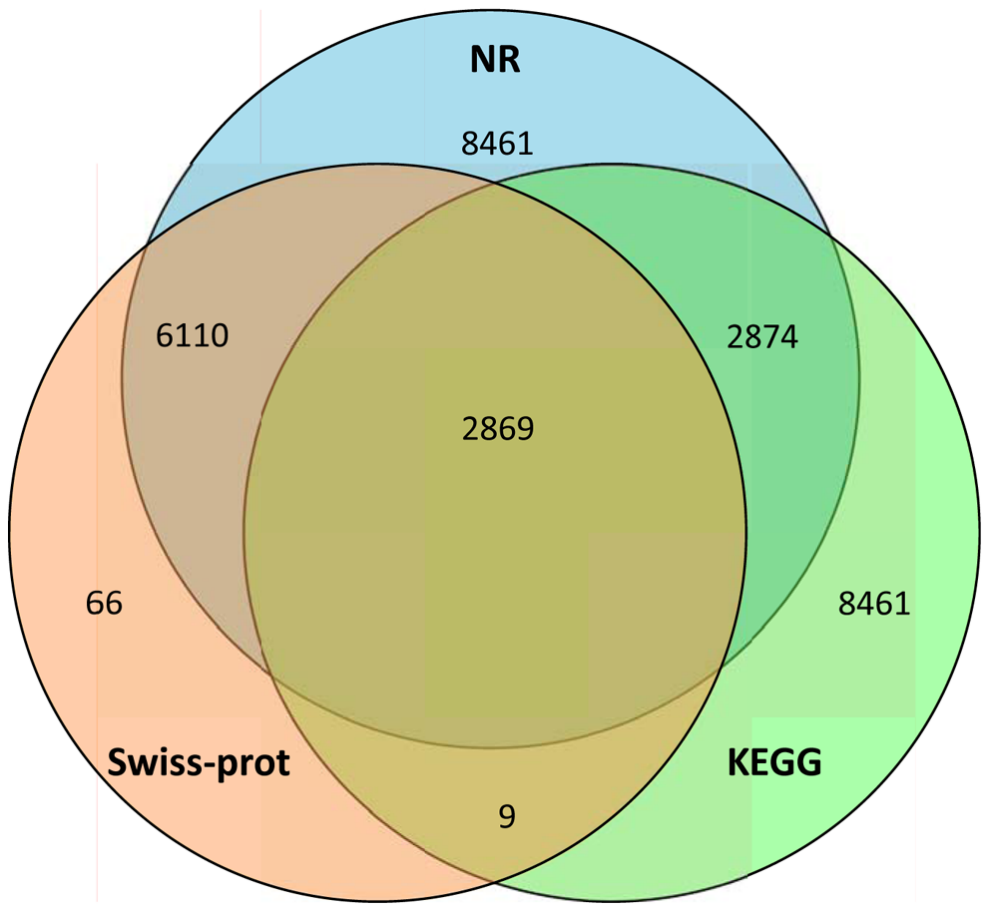
Distribution of similarity search results illustrated by Venn diagrams. The numbers are the sum of transcripts annotated by NR, Swiss-prot, and KEGG. The overlap regions among the three circles contain the number of transcripts that share BLASTX similarity with respective databases.

**Figure 2:**
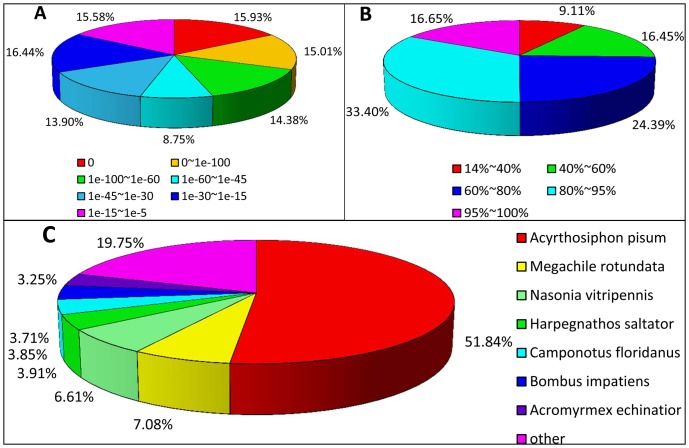
Characteristics of homology search of sequences against the Nr database. (A) E-value distribution of BLAST hits for each distinct sequence with a cut-off E-value of 10^−5^. (B) Similarity distribution of BLAST hits for each unique sequence. (C) Transcripts were searched against the NR protein database using BLASTX with a cutoff E-value <10^−5^ and the proportions of each species (represented by different colors) graphed. Species with proportions of >3% are shown.

To annotate these transcripts, all distinct sequences longer than 200 bp were searched against the NR, Swiss-prot, and KEGG protein databases by BLASTX with a cut-off E-value of 10^−5^ (Table S1). According to this method, a total of 46,137 (69.18% of all distinct sequences), 34,877 (52.29%), and 31,677 (47.50%) transcripts were annotated by NR, Swiss-prot, and KEGG, respectively (Figure 1). A much higher cut-off E-value of 10^−20^ was also used to search against NR, Swiss-prot, and KEGG, and 36,951, 24,187, and 23,100 transcripts were returned in the BLASTX results, respectively. In total, 58 putative Hsps and 66 candidate P450s were identified with BLASTX (Table S2 and File S1). The E-value distribution of the transcripts annotated by the NR database are shown in Figure 2A, and the similarity distribution varied from 14% to 100% (Figure 2B). Overall, about 68% of the annotated transcripts had an E-value <10^−30^ and 18,494 sequences had a similarity >80%.

The species distribution of the transcripts was annotated with the NR database. The cotton aphid sequences exhibited substantial matches with *Acyrthosiphon pisum* (51.84%) followed by *Megachile rotundata* (7.08%), *Nasonia vitripennis* (6.61%), *Harpegnathos saltator* (3.91%), *Camponotus floridanus* (3.85%), *Bombus impatiens* (3.71%), and *Acromyrmex echinatior* (3.25%) (Figure 2C). These percentages mean that the cotton aphid transcripts identified are most similar to *A. pisum* and reveal a high degree of sequence homology between hemiptera and hymenoptera.

In addition, all of the 58 putative Hsp genes were used to confirm the transcriptome assemblies by sequencing their polymerase chain reaction (PCR) products. The sequences had ≥99% identities at the nucleic acid level with corresponding sequences from the combined transcriptome, indicating that the algorithm has worked as expected.

### Phylogenetic analysis

#### Hsp genes

**Figure 3:**
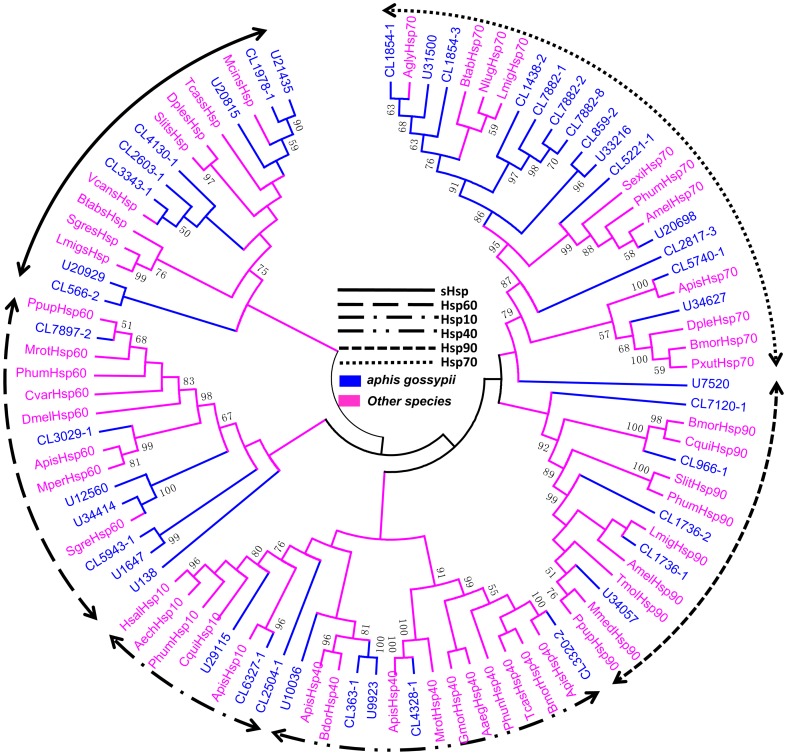
Phylogenetic tree of candidate Hsps from cotton aphids and other species. The cotton aphid transcripts are labeled CL (clusters) and U (transcripts). CL-X: CL-contigX. Values at the nodes indicate bootstrap percentages based on 1000 replicates and branches with bootstrap values above 50% are indicated.

Of the Hsps identified, 43, along with 50 sequences from other insects, were used to construct a phylogenetic tree. Of the 43 transcripts used in the phylogenetic analysis, 39 contained complete open reading frames (ORFs) with putative start and stop codons and four corresponded to partial sequences based on sequence analysis. According to the phylogenetic analysis, the cotton aphid Hsps segregated into six families: Hsp90, Hsp70, Hsp60, Hsp40, sHsp, and Hsp10 (Figure 3).

Of the 43 Hsps, seven candidate Hsp60 and three Hsp10 genes were identified. Hsp10 is analogous to the bacterial GroES subunit that co-chaperones Hsp60 for protein folding (Figure 3) [24], [25]. In addition, Hsp10 plays an important role in protecting cells from stress caused by infection and inflammation [26]. The diverse physiological functions are associated with its changeable location in cellular compartments [27]–[30]. Hsp60s are the most universal molecular chaperones being present in all kingdoms, *e.g.*, eubacteria, eukaryotic organelles (group I), archaea, and the eukaryotic cytosol (group II). They all share a common structure and basic functional mechanism preventing the indiscriminant aggregation of unfolding proteins by oligomerizing in two rings placed back-to-back [31], [32]. Most research on Hsp10 and HSP60 has focused on humans [33]. Their roles in insects have not yet been clearly defined. The cotton aphid transcriptome analysis gives an overview of Hsp10 compared with other insects and provides sequence and phylogenetic information for future studies.

The sHsps are small molecular mass heat shock proteins (12–43 kDa) that function as molecular chaperones by forming a folding-competent state that helps the cell to withstand stresses from the indiscriminate aggregation of denatured proteins in an ATP-independent manner [7], [34]. Recently, some sHsps have been identified from *Bombyx mori* (16), *Drosophila melanogaster* (11), *Apis mellifera* (10), *Tribolium castaneum* (10), and *Anopheles gambiae* (7) by genome sequencing [35]–[37]. In our study, eight sHsp homologous sequences were identified (Figure 3). Six of these transcripts have complete ORFs. To characterize these genes, functional and structural studies are needed to provide insights on the detailed mechanisms of their function and role in development.

Hsp40/DnaJ, which are important for protein translation, folding, unfolding, translocation, and degradation, are Hsp70-mediated ATPase activity co-factor/co-chaperones [7]. Hsp/DnaJ can bind to Hsp70 and be categorized into three groups by the J domain [38], [39]. In our study, five putative Hsp40 genes were identified, and four of them had complete cDNA lengths (Figure 3).

The Hsp70 family is the most structurally and functionally conserved group [40]. Under normal physiological conditions, Hsp70s function in the routine *de novo* folding of proteins. Yet, under stress they prevent the aggregation of indiscriminate proteins by tightly binding unfolding proteins and can even refold aggregated proteins [41]. Some studies have revealed that Hsp70 may contribute to mosquito dehydration tolerance [42] and respond to ultraviolet A (UVA) exposure [43]. Eleven putative Hsp70 genes were identified (Figure 3). The function of these candidate genes requires further study.

Hsp90s are special proteins present at very high concentrations under normal physiological conditions and are up-regulated by stress [7], [44]. Under heat shock, they act as a sequestration unit with other co-chaperones and play a more basic holding role preventing the aggregation of unfolded proteins [7], [45]. We identified six putative Hsp90 genes from the cotton aphid transcriptome (Figure 3). The function of Hsp90 under normal and stress conditions in cotton aphids requires further study.

#### P450 genes

**Figure 4:**
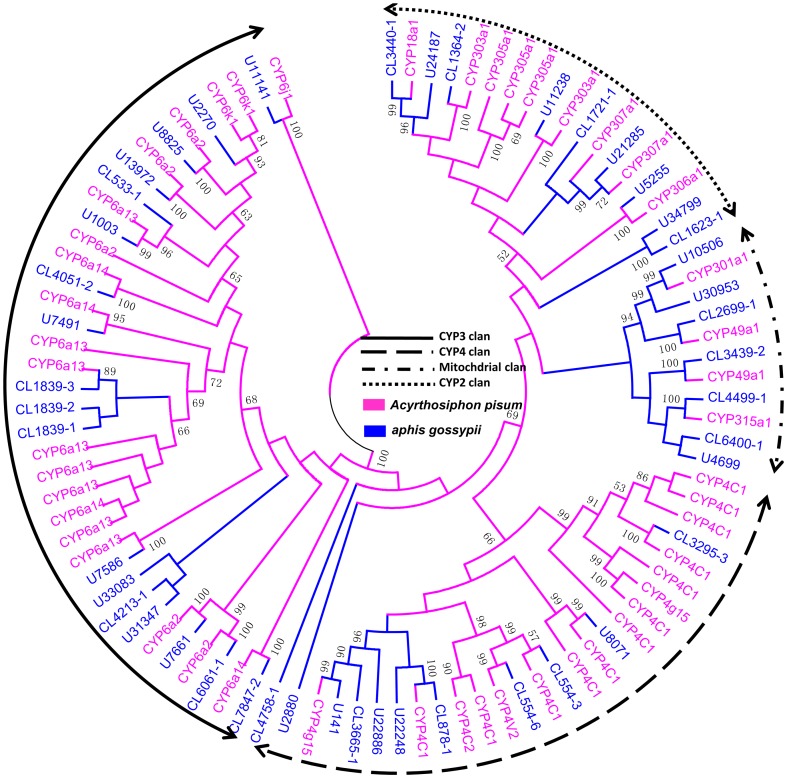
Phylogenetic tree of candidate cytochrome P450s from cotton aphids and *A. pisum*. The cotton aphid transcripts are labeled CL (clusters) and U (transcripts). Apis: *A. pisum*. CL-X: CL-contigX. Values at the nodes indicate bootstrap percentages based on 1000 replicates and branches with bootstrap values above 50% are indicated.

Of the 66 putative P450 genes, 45 genes (containing 23 full-length sequences) >230 amino acids in length were used to construct a phylogenetic tree along with 49 *A. pisum* P450s (Figure 4). The phylogenetic analysis results revealed that these 45 predicted P450s belonged to four major clades [14], namely the CYP2, CYP3, CYP4, and mitochondrial clades. CYP3 were the most numerous of the P450 genes.

Nine of the putative P450 genes belong to the CYP2 family; these may be involved in essential physiological functions. Eighteen candidate P450 genes were identified as CYP3 members, which are involved in xenobiotic metabolism and insecticide resistance, several are inducible by phenobarbital, pesticides, and natural products [14]. Eleven sequences were putative members of the CYP4 family, some of these clade members are clearly inducible by xenobiotics and are linked to odorant or pheromone metabolism [14]. Seven putative mitochondrial P450 members were also identified in our study (Figure 4).

### Transcriptome changes in different life stages

In this study, comparative analysis of two transcriptomes in different life stages was used to determine gene expression. Although more raw reads were sequenced from the MorpII than the MorpI transcriptome, there were 42,438 transcripts assembled in MorpII which was far fewer than in MorpI (83,671 about 50%) (Table 1). This observation suggests that, to develop an energy-efficient mode of life, genes unnecessary for cotton aphid survival might be silenced or expressed at low levels. In contrast, genes involved in reproduction and metabolism are likely expressed at higher levels.

**Figure 5:**
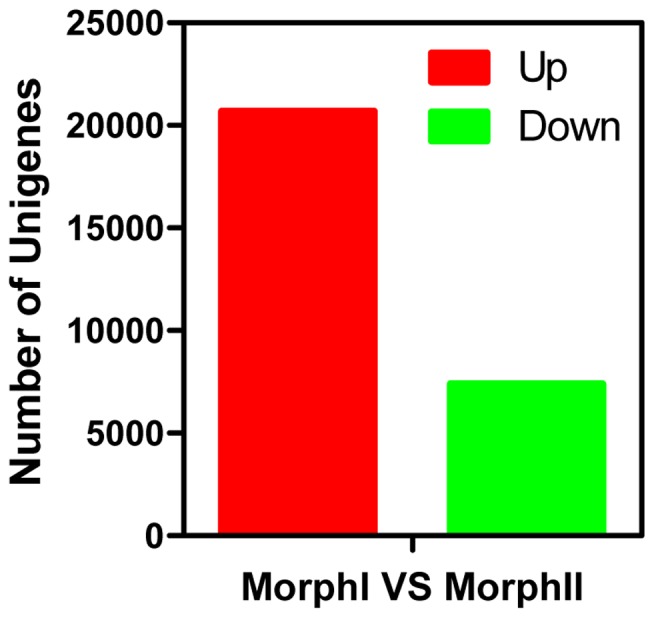
Differentially expressed transcripts in different cotton aphid life stages. Numbers of up- and down-regulated transcripts compared between the MorphI and MorohII.

To evaluate gene expression abundances, the absolute value of the log2 ratio of FPKM (Fragments Per Kilobase per Million mapped reads) ≥1 and false discovery rate (FDR) were used as the threshold to judge the significance of gene expression differences. In the comparative analysis, 20,679 transcripts were noticeably up-regulated in MorpI and 7393 MorpII transcripts were expressed at significantly higher levels (Figure 5). Thus, more genes were necessary for cotton aphids to cope with environmental impacts during the seedling stage of cotton than summer. In other words, environmental variables are more harmful to aphids in the seedling stage of cotton than to those in the summer. The ambient environment experienced during the summer might be less stressful for cotton aphids.

To further compare the two transcriptomes, we give insights into 100 of the most differentially up-regulated MorpI and MorpII transcripts (Table S3 and S4). Of the top 100 up-regulated MorpI transcripts, 42 ribosomal proteins, 5 metabolic enzymes, 4 Hsps, and 4 cytoplasmic oxidases were annotated. However, no ribosomal proteins, Hsps, or cytoplasmic oxidases were identified from the top 100 up-regulated MorpII transcripts. In contrast, 15 metabolic enzymes, including maltase, glucose dehydrogenase, and aspartate aminotransferase, were annotated in the top 100 up-regulated MorpII transcripts. The enrichment of cytoplasmic oxidases may inhibit metabolic enzymes. This could explain why many metabolic enzymes were down-regulated in seeding stage, as in the aphid *Macrosiphum euphorbiae* [46]. The up-regulated of Hsps and cytoplasmic oxidases, which deal with hostile environmental factors, may indicate an uncomfortable eco-environment during this period. However, the up-regulation of metabolic enzymes and the absence of ribosomal proteins, Hsps, and cytoplasmic oxidases in the top 100 revealed that metabolism increases and many genes are either translated into proteins at low levels or not at all. Compared with MorpI genes, the up- and down-regulation of MorpII genes may indicate an energy-efficient mode of life has been developed in adaptation to the comfortable summer environment. This is consistent with our conjecture that summer is more comfortable for cotton aphids than the seedling stage of cotton.

### Quantitative real-time PCR validation of Hsps

**Figure 6:**
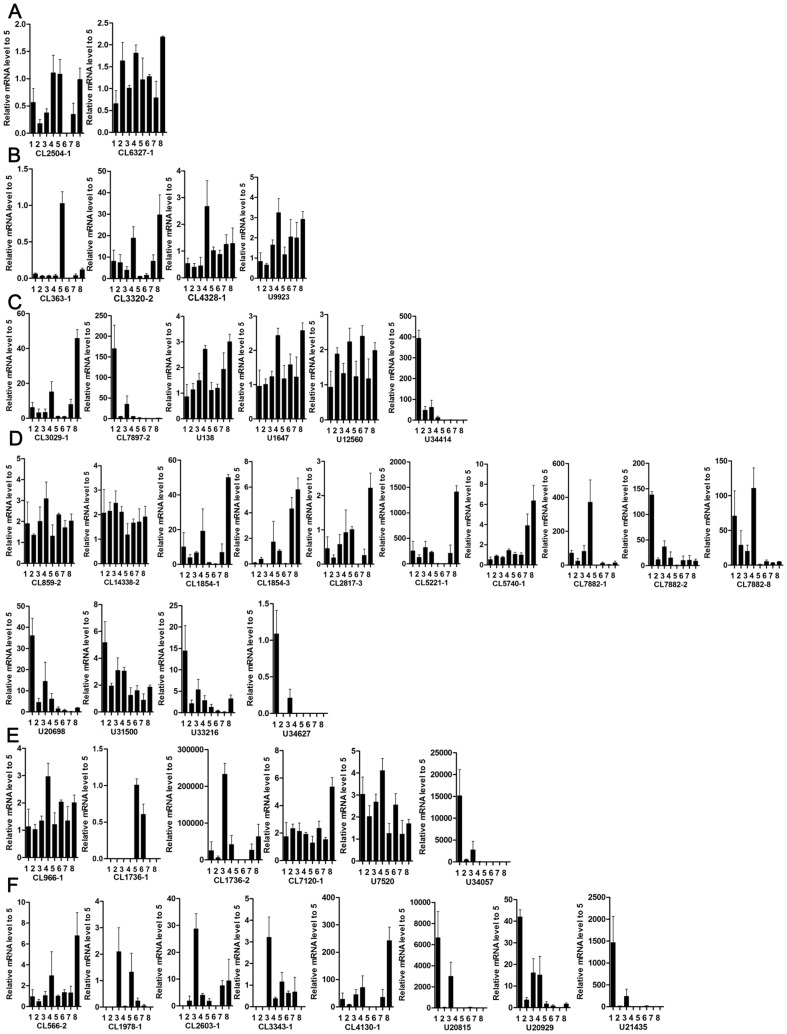
Verification of 48 Hsp genes in different morphological and developmental stages. (A) Hsp10 family gene expression analysis. (B) Hsp40 family gene expression analysis. (C) Hsp60 family gene expression analysis. (D) Hsp70 family gene expression analysis. (E) Hsp90 family gene expression analysis. (F) sHsp family gene expression analysis. 1: unwinged adults of MorphI; 2: unwinged nymphs of MorphI; 3: winged adults of MorphI; 4: winged nymphs of MorphI; 5: unwinged adults of MorphII; 6: unwinged nymphs MorphII; 7: winged adults of MorphII; 8: winged nymphs of MorphII.

To elucidate the function of the Hsp genes identified, we focused on their expressions in different developmental stages. Overall, 14 Hsp genes exhibited obvious MorphI-biased expression; one was specifically expressed in the MorphI. However, 4 Hsp genes were expressed more in the MorphII, and one was MorphII specific. All of the Hsp genes were in fact genes and not transcripts or alternative splicing isoforms, any redundant sequences were removed following BLASTX against the NCBI Nr database and alignment with ClustalX 1.83. The other genes were expressed at similar levels in both stages (Figure 6 and Table S5). These results reveal that cotton aphids require more Hsps to resist the hostile environmental factors experienced during the seedling stage of cotton, which is in accordance with our hypothesis. The specifically expressed Hsp genes may play important roles in dealing with the special hostile environmental factors of that stage. In addition, different Hsp gene expression profiles suggest that they are assigned distinct biological tasks.

### Conclusions

Using next-generation sequencing technology, we have sequenced two transcriptomes and provided large-scale sequence information for the cotton aphid and identified 58 putative Hsps and 66 candidate P450s with BLASTX. Understanding how structure, function, and gene expression interact in the abiotic stress responses of this species will improve biological control in sustainable agriculture. Comparison of the transcriptomes at different life stages revealed that summer was more favorable for cotton aphids than the seedling stage of cotton. Further investigation is required to elucidate the ecological adaptation mechanism.

## Materials and Methods

### Insect samples

Cotton aphids were collected on June 3 (MorphI) and August 5 (MorphII) from cotton field of the Institute of Cotton Research of CAAS. Those that had not been parasitized by parasitic wasps were selected and reared for 2 days in incubators using cotton plants. After 2 days, any parasitized aphids were identified and excluded from the experiment. MorphI were reared in 22±1°C, 65±5% relative humidity (RH), and a 14-h light: 10-h dark photoperiod. MorphII were reared in 28±1°C, 65±5% RH, and a 14-h light: 10-h dark photoperiod. The rearing temperatures were set according to the environmental conditions under which the aphids were collected. One hundred each of MorphI and MorphII were used for transcriptome sequencing. One hundred unwinged nymphs, unwinged adults, winged nymphs, and winged MorphI and MorphII adults, were collected and stored at −80°C until required in three replicates for expression profiling by real-time-PCR. There are no replicates for the RNA-seq samples.

### cDNA library construction and Illumina sequencing

Total RNA was extracted with the SV Total RNA Isolation System (Promega, Madison, WI, USA) following the manufacturer's protocol. cDNA library construction and Illumina sequencing of the samples were performed at the Beijing Genomics Institute, Shenzhen, China [47]. Poly-adenylated RNAs were isolated from 20 µg of pooled total RNA using oligo (dT) magnetic beads and fragmented into short fragments in the presence of divalent cations in fragmentation buffer at 94°C for 5 min. Using these cleaved, short fragments as templates, random hexamer primers were used to synthesize first-strand cDNA. Second-strand cDNA was generated using buffer, dNTPs, RNAseH, and DNA polymerase I. Following end repair and adaptor ligation, short sequences were amplified by PCR and purified with a QIAquick® PCR extraction kit (Qiagen, Venlo, The Netherlands), and sequenced on a HisSeq™ 2000 platform (San Diego, CA, USA). The reaction conditions of the first- and second-strand cDNA synthesis and end repair and adaptor ligation are given in File S2.

### Assembly and function annotation

Transcriptome *de novo* assembly was carried out with the short read assembly program Trinity (version 20120608, the assembly process is shown in File S3) [48], which generated two classes of transcripts: clusters (prefix CL) and singletons (prefix U). Transcripts larger than 150 bp were aligned by BLASTX to the Nr (release-20121005) (ftp://ftp.ncbi.nih.gov/blast/db/), Swiss-Prot (release-2012_08) (ftp://ftp.uniprot.org/pub/databases/uniprot/previous_releases/), KEGG (release 63.0) (http://www.genome.jp/) [49], and COG (release-20090331) (http://www.ncbi.nlm.nih.gov/COG/) (E-value <10^−5^) databases, retrieving proteins with the highest sequence similarity with the given transcripts along with their protein functional annotations based on different databases. We then used the Blast2GO program [50] for GO annotation of the transcripts and WEGO software [51] to plot the GO annotation results. In addition, to confirm transcriptome assemblies, all of the 58 putative Hsp genes were used by sequencing their polymerase chain reaction (PCR) products. Gene specific primers (Table S6) were designed using Primer Premier 5.0 and synthesized by Sangon Biotech Co., Ltd (Shanghai, China).

### Phylogenetic analysis

The 43 Hsp and 45 P450 conceptually translated sequences from the cotton aphid transcriptome annotation from the Nr database, along with sequences from other insect species (Table S7 and S8), were used to construct three phylogenetic trees based on the amino sequences. The Hsp data set contained 58 Hsps from other species, and the P450 data set contained 49 P450s from *A. pisum*. Amino acid sequences for each protein family were aligned using ClustalX 1.83 at default settings [52]. The phylogenetic trees were constructed using the maximum parsimony method implemented in MEGA5 [53] at default settings, and 1000 bootstrap replicates.

### Analysis of transcript expression differences between the two transcriptomes

The transcript expression abundances were calculated by the FPKM (Fragments Per Kilobase per Million mapped reads) method [54], which can eliminate the influence of different gene lengths and sequencing discrepancy on the calculation of expression abundance [54]. The formula is:
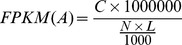



FPKM (A) is the expression of gene A; C the number of reads that uniquely aligned to gene A; N is the total number of fragments that uniquely aligned to all transcripts; and L is the number of bases on gene A.

In our analysis, FDR≤0.001 and the absolute value of the log_2_ ratio ≥1 were used as thresholds to judge the significance of gene expression differences [55].

### Quantitative real time PCR and data analysis

The quantitative real time PCR was performed using the Mastercycler® ep realplex (Eppendorf, Germany). Gene specific primers (Table S9) were designed using Beacon Designer 7.6 and synthesized by Sangon Biotech Co., Ltd (Shanghai, China). Dimethyladenosine transferase [GenBank: KF018923] and peptidyl-prolyl *cis-trans* isomerase [GenBank: KF018924] were used as endogenous controls. The reaction was performed as follows: 2 min at 95°C, followed by 40 cycles at 95°C for 15 s, 55°C for 30 s, and 72°C for 30 s. A melting curve was used to detect a single gene-specific peak and the absence of primer dimer peaks. GoTaq® qPCR Master Mix (Promega, Madison, WI, USA) was used to measure the mRNA levels according to the manufacturer's instructions. A five-fold dilution series was used to construct a relative standard curve to determine the PCR efficiencies and for quantification analysis. Each reaction was run in triplicate (technical repeat) with three independent biological replicates. Relative quantification of Hsp and P450 genes were calculated by the comparative 2^−ΔΔCT^ method [56] to identify the relative mRNA levels of the samples from different life stages.

## Supporting Information

Figure S1 **Length distribution of cotton aphid contigs.** Length distributions of transcripts in the MorphI are highlighted in red and those of the MorphII in blue. All contig sizes were calculated. Transcript lengths (nt) are given on the X-axis and transcripts numbers on the Y-axis.
(TIFF)

Figure S2 **Length distribution of cotton aphid unigenes.** (A) Transcript length distributions in the MorphI are highlighted in red and those in the MorphII in blue. (B) Transcript length distributions in the combined transcriptomes. All contig sizes were calculated. Transcript lengths (nt) are given on the X-axis and transcripts numbers on the Y-axis.
(TIFF)

Table S1 Statistics of annotation results.
(DOCX)

Table S2 The BLASTX results and digital gene expression profiles of candidate cotton aphid Hsps and P450s. Information includes gene ID in this transcriptome, open reading frame length, gene name, accession number, species, E-value, identity to other proteins, FPKM, and FPKM fold-change.
(XLSX)

Table S3 The top 100 up-regulated MorphI transcripts, including gene name, FPKM, FDR, E-value, and Nr annotation result.
(XLSX)

Table S4 The top 100 up-regulated MorphII transcripts, including gene name, FPKM, FDR, E-value, and Nr annotation result.
(XLSX)

Table S5 Relative mRNA level of 40 Hsp genes in different morphs.
(XLSX)

Table S6 Primers and transcripts used in the transcriptome assembly validation, including transcript names and primer sequences.
(XLSX)

Table S7 Hsps used in phylogenetic tree construction.
(DOCX)

Table S8 *A. pisum* cytochrome P450s used in phylogenetic tree construction.
(DOCX)

Table S9 Primers and transcripts used in qRT-PCR validation, including transcript names and primer sequences.
(XLSX)

File S1 **Nucleic acid sequences of putative Hsps and P450s genes.**
(DOCX)

File S2 **The reaction conditions for first- and second-strand cDNA synthesis, end repair, and adaptor ligation.**
(DOCX)

File S3 **Trinity assemble process.**
(DOCX)
