# Supplementary figures and images for: Ecological Adaption Analysis of the Cotton Aphid (Aphis gossypii) in Different Phenotypes by Transcriptome Comparison
Source: PLoS One. 2013 Dec 23;8(12):e83180. doi: 10.1371/journal.pone.0083180 (PMC3871566; doi:10.1371/journal.pone.0083180)

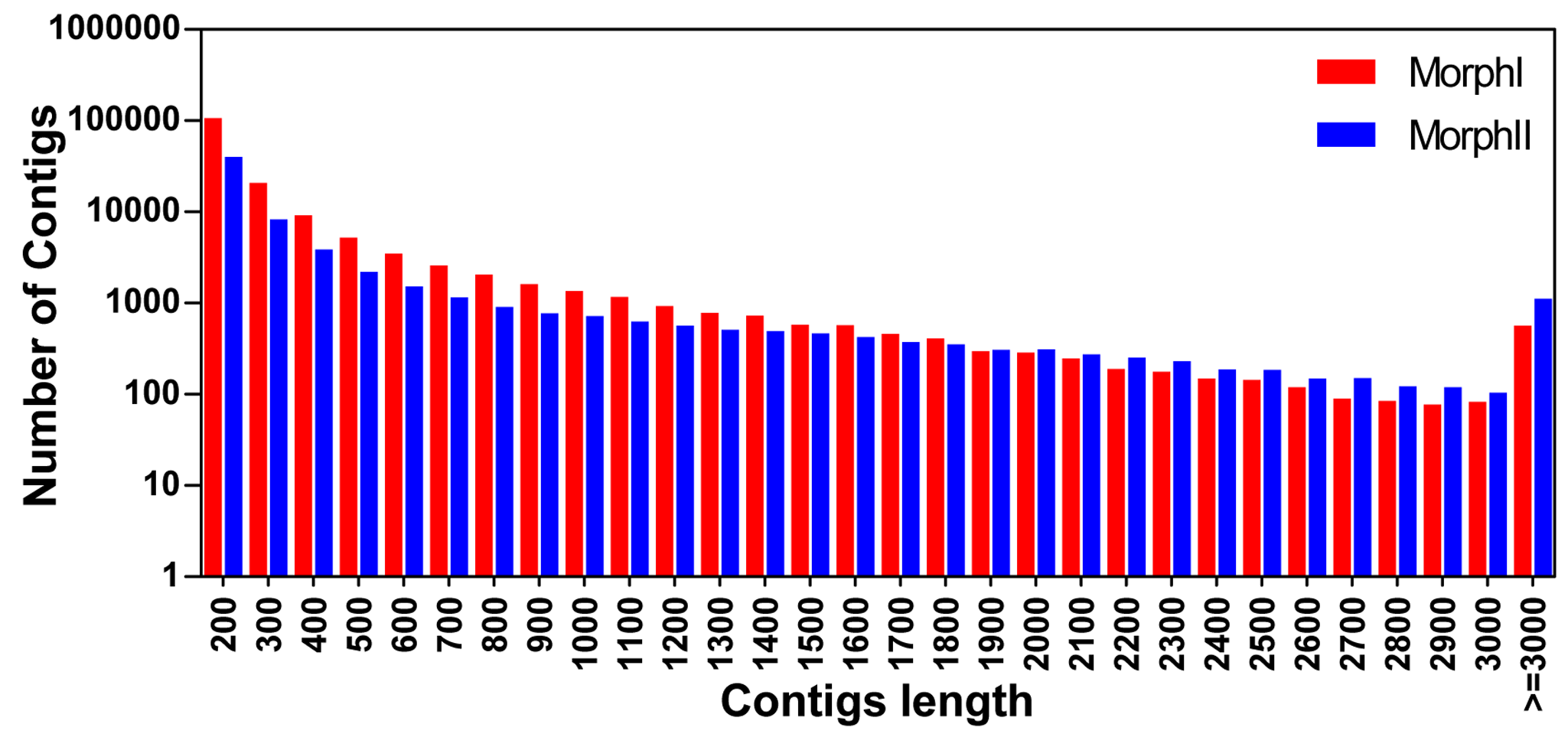

Supplement: Figure S1 — Length distribution of cotton aphid contigs. Length distributions of transcripts in the MorphI are highlighted in red and those of the MorphII in blue. All contig sizes were calculated. Transcript lengths (nt) are given on the X-axis and transcripts numbers on the Y-axis. (TIFF) [file pone.0083180.s001.tiff]

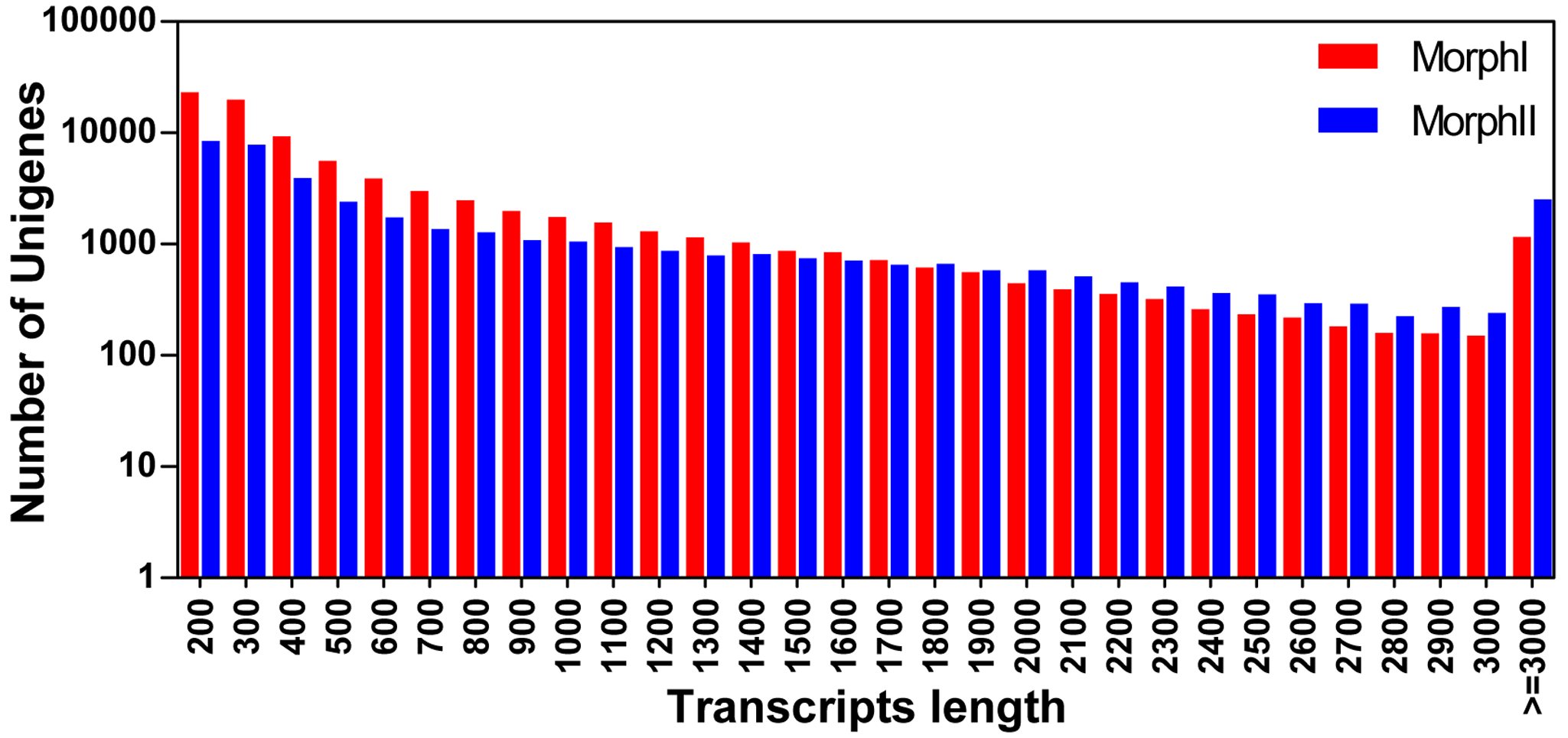

Supplement: Figure S2 — Length distribution of cotton aphid unigenes. (A) Transcript length distributions in the MorphI are highlighted in red and those in the MorphII in blue. (B) Transcript length distributions in the combined transcriptomes. All contig sizes were calculated. Transcript lengths (nt) are given on the X-axis and transcripts numbers on the Y-axis. (TIFF) [file pone.0083180.s002.tiff]
