# Supplementary material for: Ecological Adaption Analysis of the Cotton Aphid (Aphis gossypii) in Different Phenotypes by Transcriptome Comparison
Source: PLoS One. 2013 Dec 23;8(12):e83180. doi: 10.1371/journal.pone.0083180 (PMC3871566; doi:10.1371/journal.pone.0083180)
Supplement: Table S1 — Statistics of annotation results. (DOCX) [file pone.0083180.s003.docx]

**Table S1.** Statistics of annotation results

|  | NR | Swiss-prot | KEGG | COG | GO | All |
| --- | --- | --- | --- | --- | --- | --- |
| Combined transcripts | 46,137 | 34,877 | 31,677 | 16,253 | 21,674 | 52,160 |
| Percentage (%) | 69.18 | 52.29 | 47.50 | 24.37 | 34.50 | 78.21 |
