# Supplementary material for: Ecological Adaption Analysis of the Cotton Aphid (Aphis gossypii) in Different Phenotypes by Transcriptome Comparison
Source: PLoS One. 2013 Dec 23;8(12):e83180. doi: 10.1371/journal.pone.0083180 (PMC3871566; doi:10.1371/journal.pone.0083180)
Supplement: Table S7 — Hsps used in phylogenetic tree construction. (DOCX) [file pone.0083180.s009.docx]

| Hsp | Specie | Acc No. | Hsp | Specie | Acc No. |
| --- | --- | --- | --- | --- | --- |
| NlugHsp70 | Nilaparvata lugens | ADE34170 | MrotHsp60 | Megachile rotundata | XP_003700538 |
| BtabHsp70 | Bemisia tabaci | AAZ17399 | PpupHsp60 | Pteromalus puparum | ACO57619 |
| LmigHsp70 | Locusta migratoria | AAP57537 | TmolHsp90 | Tenebrio molitor | AFN02497 |
| SexiHsp70 | Spodoptera exigua | ACM78945 | LmigHsp90 | Locusta migratoria | AAS45246 |
| AmelHsp70 | Apis mellifera | NP_001153520 | AmelHsp90 | Apis mellifera | NP_001153536 |
| PhumHsp70 | Pediculus humanus | XP_002428084 | SlitHsp90 | Spodoptera exigua | ADK55517 |
| ApisHsp70 | Acyrthosiphon pisum | XP_001945768 | PhumHsp90 | Pediculus humanus | XP_002428463 |
| PxutHsp70 | Papilio xuthus | BAM19890 | BmorHsp90 | Bombyx mori | AFG30049 |
| BmorHsp70 | Bombyx mori | AFN02501 | CquiHsp90 | Culex quinquefasciatus | XP_001861262 |
| DpleHsp70 | Danaus plexippus | EHJ78227 | MmedHsp90 | Microplitis mediator | ABV55506 |
| AglyHsp70 | Aphis glycines | AFO70211 | PpupHsp90 | Pteromalus puparum | ACO57617 |
| LmigsHsp | Locusta migratoria | ABC84494 | AechHsp10 | Acromyrmex echinatior | EGI60182 |
| SgresHsp | Schistocerca gregaria | AEV89760 | HsalHsp10 | Harpegnathos saltator | EFN79770 |
| BtabsHsp | Bemisia tabaci | ACH85196 | PhumHsp10 | Pediculus humanus | XP_002428683 |
| TcassHsp | Tribolium castaneum | XP_973442 | CquiHsp10 | Culex quinquefasciatus | XP_001848951 |
| SlitsHsp | Spodoptera litura | ADK55524 | ApisHsp10 | Acyrthosiphon pisum | NP_001119666 |
| DplesHsp | Danaus plexippus | EHJ69639 | AaegHsp40 | Aedes aegypti | ABF18277 |
| VcansHsp | Venturia canescens | AAV48822 | GmorHsp40 | Glossina morsitans | ADD18658 |
| McinsHsp | Macrocentrus cingulum | ACF21815 | PhumHsp40 | Pediculus humanus | XP_002426657 |
| SgreHsp60 | Schistocerca gregaria | AEV89752 | ApisHsp40 | Acyrthosiphon pisum | NP_001119620 |
| MperHsp60 | Myzus persicae | CAB58441 | BmorHsp40 | Bombyx mori | NP_001040292 |
| ApisHsp60 | Acyrthosiphon pisum | XP_001951373 | TcasHsp40 | Tribolium castaneum | XP_971446 |
| PhumHsp60 | Pediculus humanus | XP_002428684 | ApisHsp40 | Acyrthosiphon pisum | XP_001949061 |
| DmelHsp60 | Drosophila melanogaster | NP_511115 | BdorHsp40 | Acyrthosiphon pisum | AEJ88366 |
| CvarHsp60 | Culicoides variipennis | AAB94640 | MrotHsp40 | Megachile rotundata | XP_003707298 |

**Table S7.** Hsps used in phylogenetic tree construction.
