# Supplementary material for: Ecological Adaption Analysis of the Cotton Aphid (Aphis gossypii) in Different Phenotypes by Transcriptome Comparison
Source: PLoS One. 2013 Dec 23;8(12):e83180. doi: 10.1371/journal.pone.0083180 (PMC3871566; doi:10.1371/journal.pone.0083180)
Supplement: Table S8 — A. pisum cytochrome P450s used in phylogenetic tree construction. (DOCX) [file pone.0083180.s010.docx]

**Table S8.** Cytochrome P450s of *A.* *pisum* used in phylogenetic tree construction.

| P450 | Acc No. | P450 | Acc No. | P450 | Acc No. |
| --- | --- | --- | --- | --- | --- |
| CYP301a1 | XP_001948959 | CYP4g15 | XP_001944205 | CYP6k1 | XP_001943150 |
| CYP49a1 | XP_001946744 | CYP6a2 | XP_001947920 | CYP4C1 | XP_001944051 |
| CYP6a14 | XP_001945100 | CYP6a2 | XP_001951983 | CYP305a1 | XP_001949502 |
| CYP4C1 | XP_001948889 | CYP303a1 | XP_001951093 | CYP307a1 | XP_001948715 |
| CYP4g15 | XP_001944431 | CYP4C1 | XP_001951034 | CYP6a2 | XP_001952450 |
| CYP4C1 | XP_003246356 | CYP6a13 | XP_001948581 | CYP6a13 | XP_001948934 |
| CYP315a1 | XP_001944183 | CYP4C1 | XP_001944043 | CYP306a1 | XP_001947874 |
| CYP6a2 | XP_003248187 | CYP18a1 | XP_001947923 | CYP305a1 | XP_001952620 |
| CYP6a2 | XP_001943981 | CYP6a13 | XP_001946428 | CYP4C1 | XP_003245384 |
| CYP305a1 | XP_001950295 | CYP6k1 | XP_001948421 | CYP6a14 | XP_003242904 |
| CYP6a13 | XP_001946384 | CYP305a1 | XP_001945934 | CYP4C1 | XP_001945361 |
| CYP4C1 | XP_001944487 | CYP4C1 | XP_001948906 | CYP49a1 | XP_001950592 |
| CYP307a1 | XP_001945761 | CYP6a13 | XP_001948488 | CYP4C1 | XP_001952439 |
| CYP6a13 | XP_001948443 | CYP6a14 | XP_001943570 | CYP6j1 | XP_003243002 |
| CYP6a13 | XP_001952727 | CYP6a13 | XP_001943630 | CYP6a14 | XP_001944599 |
| CYP4C1 | XP_001950226 | CYP4V2 | XP_003243610 |  |  |
| CYP4C1 | XP_001951829 | CYP4C1 | XP_001943923 |  |  |
