# Supplementary material for: Ecological Adaption Analysis of the Cotton Aphid (Aphis gossypii) in Different Phenotypes by Transcriptome Comparison
Source: PLoS One. 2013 Dec 23;8(12):e83180. doi: 10.1371/journal.pone.0083180 (PMC3871566; doi:10.1371/journal.pone.0083180)
Supplement: File S1 — Nucleic acid sequences of putative Hsps and P450s genes. (DOCX) [file pone.0083180.s012.docx]

**Additional File 1. Nucleic acid sequences of** **putative Hsps and P450s genes.**

>CL1438.Contig2 TTGTTGAACGAACATACTGTCTTGTGCGACTCTCAGTGCTTTTCACTGCTTTACAAGTCTATACGTGTTTATTAATTATTTTTCCAGTGCGCGTTTAAACTCTCAAGTGATTTTAAACCTTCTAATAATTGATTAGAAATTCGGTATACGATTACTGCAGTATACGGTTTTCTACACGTTTTTGATTTTAATAATATTTCGTTTCGTATTGAAGTAGTGTGTGATCAACAACAACTATTGACGAAATGACTGGAAGAACAGCAATCGGCATCGATTTGGGTACCACATATTCGTGTGTGGGCATATGGCAACACGGTAAAGTCGAGGTGATCGCCAACGATCAAGGTAACAGGACCACCCCGAGTTATGTGGCGTTCACCGACACCGAACGGTTGATCGGCGACGCGGCCAAGAACCAGGTGGCGATGAACCCCGTGAATACAGTGTTCGACGCCAAGCGTTTGATCGGACGTCGTTTCGACGACGAAAAGACACAAGCCGACATTAAACACTGGCCGTTCAAAGTGGTGAACGACAGCGGGAAGCCAAAAATCCAAGTGGAATTCAAAGGCGAACGGAAAGTGTTCGCGCCGGAGGAAATCAGTTCGATGGTGCTGACGAAAATGAAGGAGACCGCCGAAGCGTACTTGGGCCGTGAAGTGACGGACGCCGTGATCACGGTGCCGGCGTACTTTAACGACTCGCAGAGACAGGCGACAAAGGACGCGGGCGTCATAGCCGGCCTGAACGTGATGCGAATAATCAACGAACCGACAGCTGCGGCTTTGGCGTACGGTCTGGACAAGAACCTGAAAGGAGAGAGGAACGTGTTGATATTCGATCTGGGCGGCGGAACGTTCGACGTGTCCGTTCTGCAGATCGACGAGGGTTCGATATTCGAAGTGAAGTCGACGGCGGGCGACACGCACTTGGGCGGCGAAGACTTCGACAACCGGCTGGTGGCTCATCTGGCCGACGAGTTCAAGAGGAAATGCAAAAAAAGACATCAGTGGCAACACGAGAGCTCTCCGACGACTGCGGACGGCCTGCGAAAGGGCCAAACGGACGTTGTCGTCGAGCACCGAAGCGTCTCTCGAAATAGACGCCTTGCACGACGGCATTGATTTTTATTCGAAAATCACCAGGGCCCGATTCGAAGAAATGTGCATGGACTTGTTCCGATCCACACTTTCTCCAGTGGAAAAAGCACTGGCTGACGCTAAAATGGACAAGAGCAGTATACACGATGTAGTACTCGTTGGAGGTTCGACAAGAATACCAAAAATACAGAAAATGTTACAGGACTTCTTTTGCGGTAAATCTCTAAACCTGTCCATCAATCCTGATGAGGCCGTAGCGTACGGAGCTGCCGTCCAAGCCGCCATTCTTAGCGGCGATACCAGTTCAGCCATTCAAGACGTACTTTTAGTTGATGTTACTCCCCTGTCACTCGGTATTGAGACAGCAGGTGGCGTAATGACCAAACTTGTGGAACGCAATTCTCGCATACCGTGTAAACAACAACAAACATTTACTACTTATTCCGACAACCAAGCGGCTGTCACGATCCAGGTATTTGAAGGTGAACGAGCAATGACTAAGGACAACAATTTGTTGGGCACATTTAACTTGACAGGAATCCCTCCTGCACCACGTGGTGTCCCAAAAATAGAAGTGACATTCGACTTGGATGCCAATGGTATTCTCAATGTGTCTGCAAAGGAAATCAGCACTGGGAAATCAGAAAAGATAACTATAAAGAACGACAAAGGACGACTGTCCAAAGAAGATATTGACAAAATGTTGAAAGAAGCGGAGATGTATAGACAAGAGGACGAAAAACAGAAAGAACGCGTTACTGCCAGAAACCAATTGGAGAGCTATGCGTTTAGTGTCAAACAAGCAGTTGAAGAAGCTGGCTCAAAACTATCAGAAACGGACAAGAAATCTGTATTAGAAAGTTGTAAAGCGGTGATCTCGTGGTTGGAAACAAATACATTAGCTGATAAAGATGAGTTTGAAGATAAATTGAAAACCTTACAGAAAGAGT

>CL1736.Contig1

TTTTTTTTTTTGCTTTTTATGAGTTGCACCTTTATTTAAATTATCTGTACAATGGATGTTTTTAACAACATACAAGAAGTCTACTCACAATACAGAATTAATAAAGAGCGGATAATTAGGTAACGCTTAACATTAAAAATATTTGATCAGTTTATGTACAAAAATTATTCCATGTATATTTATTATCTGTAAAACTCGTAAACACCTTATACAACTAGAATATTCCAAGCAAACTTATACATGTGTACATTGAATATTAGATGATTATACAAATCTACCAGATTTAAATAAATTGCCATGTTAGTAATAAATTTTGGAACAAATTTGAAATAAAAAGAAAGAAAAAAAAAAAAAAGAATGGACAAATCAGTCTCAAAATAATATATAACAATAAAAAATATAGGAAATGTTCTTTCAAAATAAATTCACTTTGTAATAAATTAATCAACCTCTTCCATGCGAGATGAGTCTTCAGCATCAGCTTCGACGACAGGCTCGGAGGCTTCAACTTCAGCAGATTTTTCTTCAACTACTGGCAAATCTTCATCAATGCCCAAACCCAATTTGATCATTCGGTGGATTCTGGAAGCGTGAACTTGTGGGTCTTCAAGTCCAAAGCCAGATGACAACAAGCTGGTCTCGAACAAAAGCATGACCAAGTCTCTTACAGCCTTGTCGTTGGAATCAGCTTCGGCTTTTTGTCTGAGTGTCTCAATGATTGGGTGGTCAGGGTTGATTTCCAAGTGTTTCTTGGCTGACATGTAACCCATGGTCGATGAGTCTCTTAGAGCTTGTGCCTTCATGATACGTTCCATGTTGGCAGTCCAACCGTACTGAGATGTGACAATGCAACAGGGTGACTCGACAAGTCTGTTACTGATGACAACTTTTTCAACTTTCTTGTCCAAAATGTCCTTAACGACTTTGCACAATTTCTCGAATCTGGCTTGATCTTCTTCGCGCTTCTTCTTTTCCTCGTCAGTTTCGGGCAAGTCCAAACCTTCTTTGGTGACAGATACCAAGTTCTTGCCGTCGTATTCTTTCATTTGTTGGACGACGTATTCATCAATGGGTTCAGTCATGTAGATGACTTCGAAACCACGCTTCTTGACACGTTCAACGAATGACGAGTTGGATACTTGTTCACGGCTTTCACCAGTGATGTAGTAAATGTGTGTCTGGTTTTGCTTCATGCGTGCAACGTATTCTTTCAAGGAGCATGGTTCGTCACCACTGGCTGAGGAGTGGAATCTCAACAAGTCTGAGAGTTTCTTTCTGTTCTGGCTGTCTTCGTGGATTCCAAGTTTCAAGTTCTTGCTGAACTGTTCGTACAATTTCTTGTAGTTGTCCTTGTCTTCAGCTAATTCTTCGAACAATTCCAAACACTTCTTAACCAAGTTCTTCCTGATCACCTTCAAGATCTTGTTTTGTTGGAGCATTTCACGAGAGATGTTCAATGGCAAATCTTCGCTGTCAACAACACCCTTGATGAAGTTCAAGTATTCAGGCATGAGATCTTCACAGTTGTCCATGATGAAGACACGGCGAACGTACAATTTAATGTTGTTCTTCTTCTTCTTGTTTTCAAACATATCATAAGGCGCGCGCTTGGGAATGAACAACAGTGCTCTGAATTCAAGTTGTCCTTCAACGGAAAAGTGTTTGACGGCTAAATGGTCTTCCCAGTCGTTGGTCAAGGACTTGTAGAATTCACCGTATTCATCTTGGCTGATGTCATCAGGGTTGCGTGTCCAGATTGGTTTGGTCTTGTTCAAGACTTCTTCATCCAAGTACTTCTCTTTGATGGTCTTCTTCTTTTTCTTATCTTTTTCTTCGTCTTTCTTGTCTTCATCTTCATCTTCACCGACATCCTCAATCTTGGGTTTCTTGTCTTCTTCAGTTTCACCTTCGCCATCTTCTTTCTTTTCTTCTTCAGCTTCGTCATCGCTGACTTCCTTGGTACGTTCATTTTCAACAATCAATTTGATCGGGTAGCCAATGAACTGAGAGTGTTTCTTGATGATGCTGGTGATCTTTTCTTGTTGGAGGAACTCAGCTTGGTCTTCTTTGATTTGAAGGACAATCTTAGTACCACGGCCCAATGGTTCACCAGGGTCGGAGCGGATGGTGAATGAACCTCCCGCAGCGGATTCCCACAAGTATTGTTCATCGTCATTGTGCTTGGAAACAACTGTGACCTTGTCGGCAACCAAGTAGGCAGAGTAGAAACCCACACCAAATTGACCAATCATAGAAATGTCAGCACCAGCTTGTAAAGCTTCCATGAAAGCTTTGGTTCCAGATTTGGCGATGGTTCCCAAGTTGTTGACTAAGTCAGCTTTGGTCATACCGATACCAGTATCAATAATGGTCAAAGTTTTTTTCTTCGGCATTTGGGATGATCTTAATGTGTAGGTCTTTGCCAGATTCCAATTTGGATGGGTCGGTCAATGACTCATAACGGATTTTGTCCAATGCATCAGAAGAGTTGGATACCAATTCTCGCAAGAAGATTTCTTTGTTGGAGTAGAAAGTGTTGATGATGAGAGACATGAGCTGAGCGATTTCAGCTTGGAAAGCGAAGGTCTCGACATCTTCAGATGCAGTCATGGTAACGTCTTCAGGCATGATTAATTAGTGTGTTGATAATTGATGATCGCAAATGGAATGAGAATTAAACACTGCAAGCAGTATATCCGCAAATAGTGTTGATCACTTGATCGCAAGTAGTATCGCAGGCTTTAGGGCGTGCGTGTTGAAGGTTCGACAACGAGCTCCAAAATGACGACGATCCCACGGCGGCGGCCGCTATTTATAACATCCCCTGGCCACCGGGCCGAAGA

>CL1736.Contig2

GGAAGGTATTGCAATAAAAACTAAGATTTCAAAATAGAAATTCACCATTAATAGAACAATTAATTTTTTTTCATGATCATACAATCTTTATTATTATCATAATTATATCAACAATTACTTATATAATATTATTTATTATAAAAAATAAATTTATTAATATTAAAATTTCTGAAAATCAAATAATCGAATTTATTTGAACTACAACTCCACCAATTATCTTAATTTTTATTGCAATACCTTCCCTTCACTTACTTTATTTAATAGATGAAATTAAATGTCCTATTTTAACAGTTAAGATTTTTGGACATCAATGATTTTGATCTTATGAATATTCAGATTTTTCTAATATTGAATTTGAATCTTATATAATAAATGAATTAAATAAAGAAAATTTTCGTTTAATTGAAGTAGACAATAAAACCATTCTACCATTTAAATTTAATATTCGATTATTAATTTCATCAGATGATGTAATCCATTCATGAACTATCCCAAGACTAGCAATTAAAATTGATGCAATTCCAGGACGAATAAATCAAATTAACCTTTTCATAAACCGACCAGGAATTTATTTTGGTCAATGCTCAGAAATTTGTGGAATTAATCATAGATTTATACCTATTCAAATTGAATCAATTAATTTAAATAAATTTATTTATTGAATTAAAAATTTTTAATTAAAAAAAAAAAAAAAAAAATTAATAAATACATTGAGTAACTATTGATGATTAAATCAGTCAAAATTATTCATAATAATGAAAAAAAAAAAATTAAGTTTAATAGTAAATACTCAACAAGTATAATACCACAGTCATTTTGTTATTATTTTTTTTTTTTTTTCTTCTCAAAAGTCTTTGGATGTTGTAATTTGTTGTTGTTGTTGTTGTTTTGAAATTGAATTTAATCAACTTCTTCCATTCTTGAAGAATCTTCAGTGGCTTCTTCAACAACTGGTGTAGAATCAGATGAATCTTTGACTTCTTCCATTGGTACATCATCATCTTCAATACCAAGACCCAATTTAATCATACGATGAATTCTAGTTGCATGAACTTGTGGATTTTCAAGGGCAAATCCAGATGCCATTAATGATGTTTCATAAATTAATACAACAAGATCTTTGACTGATTTATCATGTTTGTCAACTTCAATTTTGCTTCTAAGATTCTCGATGATTGGATGATCTGGATTAATTTCAAGATGTTTCTTGGCAGCCATGTAACCCATTGTTGATGTGTCCCTGAGTGCTTGGGCCTTCATGATTCTTTCCATGTTTGCTGTCCAACCATACTGTGATGTGACAATACAACATGGTGAATTAACAAGACGATTTGATACAACAACCTTTTCAACTTTGTTGTCCAAAATGTCTTTCATGACTTTACATAACTCTTCAAATTTAGTCTTGTTTTCTTCCATTAGTTTTTTATCTTCATCAGATTCTGGAAGTTCCAAGCCTTCTTTTGTAATTGAGACCAATTGTTTACCATCAAATTCTTTCAATTGTTGAACAACATATTCATCAATTGGCTCAGTCATGTAGACAACTTCAAAACCACGTTTCTTGATTCTTTCAACAAATGCACTGTTAGCAACTTGATCTTTATTTTCACCAGTAATATAATAAATGTGTTTTTGATTTTCCTTCATACGATCAACGTAGTCTTTCAATGAACACATTTCATCACCAGATGCTGATGTATGATATCTCAATAATTCAGATAATTTTTTTCTATTTGTACTATCTTCATGAATACCAAGTTTGAGATTCTTTGAAAATTGTTCATAGAATTTTTTAAAGTTTTCTTTATCTTCTGATATTTCTTCAAATAGCTCCATGCATTTTTTAACAAGATTTTTACGAATAACTTTGAGAATTTTATTTTGTTGTAACATTTCACGACTGATGTTCAATGGCAAGTCTTCACTGTCAACAACACCCTTCATGAAATTGAGATACTCTGGAATAAGATCTTCACAATTGTCCATGATAAAGACACGTCTAACATACAGCTTGATGTTGTTCTTTTTCTTTTTGTTTTCAAACAAATCAAATGGTGAACGACGTGGTAAAAATAGCAGAGCTTTAAATTCAAGTTGACCCTCAACTGAGAAATGTTTAACAGCTAAATGATCTTCCCAATCATTTGTCAAGCTCTTGTAAAATTCACCATACTCATCTTGAGTAATGTCATCTGGATTTCTTGTCCAAAGTGGCTTTGTTTTATTCAACTCTTCATCATCAATGTATTTTTCTTTAATTGTTTTTTTCTTTTTTTTCTTATCTTTATCATCAGCCTCTTCATCTTCACCAACTTCTTCAATTTTTGGTTGTGTATCATCATCTTTATCTTTATCTTTAACTTCTTCTTCTTCATCATCAGTTAATTCTTTTTCTCTTTCTTTTTCAATGTATAATTTAATTGGATAACCAATAAATTGTGAATGTTTTTTAACAATTTCTTTGATCTTTGACTCTTCAAGATATTCAGCTTGATCTTCTTTAATTGTCAAGATCATTTTAGTACCACGACCAAGTGATTCACCAGTATCTTGACGAATTGTAAATGATCCACCAGCAGATGATTCCCAAATGTATTGTTCGTCATCATTATGTTTTGATATGACTGTTACATTATCAGCAACAAGATATGCTGAATAAAAACCAACACCAAATTGACCAATCATTGATATATCAGCACCAGCTTGAAGTGCTTCCATAAATGCCTTGGTGCCAGATTTAGCAATTGTACCCAAGTTGTTGACAAGGTCAGCTTTAGTCATACCAATACCAGTATCAATGATGGTTAGAGTTCTATCAGCCTTGTTTGGAATGATCTTGATGTGAAGATCCTTGCAGCTGTCAAGCTTGGTTGGATCAGTCAATGATTCATAACGAATCTTGTCCAAGGCATCAGATGAGTTTGAAATTAATTCACGAAGAAATATTTCTTTATTGGAGTAAAACGTGTTAATAATTAGACTCATAAGCTGAGCAATTTCAGCTTGAAATGCAAATGTCTCAACATCACCAGTTTCTACGTTTTCAGGCATCTTGATGTAATTCAATTAAAATTGGTAGTTTGATTTATAAACAATTGCAATAAATTATTATCCTTGAGGATATGCAGTTGAATAGAATACTTTGTTTATGCACGATACGTACAGCTCTGTTTCACTTTTG

>CL1854.Contig1

TTTTTTTTTTAAACATTTGATGATTGCTTTTATTATCAATAGAAAGTAAATGCATAAGAAAAAAAAACACAAGTCATCTGTTGGTGTTCATATGATGTAGGAATGTGCTGAATAGCGAATAGTTTAATCAACTTCTTCAATAGTTGGACCAGCTCCTGAGCCAGCGCCTGGAGCGGCACCACCAGCACCAGCTCCTGGGAATCCAGGCATACCACCGGGACCACCTGGCATACCACCACCAGCACCAGCATACAATTTAGTGATAATTGGGTTGCAAATGCCTTCCAATTCTTTTTGTTTGTGTTCATATTCTTCCTTGTCAGCTAGTTGGTTAGCATCCAACCATTTGATAGTATCATTGACTTTATCAAGAATTACAGTTTTATCGGTATCTGGGATCTTGTCCTTGATTTTTTCATCTTCCATGGTGCTCTTCATGTTGAAACAGTAAGATTCCAAACTGTTCTTGGCAGCAATTACATTCTTTTGTTGTTCATCTTCATTCTTGTATTTTTCGGCGTCATTAACCATGCGCTCGATATCTTCCTTGCTCAAACGTCCCTTGTCGTTGGTGATGGTGATCTTGTTTTCTTTGTTGGTAGACTTTTCAATAGCACTGACGTTCAAAATACCATTAGCATCGATGTCGAAGGTGACTTCGATCTGGGGGACTCCACGTGGTGCGGGGGGAATAGCAGTCAATTCGAATTTTCCCAACAAGTTGTTGTCTTTGGTCATGGCACGTTCTCCTTCATAAACTTGGATCAAGACTCCGGGTTGGTTGTCAGAGTAGGTGGTGAAAGTCTGGGTCTGTTTGGTCGGGATGGTTGTGTTACGCTTGATGAGAGCGGTCATCACACCACCAGCAGTTTCAATACCCAAAGACAGAGGAGTGACATCGAGCAACAACAAGTCTTGGACTTCTTCAGATTTGTCTCCGTGCAAGATGGCAGCTTGTACTGCGGCACCATAGGCAACAGCTTCATCGGGATTGATGGATTTGTTCAATTCCTTGCCGTTGAAGAAGTCTTGTAACAATTTCTGTACTTTGGGGATACGGGTAGAACCTCCAACCAATACAATGTCATTGATGGCAGACTTGTCCATCTTAGCGTCACGTAGAGATTTTTCTACGGGTTCCATGGTACTACGGAAAAGATCGGCGTTCAATTCTTCAAAACGAGCACGAGTGATGGATGTGTAGAAGTCGACACCTTCAAACAACGAATCAATTTCAATGCTTGCTTGGGTGGAAGAGGAAAGAGTACGCTTTGCACGTTCGCAGGCAGTCCTCAAACGTCTCAAGGCTCTTTTGTTGGTGGTCACGTCCTTCTTGTACTTGCGTTTGAACTCTTGTACAAAGTGGTTGACCATTCTGTTGTCAAAATCTTCACCTCCCAAGTGAGTGTCTCCAGCAGTGGACTTGACTTCGAATATTCCATCTTCGATGGTCAAGATGGAAACATCAAAAGTACCACCACCCAAATCGAAGATCAGAACGTTGCGTTCTCCAGATGTCTTCTTGTCAAGACCGTATGCAATGGCAGCGGCTGTAGGTTCATTGATGATACGCATTACGTTCAAACCAGCAATGGTACCAGAATCTTTGGTAGCTTGACGTTGGGAATCGTTGAAATATGCAGGTACAGTGATAACTGCGTTTGATACAGTTTTTCCCAAGTAGGCTTCAGCGGTTTCCTTCATCTTGGTCAAAACCATGGATGATACCTCTTCTGGGGAGAAGACTTTGTTTTCTCCTTTGTAGGAAATTCTGATTTTGGGTTTGCCACCATCGCTGATTACCTCAAATGGCCAATGTTTCATGTCAGCTTGTACAGTAGCATCTTCAAAACGTCTACCAATCAATCGCTTGGCATCGAAGATTGTGTTGTTGGGGTTCATGGCGACCTGGTTTTTGGCAGCATCACCAATGAGACGTTCGGTATCAGTGAAACCGACATAACTGGGTGTAGTACGGTTTCCTTGGTCATTAGCAATGATTTCTACTTTACCGTGTTGGAAAACACCCACACAAGAGTAGGTTGTACCCAAATCAATACCAACAGCAGGAGTTTTGGCAGCCATTTTTCTTGATATGGAGTGTATGTGTAGTAAGGAATATATAAATAAGTCTTAAGAGTTTGACAAGAAGTCAGACTAGTATCTCGCGTAGAGTACAGGAACAAAATGGAAGATGCCGAATGTCGAGAGCGTAGCTGTAGCGCTTTCTGTGGTCGTGACTATAGTATTTAGTGGGTTTGGTGGGGAGGCGGTAGTCAACGTATATAATGAAATTCATTCACCGTTGTCG

>CL1854.Contig3

GGATTGTCAGATACGTTGCGAAGCGAAGTACAAGTCTTAAATTTTAACCAAATATTAACCTCGTGTTAAATTAACTTTTTTTAATTCAATCATACTTAGACAAGGAAATTAATTACAATGGCAACAAAAGCACCAGCAATTGGAATTGATTTAGGCACAACCTATTCATGTGTTGGAGTTTTCCAACATGGAAAAGTTGAAATCATCGCCAATGATCAAGGAAACAGAACTACACCAAGTTATGTTGCATTCACCGACACTGAACGTCTCATCGGTGATGCTGCCAAAAACCAGGTCGCCATGAATCCAAACAACACCATTTTCGATGCCAAACGTTTGATTGGTCGTCGTTTTGATGATGCAACTGTCCAAGCTGATATGAAGCATTGGCCATTTGAAGTTATCAGTGATGGTGGTAAACCAAAAATCCAAGTCAACTACAAAGGCGAAAACAAGAACTTCTTCCCAGAAGAAGTCAGTTCAATGGTCCTTGTTAAAATGAAGGAAACTGCCGAAGCATACCTTGGACAAACAGTAAGCAATGCTGTTATCACAGTTCCAGCTTATTTCAATGACTCCCAACGTCAAGCAACCAAGGATGCTGGTACAATCTCTGGATTGAATGTACTCCGTATCATCAATGAACCCACCGCAGCTGCAATTGCTTATGGCCTTGACAAAAAAGCAGTCGGTGAACGTAACGTTCTGATTTTCGATTTGGGTGGTGGTACCTTTGATGTTTCAATCTTAACAATTGAAGATGGTATCTTTGAAGTTAAATCAACTGCTGGTGATACTCATTTGGGTGGTGAAGATTTTGACAACAGAATGGTCAATCATTTTGTTCAAGAATTTAAACGTAAATACAAAAAAGAACTTACTGCCAACAAGAGAGCACTCAGAAGATTGAGAACAGCATGTGAACGTGCAAAGAGAACTTTGTCATCATCAACACAGGCCAATATTGAAATTGATTCACTCTATGAGGGTATTGATTTCTATACATCAATTACACGTGCACGTTTTGAAGAACTTTGTGCTGATCTTTTCAGAGGTACACTTGAACCTGTTGAAAAATCACTTCGTGATGCTAAAATGGACAAAGCTATCATTCATGATATTGTTCTTGTTGGTGGATCAACCAGAATTCCAAAAATTCAAAAACTTCTTCAAGATTTCTTCAATGGTAAAGAACTCAACAAATCCATCAATCCTGATGAAGCTGTTGCCTATGGTGCTGCTGTTCAAGCTGCTATTCTTCATGGTGATAAATCAGAAGCTGTACAAGATCTTCTTCTTCTTGATGTTACACCATTGTCATTGGGAATTGAAACAGCTGGTGGTGTTATGACTGCTCTTATTAAGAGAAACACAACCATTCCAACAAAACAAACTCAAACATTCACAACATATGCTGAAAATCAACCAGGTGTATTGATTCAAGTTTATGAAGGTGAACGTGCCATGACAAAAGACAACAATCTTCTTGGCAAATTTGAACTTTCTGGAATTCCACCAGCACCACGTGGTGTACCACAAATTGAGGTTACATTTGATATTGATGCTAATGGTATTCTTAATGTATCAGCCATTGATAAATCAACTGGTAAAGAAAATAAAATCACCATTACCAATGACAAAGGTCGTCTTAGCAAAGAAGATATTGAACGTATGGTCAATGAAGCTGAAAAATACAAAAATGAAGATGAACAACAAAAGAATGTAATTGCTGCCAAGAACAGTTTGGAATCTTACTGTTTCAACATGAAGAGCACCATGGAAGATGAAAAAATCAAGGACAAGATCCCAGATACCGATAAAACTGTAATTCTTGATAAAGTCAATGATACTATCAAATGGTTGGATGCTAACCAACTAGCTGACAAGGAAGAATATGAACACAAACAAAAAGAATTGGAAGGCATTTGCAACCCAATTATCACTAAATTGTATGCTGGTGCTGGTGGTGGTATGCCAGGTGGTCCCGGTGGTATGCCTGGATTCCCAGGAGCTGGTGCTGGTGGTGCCGCTCCAGGCGCTGGCTCAGGAGCTGGTCCAACTATTGAAGAAGTTGATTAAACTATTCGCTATTCAGCACATTCCTACATCATATGAACACCAACAGATGACTTGTGTTTTTTTTTCTTATGCATTTACTTTCTATTGATAATAAAAGCAATCATCAAATGTTTAAAAAAAAAAA

>CL1978.Contig1

TCTTTAACAAAATTTGTTGATTTATCAGTACAAGAATGTAAATAATAATAATAATAATATAAATACAAAGGAGTAAATAAGAAATATTTAAGTTGTATTATTGTTTTTTAACAACAATTAAACAACTTATTTTTTAGCACCATTTGGTTGTTGTTCTTTTTGTTGCTTGATGGCTTTTTTATCATCTTCACGAATGGCTGGTTTACCAGTGTGTTCAATCTTGATGCTTCGTTCATTTTCAACAGCAAGTTCAGCTTTTTTTGGTGCTGTTATTGTGAGTACACCATCTGATGACAATGATGAAGAAACTTGAGCAAGATCACATTGTTCTGGAATCAAATATTTACGTTGGAATTGACGTGAAATCCAGCCATGCTCATCTTGTTTTTCTTCATGTTTAGCTTCAACAGTGACAAATTTGTCAACGACCTTGACATTGATCTCTTCAGGCTTAAATTGCTGAACATCAAGGATAACTTGGAACTTGTCCTTGTCAGGTTTGACTGTTGATGTTGCTGATTCAGCATTGCGAAGAAGTTCAGCCCATGGACGAAGATAATTAGATGGACGAGCCAAGACATGATGTCTTCTTGGTGTTGGAGCCACGTAACGATGAAACATTTGTGGTGTCATCAACTGATCATGATTCAATCCAACTCCAAAATCTTGATCAAAAATATGATGAGGATGATCCAAATCTTCCCACCAGTTTGAGAATAACATTGGTACGAGAGACATCTTGATAAATTAACTTTCTTTAAATTAACTTGAACTTGATACTGTTTTAAAGATTAAAATAATCTTTTGATTTTTTTTTTTTAACAATTTGTTAATGGCACAAATTTATTAATTAACTTGTCACTTTTTTTAATCTCACTTTTATTTTTACTTAAAGATTGATTAATTGATTTTGTTATTATTTAGAAATAATAGCGTTGGTTAAGTTGGTTTTTTATTTGATTCGATGTGTTTGCTTTGATTGCTTTTATTCGACTGATGCCGTTATAACCGTGGTGCTCGTATTTATACAACTTTCTCGCCGGCCGG

>CL2504.Contig1

GAATAACTTGAATTTATGTGACAGAAAATTAAACAAATATTAATTAAGAAGGCAAAAGAATATCTATGAAGGATATATGAAACAAATATCAATAGGCACATAAGAGGGTAAAGAGTGCATAGGTACTTTTGATAGACAAGCTATTACATTTCAAAAATAAATACAAAAAAACTATATTTAAGAATTTATTGAATTGTTCAGACTCATAATAACATCCCATATCCAAATGTTCTTTTAATTGGTGTGAAGTAGTATCTTTCCCAATTTTGACGAGTAATAGCTTCATCACTTTTGGGCACTCCCACAAGAGTAACTTTAACTTTAGTATGGTCTTCTTGTTGATTGATAGTAACAGTAACAGTCGAATATAACCAATCAGGCCACTGTTTAGTTCTCCAATTCTTAACAATTTTTTTTGCCAGGACAAAGTTCTAAGAATTCGCCAGTGATATTGCCATCAAATAATTGAAATTTTCCACCTTTAGTTGCTTGAAATGTGACGGATTGTTGAGTAAAAGCTGAAACAAGCTCTGGTGTTGTGAGCACATTGTAAAAATCTTCACCAGAACACTGAAATACTACATCCATGACAACAGTAGTAGTATCTATTTTGGATTTTGACTTATCTTCTTTTGCACCATTATCAGAATTGTGAATATTTTTTAAAGATTCTTTTGACACTTTTGCTTTAGAAGAACTTTCAATATCTTTTTTGGGCAAAATCATGCCTTTTGAAAATTCTTCTTTAAGATCCTTTATATATTTTTCTAGTTGATCTCGGATTTTACTACGCCCTTGGTGGTAAAGAAAATCTTTAACTATTGTTGCTTCATTTGACGGTTCTTTTAATGAAATATTTATCTCAATTTCAGCTACACTATTTTCTTCACTTAAATTTGGTATGTTTATTGTGCCCTCTATCTCTTTTGCAGTACCACTTAAATGTCCTTTCCAACTTAAAGTTAAATCCCACTCATAAAAGAATATGAGCTTGCCCTTTCTATTATTTGCAACAGCTTCTCCTTCACAACTATCGATCTTTACAATCTCACACTTAGCTACATCATTTTCTAATACCATACCAACTAATAAGGAAGCAAGTTTGTCTTTAGACCACTGACATGCATTTTTCTCTGTCCAATGCCAGTTGTTTACATTAGTGGCATCTGGGCGTTCTTCAACAATCCATCGCGGATCTCCTTCACCCCATTTAGCCATTATGAATTAAAATTGAAGAAATTTAACTGAAAATACTCAATCAAAAAATAACAAGACTATTCTAGTAAATGAAATGTAGTAAATGTTCACCACAAAAATACAAAATAATGTAAACGATTAACGATGGAGAAACCCGTGGTACACTCGGTACAGAACGGCAGAG

>CL2603.Contig1

TTTTTTTTTTTTAGTTTTTTTTTCTTTGAATTTTTTCAACTTATAAATATATCTTATTTATAAAAAAATAAATGCTTTCATAAAAACCTCAAGGGCCAGTAAACGACACAGCCGATGGCCTTACCTCAGTCAAACGCGTTCAGACACACGCATATAAATTTTTTTTTTTCAATTCAAAAAGTATATAGAAAAAAAATTAAATTTCTTAAAAAATACAAAAATCTTTAGTTTTCAGATCTCAATCGATAATAATCACTCAATGTACGTTTAGCTCTTATATCACGATCCTCGATAAATCGATAATTTTCAACGCAATAACACGGTAAATCGCCATCCATCTGTATAAATTTACTTTCGTAAAAAACCATTATATATATTTGTTATTAAAAATTTTATAACGCTGTTATGTTTGACGAAAAAAAAATTATATTAAATTTCAATTCATTTTATCATGCAATAAAGTACGACATTTTCCAGTTGACAATATTTACTGTTCAATTATTTATGGCTAAATTAATTTTTTTTCATACAAGATAAAATGAATGAAATGATAAAAATTTTAATGATTAATTACCGAGGTAGAAATGAAAAATAAGAATTAATAAATTGGCTCGCAGGTGAGCCCGCGAGCCAGAAATTTTTACTTCTTTTTTTTCGGGTTGTACGCTGTTTTAAAAAGACTAGTATATTTTATAATAATTTAAATAGAAACAAATATATTTCTAATGAAAAAAAAAAAAACTAGAATAATAATAACGTCTTGATAATAAAGACGCTAACCTTATTGTTTCCGGTGTGTGATTTTTTCTAGTATACTTTGTTTTTAATTTGTTTTTTGTTTTGTCGTTTTTATGACTTGATGATATTGTTGATGATGCTGTTGTTGTTGGTTTTCGATGATGATGGAAGCTGATGAATTATCATTGGAACGCAATTATGCTTAATTGTTGTAGGTGATTCTGGATGTACAGCAATTTTCACCAGATGAAATAACAGTCAAGACAATTGATGACAGTGTTATTGTCGAGGCTAAACACGATGAGAAAAAAAGACGAGCACGGCTTCATCAGCCGTCACTTTGTAAGACGATATGTGTTACCTGCTTCACACGATGCACTTGAAATCACTTCAAGTCTTTCCTCAGATGGTGTTCTTACAATAACTGCTCCAAAGAAGGTGAGACTTTCACAATAACTATATACTTTAAAGCTAGATAAAGCTTGCTTTTTTTTTTATCTAACTTGACTTATTGTTGACATGTGTTAAGTTATATCGAACCACTGCCTCTACTCAAACTCCCACCGCAGACTCCCGATCTCAACGGAGAGATCCACTCTCCGCTGGAATCACTTGATCCAGTTGAGTTGATAAAAAAATTGTTCTTTTTTTTGGTCACAAATGTTTATTTTTTTTATTTTTACAAGTAACTGGATAATTGATGATTCTGGAGCTGGACACCAAGCTTAAACTATTAAAAGCTCTCTTTAAACATAATCAATTGTATGATCAGTTTGGTCAATAGTATAGTCAGACCAGGATGATGTCTGACCAACACAAGTGGATGGCACAAATTGATGAGTACGGATTGACCAAGCACCAAGCCCGGGGGCTATTGGGTAAATGTCACGGTCCGCACCGTTTTCTCCAATTCTACATCGTAAACCGTATTATTTTAGTCACAACAATATTTAAAAAATATTAAAATATTTATTTATAAGTTTTTTAAATTTATCAAGAGATGAAATCACAACGATGGTTTGTTATTGAATGCTGC

>CL2817.Contig3

TGTAGAACGTTCTAGAATATTCCCTTAATCGATTCACGTTTGCCGGCTCTCCGAATCGCGTGTGCTGTGCCAAGCATTCCGTCGGTTTACTTAGTTATCAATTCAATCCTCTTTAGACCGCGCGTTAAAAGCCGATAATTTTTTTTTATTTCTCATTCACGGCGTGAGCACACCTACTGATCGACATATCCACGTGATAATTTCTCACCGTACGATTTACACAAATAAATGGCCGCGATGTCCGTAATAGGTATAGACTTCGGCAATGAATCGTGTTATGTGGCCGTGGCCCGGGCCGGCGGCATCGAAACGATCGCCAACGATTACAGCTTGCGGGCCACGCCGTCGTGCGTTGCCTTTAGCCCCAGGAACCGTATCATCGGTGTGGCTGCGAAGAATCAGATGGTTACCAACATGAAGAACACCGTGCATGGCTTCAAGCGATTGCTGGGCCGGACCATCGACGACCCGTTCGTCAAACAAGAGCTCAAACACCTGCAGTTCGGCGTGGTCAAGGGTGACAACAACAAAATCGGAATTAACGTCAACTATCTAAATGAACAACAAACATTCAGCCCGGAGCAGATCACCGGCATGTTGCTGACCAAACTCAAAGAGATATCTGAGTCATCGCTCAAGACTAAGGTAAATGATTGTGTCATTTCAGTTCCTTCTTATTTCACAAATGCCGAGCGCAAGGCATTGCTTGACTCTGCCTCTATTGCTGGACTTAACGTCCTGCGTCTGTTCAATGAAACCTCTGCCACGGCACTGTCCTATGGTATCTATAAACAGGACCTTCCTAACCCCGAAGAAAAACCCCGTAATGTTGTGTTCGTGGACTGCGGATACACTTCCCTCCAAGTTTTCATCTGTGCGTTCAACAAAGGCAAATTGAAAATGTTGGCTAGTACGTTTGACTCTCAATTGGGCGGTAGAGAATTTGATTACATTTTAGCTGAACATTTCAGTAAAGACTTCAAGTCCCGTTATAATATTGATCCAAGAACTAATGCAAGGGCGTTTTTGAGATTGTTGACAGAAGTTGAAAAGATTAAGAAGCAGATGTCTGCTAATTCCACTAAGTTGCCAATGAACATTGAGTGTTTCATGGATGATAAAGATGTGCATGCTGACATTAAAAGATCAGAATTTGAAGAATTAGCCATGTATTTATTCAATAGAGTTGAAGTCACATTAGAACAGTGCCTTAAGGATTCTAAGCTGAGTAAAGATGACATTTATTCTGTAGAGATTGTTGGCGGCTCCAGTCGTATTCCATACATCAAGAACTTAATAGAAAAAATCTTTGGCAAAACACCTAGCACCACTTTGAATCAAGATGAGGCCGTGGCTCGAGGATGTGCTTTACAATGTGCAATGCTATCACCAGCTGTGCGAGTAAGAGATTTCAGTGTTACTGATATTCAAAGTTTTCCTATTGAACTCCTATGGGATCCAGCTGATAACTCGGATGATGGTCGTGCTGAAGTTTTCCCTAAAAATCATGCTGTTCCATTTTCTAAAATGTTATCTTTCTACAGATTAGCTCCATTTACTGTTAAAGCTCATTACTCTGGCCCTATCCCTTATGCAGATAGTTATATTGGTCAATTCACTGTTCGTGATGTCAAACCTACAGCTGACGGAGCAAGCCAAAAAGTTAAAGTTAAAGCCAGAATTAATTTACATGGTATATTCAGCATTAGTAGTGCTACACTTTTAGAGAAATCTGAATTAGTAGAAGAAGTTCCACCTTCTGAACCTATGGAATCAAATGAAACTGAACCTCAGCCAACTGAACCTGAAGAAAAAAAAGAAGAAAAGAAAAAATCTGTTACAAAAACTATTGATTTACGTATTGAATCTTTGACTCACGGTTATTCGACTATGGATTTAAATAACTATATTGAGCAAGAAGGTAAAATGGTTGCGTCTGATCGCCAGGAGAAAGAAAGAATTGATGTTCGTAACAGTCTTGAAGAATACATTTATGATATGAGAAGTAGAGTTTCTAGTGAAGAAGATTTAGCCTCCTATATAGTAGATGCTGATAAACAAAAAATTGTTAAACAGTTAGAAGACCTGGAATCTTGGCTGTACGAAGAAGGCGAAGAATGTATTAAAAATATTTATACAGAGAAATTGGATTTGTTAAAAATGGTTGGTGAACCCATCAAGAGACGTAAAGTAGAGTACACAACTTTCCCATCTGTTAGAGATCAAGCTATCCAATTAATTTCAAAAGCTGAAAGAGATATTGATGCATTTCGTAAAGGAAGTGAGCAGTTCAACCACTTAGACAGTGCTGAAGTAGACAAGTTGTCTGAAACACTTAATAATGCCAAGAGTTGGTTGGACGAAAAGACAGCCAAAGTAACTGCATCTCCTTTATATAAAGACATTCCGATCAAGTTAGATGAATTTGTTAGAGAGAAACATAGTATAGAAGAGAATATAAGCAAAGTTTTATATAAACCAAAGCCAGCACCTAAAGTGGAACCTCCGCCACCTAAGGAAGAAGAGAAAAAAGAACCAGAACCAATGGAAACTGAACAGCCAGTCGAAAATGGTAAAGATGCTTAAAAATGTTACTTTTAACACTTTATTTTTTTTTTTTTCTATTGTTTAATATATTTAATTTTTTTATTTCAAAAATATGTCACCTTTATATTTGTATGTTTGTATTCACAAATATGAAATAATTTTGTAAGCAACATAATGTATTACAGTTTAATAGATGGAAAAAAACAAATTTACTAGAACAATATAGGAATATTATGTTTCATTATTTTTCCTGCTCCTGTGTTAACTATTAATTAACTAACTTTGATTAAAGAAAATAAAAAATTGTTCATCACGTTTAATGATAATTAATAAATTGTATTAATCTGTCTAATATCACCATTGTTCACAGCTGATGTACCTTAGTTAAAATTAATACATTAATCTTTCAAGTATTTTTAAATCTTTATAAGTATTTTATTAACTGTG

>CL3029.Contig1

CTCCCGGCTTCATAATCTACACACTCTACTCGTTCTGTGTTGTGGCGTGGTGCAGTTTTACGAATAATTTTTGTTCGACTTTCAGTGGTTAAATCTACTTAATTAAATTAAATTTCTGTTCTGTTGAATTATTTAAATCATTCAAGATGTATCGTATCTCTGCCGCTCTTGCTAGAAACAATGTACCCCAATACTTTGCCAGAAACTATGCCAAAGACATTAGGTTTGGACCTGAAGTGAGAAAACTTATGCTTGAAGGAGTTGATATCCTTGCTGATGCTGTTGCTGTAACTATGGGTCCAAAAGGCCGTAATGTCATCTTAGAACAAAGTTGGGGTTCTCCTAAAATTACCAAAGATGGTGTAACTGTCGCCAAAGGTATTGAATTGCAGGATAAATTCCAAAACATTGGTGCCAAGCTTGTTCAAGATGTAGCAAATAATACCAATGATGAAGCTGGTGATGGCACAACAACAGCTACTGTATTAGCACGTGCTATAGCTAAAGAAGGATTTGAAAAAATTATTAAGGGTGCTAATCCTGTTGAAATTAGACGAGGAGTAATGTTAGCTGTTGATACAGTGAAAACACATTTAAGTACATTATCAAAGAAAGTTCAGTCTTCAGATGAGATTGCACAAGTTGCCACAATCTCTGCTAATGGTGATATTAGTATTGGAAAACTTATCTCTTCTGCCATGGAAAAGGTAGGAAAAGATGGTGTAATAACAGTTAAAGATGGAAAAACCTTGGAAGATGAATTGGAAGTTATTGAAGGTTTAAAATTTGATAGAGGATACATTTCCCCATATTTCATTAACTCTACTAAAGGTGGCAAAGTAGAATTCCAAGATGCTTTAGTATTATTCAGTGAGAAAAAAATTTCATCTGCTCAATCATTAATTCCTGCTTTAGAATTAGCCAATGCACAACGTAAACCACTAGTTATTGTTGCTGAAGACCTAGATGGTGAAGTTATTGGAATGCTAGTTTTAAATAGGTTGAAAATAGGTCTAAATGTTGCTGCTGTCAAAGCACCTGGATTTGGAGATAATCGTAAATCAACATTAACTGATATGGCAATTGCAACTGGAGGCGTTGTTTTTGGCCAAGAAGGCAATGAGTTGAAACTTGAAGATATTAAAGCTAGTGATTTTGGTGAAGTCAAAGAAGTGGTCATCACCAAAGACGATACTTTGCTTTTAAAAGGTAATGGTGTACCATCTGACGTGGAACAAAGGGCTGAACAAATTAGAGAGCAGATTAAGGATACTTCTTCTGAATATGAAAAAGAAAAACTGCAAGAACGTCTTGCAAGGTTAGCGTCTGGTGTTGCTGTATTAAAAATTGGTGGAAGTAGTGAAGTTGAAGTCAATGAGAAGAAAGACCGTGTTACTGATGCTTTAAATGCTACTCGTGCAGCTGTTGAAGAAGGTATTGTTCCTGGAGGTGGAACTGCTTTAATTCGTTGCTCACCTTCATTGGATACAATTAAAGTGGCCAATCCAGATCAAAAAATTGGTATTGAAATTGTTCGTAAAGCATTAACAATGCCTTGTATGACAATTGCCCGAAATGCTGGTGTTGATGGTAGTGTTGTGGTTGCTAAAGTATTGGAAGGTAAAGACAGTTTTGGATATGATGCATTGAATGATGAATATGTTAACATGATCGAAAAAGGAATCATCGATCCAACCAAAGTTGTCAGGACAGCATTAACCGACGCTGCTGGTGTCGCGTCATTGTTGACTACAGCAGAAGCAGTTATCACTGAATTACCAAAGAAGGATGAGCCATTACCTGGTGGTGGTATGGGAGGAATGGGCGGCATGATGTAATAATTTGTTTTAGAAATCACAAAGACTGACATCGCGCAAGTGGCATAAATATTTTTCTATCCTAGTTATGTGTTGTTCTCGATGTTTTATTTTATAAGAATCAAATTTATTAATTATTAGTAACATTATTAGTTTTTTTTTTATACATATTTTTTAAATTCATGTGTATGTACAAATGGATTGTAAGTTTCATTAGACACATGTTAAGAGATATTTTGTCTATTGCATAGATGTTAAAATTGGAAATTGTACAAAGTGTTTTAGGTCATATAAAACTCATTCCAAAAATCAAAAAAAA

>CL3320.Contig2

GAGCAAAGCGTTTTCCAAACGCAATCACTGCCGCTGCGCGAACGTTCTAGAAACTACAAACTCATTCCGCATTCGATAAAACGTCGTACTTGTCGGCTCGGCTTGGCCTTTCACAATCCGCTTTCGTAGCTACGCAGTCGTTCTTTACCGGACGCGCATCACCTGTTTCGCGTGCTAACAACGATCGTCGTCAGATAATAGAACATAAGTACTGTGAACGTTCCGAACGGATCTAGATTCTTCAAGAAACGCCGAAAAGTCTTTTAATTGTGGTCCGGTATCATAGTTTTGACAAGATTAACTAGTTGGAGTTAACCATAACAAAAATGGTTAAAGAAACAACTTACTATGATATCTTAGGTGTTAAACCTAATTGTGGGAATGATGAGCTTAAAAAAGCATATAGAAAATTAGCCCTTAAGTATCATCCGGACAAGAACCCAAATGAAGGTGAAAAGTTCAAACAAATTTCCCAAGCTTATGAAGTATTAACTACACCAGAAAAACGTCGTTTATATGACCAAGGTGGCGAACAAGCACTTAAAGAGGGTGGAGTGGGTAACTCATTCTCTTCACCTATGGATCTGTTTGACATGTTCTTTGGTCAATTTGGTGGAGGTGGTGGTCGTGGCCGTGGTCGACATGGACCTCAAAAAGGCAAAGACGTTGTTCATCAATTAAGTGTTACTCTTGAGGATCTTTACAATGGCTGTGTACGCAAATTAGCCTTAGAAAAAAAATGTTATTTGTGACAAATGTGAAGGTAGGGGTGGTAAAAAAGGAGCCGTAGAGCAATGTCCAGGATGTCAAGGCAGTGGAATTCAAGTGCAAATTCATCAGTTGGGACCTGGTATGATACAACAAGTGCAGTCCATGTGCAGTGAATGTCGCGGACAGGGTGAACGCATCAATCCCAAAGACAGATGTAGAACTTGTAATGGAAAAAAGGTTACTCGAGAAAGGAAAATTTTGGAAGTAAATGTAGATAAAGGCATGGTTGATGGACAAAAGATAACATTTAATGGAGAAGGTGATCAAGAACCTGGACTTGAACCTGGTGATATAATTATTGTGTTGGATGAAAAAGAACACCGTGTATATAAGAGAAGCGGATCTGATTTAATTCTTCGCTTGGAAATTGAATTAGTTGAGGCATTGTGTGGATTCAAGAAAGTGGTTAAGACTCTAGATGAACGTTCTTTGGTGATTAACTCTGAAGCCGGTGAAGTTTTGAAGCATGGTGATGTCAAATGTGTGATGGGTGAAGGTATGCCTCAGTATAAGAACCCATTTGAAAAAGGTCGCATGATCATTCAATTCTTGGTGAATTTCCCCGAAACATTGTCACCCTCCAAGGTGCCTTTGCTTGAAACGTGTCTTCCTCCAAGACCAGTTGAAACTATTCCGGAGAATTTTGAAGAAGTTTCGCTTATAGAGATGGATCCTGAATATGACTCCAGAAGACAATCCAGACGTGAGGCATACAATGATGAAGATGGACCCACCAGAAATGTACAATGTGCTTCACACTAATGTTTTTCACTTCTAAAAGTCCATATATTTTACAGTACTGAGTTATCTGTACTCATTCCGTAATAAGACACTGAGCAAACAAAATTTGATATTCTGTATAAAATTTTAGATGTCATATACAGTATTTCTTTCCTTATATATTTACTATTTTTTTTTTTTTATTACTCAAAAGTTAAAGGATACATTTAATTTAATTGCTTTCTGCTGATCAATACATTAGTACACTCCATTTTTAGTTACTTGTTTTTTATTTTATAAAAAATGATTAATCATTTCTATTTTACACAGAATTATTATTTTGTATTAATTCTTAATCCCAGTTCCCTGCAATTCTGTTTAGTCATTGTTTCCTATTTTTCTTTTGATTTGAATATGGGGTTATGATTTGAGAATTCACTTTTTGTAATTAGTGGTATTTTTTGATTTTTGATTAGTTTTTATCAAGGGAATTTGTTGAAAGATGATTTTATGTTTGTTTAATATTTGTAAAACAAGTAAATTTTAGGCGTATTCTATTTCTAACTTGTCAAGAATCTAGTGAACAGATTAAATGAGAAAGAGAACAAATTCAAATTTACAATATATTTCTTATAGCATTTCAGGTTGGAAATAGAACAATTTTTTAATAAACCAACAGGGGTTCTTTAACTATTAGACGTCACTGCTTTCCACCCAAGCAATATAAATATTTTGTACATTTTTATGGAATAGTTATATTGTTCTGATTATACTAATATGAACAATTTTTTGTTGCATGTAAGTTTGAAAGTACAATACCAAAAAAATTAAAGAAATTGATAATTTAAAAATAAATTAATTTCTTTTATTTTTGAATTGTTTGATATCTTTTATTAAATAAACTTCTTGTAATACGCCATACGTATAATTAATGTTCCCCGTTGGTTTTGAAATAATTGATGAATAAATATAATAACTCAAACTTAATACAAGATTAAAAAAAAAAACTGATACTTACACTGAT

>CL3343.Contig1

ATAATAATAATTTTTTTTTTAAATGTTTTTATTCGACAATAATTTAATCAAAGATGACGTCGATGGTCACATGAAGTGTGTTGTTACACTGCGGAATCACGGCTTCTCTTATCAAAAAGTACTTTTTTTTAATTTTTTTTTTTTTCTTTTCTCTATTTTCTTCCAGCAAAAACACACACAAACACATTGTTTATTTTCCCAGTAACGTGTTTCGAAATACAACGATAAATTGAATGACTATTACTATTGATTATCGAAATTATTAATAATTAATAAACATTAATATTATGTAGATAAAAATCTGTCTCTAAAATTTTAAAGGTCATTAAAATCCGCATAGAATAATTAACGTAATGACGTGGAAGAGATATTTGTTAATCACTTATTGGATCAACAAAACGCCATATCGATAAACCCCCTCAATTTTCAATCTGTCATCAATTTTTTTTTTAACCCAATAAAATATAAAATAACAAACAAAATATATATTCAAATAATGAGTCATATTAATGATATTTTTATACTCACAAGTGTTTTTTTAAAAAATATGTAATATTTTATTTTCAGTAAAAATATGTGGGCAGCTGTGAATCTTTTTTATTAATAAATAAAAAAGATGCGTCACAACGCTATTTCTATTTAAAGTAACCCAAAAATCAAAATAATATTATGTATTATTCGCAGTTCTTAATAAGCTTTTAAACGTGTCAGTTGTGTTTGTTGCAATTATTTATCTTGGATCTTTTAATTCCCATATAATAAAGAAAAAAATTTTATCACGTATGTAACGATGAGTTTAATATTAATTATAATTATTTTATAATTATTATTATCAGCCAAGCTTTAATATTAATTTTACGAATAATAATTAAAACTTTCGTATTTGTACTTAACACAGTTGTTATTATCATTCTACGATATTTTTCTTAATCCTAATTGATTATTTTTCAAGATTTTTTGAAAAGATGGTCAACTGCATCTTGTCAACTGTTTACCGAATTCTAAAAGTATTATTAACGTATTAATTTATTATTAATATCATTTACATAAATGAGGCTGACAATAATTTTTAATTAATTAACAAGTTAAGTTTTTAAAAAATTAACGAGTCATGATGTTGATAAGACCAAACGAAAAATTAAACTTTCCAGTTTAAATTTGTTTGTTTAAAAATCTCCTGGAAAGCTCAATGTGATCGATCATGTCAATAAACATGAGTGTCAATAATAAATTGGATTAACTTGTTAATTATTAATCCTTAAATTGTTTTTTATTTTTTGTTAAAGTGCAACGATATTAATTTTTAAATTTTTATACTCTCATGTTGTTTAATAATTCGTAAAATTATTCATGCATATTTACAAATTAGAATCGGTAATTAGATGATAAAATCCCCATTTTAAAACCACAATCAACAGTGGTCTTTTTTTTTACTAAAAAAAAAAAGAAACGGATTAATTAATTAATTAATTTTAATATTGTATGTTGTTGATTATTTAACAAACAAGTATTTGGTTTTTGTTATTTTTTTTTTCTTTTAACTTTCTTATACAATTAATTAAAAGCCTGAGTATTCCTATATAATTTACCGATTTTTATTCGATGCCAATAAGTTGTGTGAGTTGATTAATATTAATTAATAGACGGAAGAGATATAACATATTCTAAAAAGGTACTAAAGTACTTGGTATAATAGCTTGGTGTTTCCTGACATTTTGGTAGACTCAGTTTTCTTTTTTAAATTTTAAATCGTCCACGATAATTTAAGAAATCCTACTAATTAAAAAATTTTAATAAAAACAATATTAAATTGTTGATAAATAATTAAAAAATTAATAAATGAATAATAAAAATTTATTAAAAATTTGCATGTTTTACGAAGTTGTTCCCATCTACCTTGTTTGAGCTAAGAGTTATACGCACGAACTATCTTTATTGAAAAAATTATTAATTTAAAATAATAAAATGAGATATTAATTTGTCAGTGAAATATTAATTATTAGAAATAAGATAATGATAGTTTTAGCTTGCTCACGTCTACCGATACCAATATTTTTTATTATAAATCTTATTGAATTATTATATTAATAATTAATTGTGCGATCTGTGTTTGACGTGATATTGACTAACTCATCAATATTTTCTCCCCTTAATCTATAGATATGACTCAACACTAGATCTTGATTATTTTCATATTAACTTGATATTGTCATTCTATTATTTAAATTTTAATTATTCTTAAACGTTCCCTCAAGATCTTATCCATTAATTTTTCATTTGATTTTTGCTTATTCATTTTTCTTGTTTTTTCTCTTCAAACCTAAACACTAATTTTAGTATTTTTATTTATTATTATTAAATTATATTTAAACTCATCGTTAGACGTGTTGGGGGAATTAATTGATCTTTGATTAATTACCACATATTTTTATTGAATATTTTTTTATTTTTTTATTGGGATAACAGAATGTCAATAGTTCCAGTGATGTTTCGCGACTGGTGGGATGATATTGATCGTCCCATGTCAAGATTAATTGATCAACATTTTGGTAGCGGTTTAAATCGTGATGATTTATTGACTGGTTTATCATCATTGAGTCTTGCTAGACAGAGGCCAATATTTACTGGACCTGGTTATTATCGTCCATGGACTAATATTGCAAGACAAAATTCAGGGGGTGCAAGCACTATTCAATGTGACAAGGAAAAATTTGAAGTAAGCTTTATATATTATTTTTATAAACTATATATTTATTGTTATTATTAAGACATTTCAAAAAGACTTTTGTTAATTATTTTTGTTGCTTTTATTATATTTTTCTTTTTACTTTTATTTCAGAGACAAATTTTTTTATACTTTTGTTGATAAGTATTGTGCATATTGAGTGATAATAATTTATAACTTCTAACACACAGTGAATAAGTAATTTTTTTATTATTCCGAGTATAGTAACGGTGATGAGTAAAGAGGTGGGAAAATAATTTAATTTGAAAATTTTGCATTTAAATTAAATTAATAAAAA

>CL363.Contig1

GTTCATTTTAAACTTCAAAATTTTGCGTTAATGATTGAGAACTGGAAAATAAATCTTAATTCATTAAATTAATACTACGATCATTGTTCTATAGTTGTATTCATTTCAAAGCAGTCAGTTATAATATCTTATTCATAGTTTTTTCTGATCGGCGATTGTTACAAGCATCTCAATCTTTGTATACAGTATTTAATTAACTTTATATAGGTTCATAGAAGTATAAAATTATACAATATTATCATCCATAAACCATAACTTAATAATACCATTAATTTGAACCGCTATTTTTAATTTACCATTGACTATTTGATATATTAACATCAATAATATTATTTATTTTTTGATTATACTTAACTATGGAGAGCAACCGAGATGAGGCATCTCGCTGCATTGATATAGCTAGGTTATACTTTAAAAAAAAGGATTTGGATAATGCTTGGAAATTTTCAGTTAAAGCTAACAAAATGTATCCATCATCTGAAACAAAACGTTTAATATCAGAATTAAAATCATCAATGAAAAACTCAAACCAACGGTCAATGAATCAAAATCAATCTGATCATTCTGACTCATCTTATAATGGTGATGATTCCAATTCATCTTCCAGTACTGACTATAAAACTAAAAAACAAACAAAAAGATCAAGAAGAAGCCGCAGACCAAGTACTGTTTATTCACATAAGATGTTTAAAGAGATAAATAAAGATGAATCTTTTAAGTGTCTTGAAAGAGCAGAGCAATTCTTGAAAACTAAACAATTTGATTTAGCTGAAAAGTTTGTTCTCAAATCAAAAAAGCTTTTTCCAATACCTAGAGCTGATGAACTCTTACAAGAAATTCAAGAAATTAAAGGTTCCATTCATCCTGAATATACAGAAGAACAGGCAAATATTGTCGAAAAAAGTACTGGTATTGAGAACCAAAGGGAAATATTTTGGAACTGGACATGAACTGGAATCGATAATTTTCTAAGAACCGACCCATTCCTACTATCACCATCAATGTCCAATAACCCAATAATAAATTTAGTATAAAAATAATAGTATCATAAATATTTATTTTTATTACAATAATTAATAATTAGAACATCTAACATAATTTTAGTCTATTTTAATTTAAAAATATTTTGTTTTTATATTACAAATTTACAACTGTGCAGTAATATAGATGTGTCCTGCTGATGCGTCGAATGTAGCTCGCATTTAGAACAGCATGATAAGATAGGATCTGACAGAAATCGCTCAAACTTGTCGCACTAGCTAGAATATGGCTGAGCTTAGTGGCTTACTAAATAACAGCCTATTAATAATCTTTTCACAGATTTTTGTTTACATAATTAACAAAAATTAAAAATACATTAAAGTAGTAAATACTAATTAAATACATCTAGGTAAATTATATATTTAAATGCTTTAATAATAGCTCAAAAAAAAGAAGCTAAGGGGAGTTGATTCTCCAAGCACCTCCCCCATTAATGCCACTAATATCAAGTTATCGTATTATTTTATGTTTATGCACAAGTGAAAAATAGTGAAAATCACTATACAATGTTAAACATTAAACGTACTGCAACTGTTCCTGAAATAAAAAAAGCATATAAGAAATTAGCTTTGTTATTACATCCAGATAAGAATTCTGCACCTGGTTCAGGAGAAGTTTTTAAAGATGTTAGTAATGCTGTTGACACATTATGTGATAATACAAAACGTCAAATATATGATCAAACTTTAATAAAACCTAAACCTTCTACTTCTAGAACTACAAATTATCCTCAAAGTTCCTGCCGATATCAATCTCCTTATTACCATACAAGAAAAAGAAATACTTATACTTCTTATACATTCTGTCGAAGTCCATCATTAAAAAGCTCTGACTTAGATGACGATGATTGTGATGTTTATGATGATTATGATGATTATATTAATGCCCACAATGAGTTCAATGAGTTCAATGGTTTTGATTATTATGAAATGTGAATATTATATTGATTTTGAGATTAATTGATAATTTATATTATAATAAATAATTGTACATATATTTTTTATCTTTGAAGCTTAA

>CL4130.Contig1

GGGAGTATAATTTTTAACCTCCCCTCTAGCATAAACCACTGGATATAACTTGTAAAGGCTTTGTTATTGAATAAAAATTATAAATTTTCAAATAAACAAAATAAATAATCACATTTAATCCATTTATAAAAGATTTTTTTTTAATAAAACATTATTCATAGTACGTTATTAGTATAATAGGTATGTAAGTAAAATATAAATAAATTTATTGATGTTCAACTTTAATCTCAGTAACTAAAGGAGTTTCATCGTTTTTCACAGGTTCTGCTGCTCTCACTGCTGGTGTTCCTGTTTGAATAATTGGTACACTACGTTCACCTGTTACTTGCTTTTCCTTTTTAGGAACGCTAACTGTTAGTATTCCATCCGAAGACAAGCTTGAAACAATTTGTTCAATATCCACATCTTTAGGTAATAAATATCTTCTTTTAAATTGCCTGGATATAAAACCATGCTCGTCTTGTTTTTCTTCGTGTTTTCCTTCAACAATCACTGCTCCTTCTGATGTTTTAACTGTAATCTCGCCGGGTGCAAATTGTTGGACATCCAATATAACTTGAACTTGTTTGTCATCAAACTTAAGGTTGGACGTACCACTATTTTGCCTGTTAAGTACACCTTGCCATGGTCTATAGTATGTAGCTGATCTTAGAGAAGGCGAAGTAAGAGTAGAAGTTAAATTAGACAAATCATCTCTATGCAACCCCAGACCAAAGTGTTGGTCCAATAATCGTCTTGGTAATCGTTCACGTTCAAAATCTTCCCACCAATCTCGGAAAAACAATGGTACCATAGACATTTTAGAATAAAATTTGGTTTCCTGTGCAATGTACTCAATAAAAAATAAAGATGTAACTTCAGTGGATTTTTAAGTTTAACTC

>CL4328.Contig1

GGTTAGTTGAATACTGTAGTAGTGTTTATTGTGCAATGCCATTCGATAAACTCTTTCGGTTCAATACGCCAATACGCCGTCTTTACCGAAAATAATATTTCCAAATACGAATCCGTAACAAGTGACCATAATATTTTTTAAAATTACGTGCTACACAATATTTTACGCTTGTAGTTAATTTAAGAACAATTTACTCGTGAAACGACCAAATTTACTATAAATCATATGATGGGAAAGGACTATTACCAAATCCTAGGAGTGTCAAAAGGAGCAGCAGATGATGAAATTAAAAAAGCTTATAGAAAATTGGCACTCAAATACCACCCAGACAAGAATAAAAGTGCTGGAGCTGAAGAAAAATTCAAAGAGGTAGCAGAAGCATATGAAGTATTGAGTGATAAAAAAAAACGTGATATATATGATAAATATGGCGAAGATGGTCTTAAAGGAGGTGCTGGCCCAGGAAATAATGCTAATAACTATTCATACACTTTCCATGGTGATCCTAGAGCAACATTTGCACAGTTTTTTGGCTCATCAAATCCGTTCGGAAATTTGTTCTCTAACAGTTCAATGTTTGACGACGAAATGGACTTTGATGATGGTTTTATTCGAATGTCACATGGTCCTCCTGGCATGGGTGCTTTTAGGTCACAGTCATTTAATGTTCATGGTTCACCCATGGGACGAACTAAAGAAAAAGCTCAAGACCCAGCAATTGAACATGAAGTATATGTGTCATTAGAAGATATAAGCAGAGGGTGTACAAAGAAAATGAAAATTTCTAGGAGAGTGCTCCAACCAGATGGAACTTCTAAAAAAGAAGATAAAGTGTTAACAATTAATATAAAACCTGGTTGGAAATCAGGTACTAAAATAACATTTCAAAAAGAAGGAGATCAAGCGATGAATAGAATACCTTCAGACATAGTTTTTGTAATAAGAGATAAACCTCACCCAGTTTTCAAACGAGATGGAAATGATATTAGATATACAGTTCCAATATCTCTTAAACAAGCACTATGTGGTGTAGACGTTGTAGTTCCTACCTTAACAGACAAAAAAATTAAATCTCAACCTAAAATCAGAAGTTGTAAAACCTACAACGGTTAAACGATTTCAAGGTTATGGTCTTCCATATGCTAAAGAACAATCAAGAAGAGGAGATTTATTAGTATCATTTGATATTAAATTTCCTGAAACAATTTCACCTGCTATGAAAGCTGTTTTGTGCGATACGCTACCGTGAGTTGTTTAAATTGGTTCAAAATTGTATTTGGGCTTTTATTTTCTAATAAAAATAATTTTTTATATATATAAATATACATAATATATTATATTTATAAGATATATTATGTGTTTTCTTAATCTTGTTCTATTTTTTTTTTCTAACATCTTCGTTGTTTGAAGATAATAATAAATATTCCGAAATAAATAATTTAGGTATTATTTTGTATATAGAATTTAACATACATCATTTGTTAATAAATTGAACAACTTTCTGGTTTATTTAATATGATTTAAAATTTTTTTTTTTTAATATTCCTCCTTGTTATTTAATATGTGCGCCAAAACTTGTAGTTCTATTTATACATTCACTATATATTTTATTTATAGATATTTTCATGTAAAGTTACAAGAAAATATTGGCTCTTGTGCCTAAATGCCCTACTGGACAAATGTTGTTTTAATAATTATTGTATATTTATTACTTACATAAATTGTAATTATATTATAACAATAAGCAAACTATTTGTTTAAGTTATAAATTATTCGTTG

>CL5221.Contig1

GCCGCCCCATTGCCGCCGAGCGCTGACTACGGATGTACTGACTACTGGGGACTGGAAGCCATTTCCGTGAAACCGCGTTTAACGTAAACTTGTGCATCGCCTGTGATCCACAACGTGTTTATTTTGGCGCTTCTAGAACATTCCCAATCCTGAGACACTACATGTGATTTCCAATCGATCGTCTACCAAAGATTACTGTAGAACTTAAAACCACATCACAATGTTGAGCGCCGCTAAATATGTTGCACGTCGAGCTGCAGAGCAGTCGCTTCTTGTTAAACAAGATATAATTTCTAAAGCTTTATGTCTTAGCCCATTTCAAACTCGACAATCATCATCGAAAGGTGTCCAAGGTCATGTTATTGGAATTGATCTTGGCACAACCAATTCTTGTGTGGCTGTTATGGAAGGAAAACAACCCAGAGTAATTGAAAACTCTGAAGGTTCAAGGACTACACCTTCAGTTGTAGCTTTCACTAAAGATGGAGAAAGATTAGCTGGTACTCCTGCTAAAAGACAAGCTGTAACTAATACCCAAAATACTTTTTATGCTACTAAAAGGCTAATTGGTCGCCGTTATGATGACCCTGAAATTCAAAAGGACTTAAAAAACTTGACATTTAAAATTGTAAAAGCTACTAATGGAGATGCTTGGGTGCAAGGTACAGATGGAAAAATGTACTCCCCTAGTCAAATTGGAGCATTTGTTTTAATTAAAATGAAAGAAACTGCCGATTCTTTCTTAGGAACAAATGTAAAAAATGCTGTAATTACTGTTCCAGCATATTTCAACGATTCTCAAAGACAAGCTACAAAGGACGCTGGACAAATCGCAGGACTCAATGTCTTACGTGTCATTAATGAACCTACTGCTGCTGCATTAGCATATGGCATGGAAAAAGATAGTGATAAACTTATTGCTGTTTATGATTTAGGTGGTGGTACTTTTGATGTTTCTATTTTGGAAATTCAAAAAGGTGTGTTTGAGGTCAAATCGACCAATGGAGATACCTTACTTGGTGGAGAAGATTTTGATAATTTATTGGTTAATTATTTAATATCTGAATTCAAAAAAGAACAAGGTGTTGATCTAAATAAAGATGCTATGGCTTTGCAGCGTGTAAAAGAGGCTGCCGAAAAGGCAAAAGTTGAGTTATCCTCTTCTTTACAAACTGACATCAATTTACCATACATAACTGTTGATAGCTCTGGCCCAAAGCATTTAAACTTGAAATTAACTAGAGCTAAATTTGAAGGACTAGTCGGTGATTTGATTAAAAGAACGACAGGACCATGTCAAAAAGCTGTTAAGGATGCTGAAATAAAACTTTCTGATATTTCTGATGTATTGTTGGTTGGTGGAATGACTCGAATGCCTAAAGTGCAGTCATTGGTACAAGAAATATTTGGAAAACAGCCTAGTAAAGCAGTTAATCCAGATGAAGCTGTTGCTGTTGGAGCTGCAATTCAAGGTGGAGTACTGTCTGGAAGTGTTACAGATGTGCTTTTGCTTGATGTTACTCCATTATCTCTTGGTATTGAAACAATGGGAGGAGTATTTACTAGCCTCATCTCTCGTAATACAACCATTCCAACTAAGAAAAGTCAAGTTTTCTCAACAGCTGCTGACTCTCAGACTCAAGTAGAAATTAAAGTGTTCCAAGGAGAGAGAGCAATTGCTGCTGATAATAAACCCCTCGGCCAGTTTTCATTGGTCGGAATTCCACCTGCACCTAGAGGTGTTCCACAAATAGAAGTAACATTTGACATTGATGCTAATGGTATAGTTCATGTTTCTGCTCGTGATAAGGGTACTGGTCGTGAACAACAAATATCAATCCAATCATCTGGCGGTCTTTCAAAAGACGAGATAGAAAATATGGTGAAGAATGCTGAACAATATGCCCAACAAGACCAAATTAAAAAAGACCGTGTTGAAGCACTTAATCAAGCTGATTCAATTGTTAATGATACTGAAAGCAAATTAACAGAATATCAAGTCCATATACCTGAAGAAGATGCCACCAATATCCGAGAACTAATAAAAGAAGTTAGAGAAAAAAATTGCAGAAGCTCAAACAAATGAAACGGGGGATCCTGAAGAATTAAAATCAAAAACTCAAAAACTGCAGCAAGCATCCTTAAAAATGTTCGAAGTAGCTTATAAAAAGATGGCTGCAAAACGCCAAGAAAGTGAAGGTCAGACAGCTGATGAAAAAACTAAAGAAGAACAGTAAAATAATATCTAGTATGTAAATAATATTGTATAATATAATGTAACTTATGAATGTAGAACTGTAATAAATTGTCTTATTATCTTACCTCTGATTAAAATTGTTTTAATACTATTTTGAAGAATGTAAGTGCTAAAAAAAAGGCCAGACATACTAGTAGTATATTATTTAGATAGAAATTGAGATTTATATTCATTTTTTGCAATTTTAAATGTTATGTAAATAAAGAAAAATTGAACACAAAAAAAAAAGG

>CL566.Contig2

TATATATATATATATATATAAATAATATTGTATGGTTTTTTTTTAAACATTGGAAACAAAAAATCATAATGACTTATTGAAGGGAATTAAAAACAACATTATTTTTTTCAAAAATTAATTAATTAAAAATTAAGATATTAAAATTAAAATTAAAACGTTTATGCATTCTAAAAGTGGTTCACTAGATAGAAAAACAATTAATGATGCGAGATTGGAATCAATTTATCAGGCCCTCCTAAAGCAGGCAACGGTGCTTCAACAGTTAACACGCCGTCTTTGCTAAGAGATGATTTGATCGCTTCCGGATTGGTACCTTTTGGAAGTAAGAATTCCCGGTTGTATTCCCTGTACACCGATTTTGAATCTGATTTTTCTTCATGCTTGGCATGTACCAATAGTTTGTTGTCGACTGTTTTGACGACGATTTCCTCGGGTTCGTATTGACTGACGTCGAATCTGAGTTTCAACATTTTGTTGTCGCCATCCTCTTGAATCAACGGCGAATTGATGCTTTCAACCCAACCGCTAGGCGCAGCCCCAGTTAGTCGGCTAGCTGGCAAATCGCTGTGGTTCGACGAGATTGTGTTCTGAGAGAAGGACTTTTCTTCCACGTGTGATGTCTTGGACGTGGTCTTGAAGAAGTTCGACTCTTTGTTCATGAGTTCGGAACGAAACTTGGTCATCTCGTCTTCCATTTTTCGCATTTCAGCGTCAAAACGCTCGCGGATGTTGCTGAATTCTGAGTCGATCACGCTGAAGTCGCCCAACTTGATAGGGATGTCTCGCTTGACGCCAGTGTTGTGTGCCATTTATATTTTAGCGTGTGCAAAGAAAATAATCGATAACGGAACGGACGAGAGTTTGAAGGGGTAAAAATGCGACGGTAATAATATAAGATATTATATAATAATATCTAATATGATATAATAGGTAGGTGGTTTGTATGGCGAATTATATTCCGCTAAAAATCGACTGTTGTATGGACACGC

>CL5740.Contig1

CCAACTTTTAATGATGTAAAGAATCAGAATATAGGTTATAATTAGTAAAAAATAAATAGAATGCGCAATTTTGTATATTGTAAAACAAGTTAAACAGCTACAAATATAGCAGATGATTTTAATTCGGTGTCCATATTTTTTTGCTTCAACGTGCAATTGATCTACGCTAGCAGTTAATGTAAAAGTAATTTCTCCTTCTGTTTTCAAATTTATATGTGCTTCAGATTTATTACTCTTATTATTTCCTGATGTAGCTGTAAGAGTAATATTATCAACATTAACACTAGTTGTTATACTTTTTGTACTAGGTAATATTTCATTTTGATCAAAACTTAAAGTTTTCCCACAACAATCAATTTCTATTAATTCATTTAATGTGTGAACTTCTGTGGTAGGTTCTGGTAAATCATCATGTGTATTCCAATATTTTGCTAAGAAACTAGCTTGTACAGCCGCCCCGTAAGCCAATACTTCGTCAGGACTAATACCATTTGGTATATCTGCATGCGGGAATAAGTTTCCAATATAATGTTTCAACTTTGGTATTTTGAGTGGACCACCACAAAGTAAAACTTTTTTAATATCTGAAGTTTTAAGATTAGCTTTCTTTAAAACAACTTCAATAGGCAAAGCAAAATCAGTCAACAAATGAGCAATTAATGATTCAAATCTTGCTTGTGAAACTGAGGCAGCAAAATCAATTCCATCAGTCAATGATTCAATAAATATATTTGCTGTTTTTAATGTAGACAATAAATGAACTGCATCTTCAGTAGCTCTTTGTAACTTTGCAGAAGCTCTTGCATTTTCTGAGGGATTTGATCGATGTTTCCTTTTAACTTCTTCAATTAAATGAGCTTTCAGTGTTTCTATTAATTTATATCCTCCAATAGCCTTAGTATGTTCAGATGCTACAATTGACACTAAACCAGAATTTACGTATAAAACTGTTGCATCACAACTAATACCTCCAAGTCTATATACTAAAATATACGTATCATTTAAAGGTTCTGTAACATTAATTCCATAAGTAAAAGGAGTTACACTAGGAGCATTCACGACTTGAAGCACGTTCCATCCAGCTTGAATCGCTGCATTTCTAACTGATTCTCTACTTGGATCATCAAAAAATTCTGGTACAGTTACAACACAAGAACATTTTGTATCTTCACTGTGAGTGGCTCTTTTAGCAATACCATACAATGTTTTATAAATATTTGATTCAATGAATTGAGGAGAATAAATTTTCTTTTTAGTGTCCAAGGGTAATTCGTATGCAAGATGACCTCCGTCTTCAATAGTTTTGACTTTATTATTTTCTATGAGTGAACGTTTCTCATTTTCGTCCAAAGAAAGGTTCATTAAACGATTATTAAAAGTTATTGTATTAGCTTGTTTTGATATAAAACCAGTTTTTGCAGCATTTCCAACAACAGTTTCTTCATCAGTTATAGATACAATTGCAGGTGTTATTCTGTCTCCAGCTTCGTTTGCAAGAACATCCACCTTACCTTCTTCTTTGGAGATGGCTATTGATGCGGTTGAGTTTCCGACGTGTATTCCAAATACAATTGAACGTGACATTCCTAAAAAAGTAGTCCAACAAATACAATAACTAAAAAAAATCCAAAATTACATAAAAACAAAAAAAAACTA

>CL5767.Contig1

GACATAGTGTAAGTATAATGTGAAAATATATAAAATAAAGTAATTTATTAATTAATATTAAACTCCAAAAGATTTTTCGAGATTTTTTAAAGCACTTTCTGAAACAAATTTTGAAATGTAGTTCAGCAATTCTTTGATACATGATACTTCATTACTAGAACAAAACATAACTAACATTTGTAGTCTTGGTAATTCACTAATGCCCATCAAATAATCTGTTATATCACATCCATTTTTAATGAATTCTATCCGTAAAATTCTTAAAATATCCACAAATAGATCATTATCCATAGAATTACGTACAATTGATGGTAAACGTTTACCAGGAATTTGTTTTAAATATTTAAATAATAACTCTGGATCATTTTTTAAGCTTGCCCAATCTTGTTTAAGTTGGACGTATGTTAGTGGAACAGGAGGTATGGCTATGTGTTTTTTTTCATTTTGAATTAAGTCTTGTTCCATAGGAACAACTTCATTGGTAATAGTCGTTTTAGATTGATCTTTATAGTTTTTTTTCACACACTTCTTTCAATGTAGTTTGAAGTAAATGCATGTTGTTTAATTTTTTTTTTGATCTACTTGAACATCTTTGATGTTGAGTGTACATAAAGGTTTTTTAGATCTTAAATGTGGTGGTTTTTGTTTAGAAGTTATTTGTTTACAACCATCTCTTTGAGGCCAATCTGGAGTGACACTTTTTATTGGTTCAACGTCTTTACATAACATTTTAGAACTTTTATTTTGTAAGTTATTAATTGTGTTTGTTTTTTGACATTTATTATCAGAAGTGTTCTGCATTCCACTCTGTTTGATTTTATTGTTTACAATTTCTATATCATTTTTAGCTTGTTTGTTATTTGGTTCTAATTTTAACACATTTAGTAAATCTTTTTTTGCTTCATTAAACATTCCAAGAGCCAAATAAGCTGAGCTTCTGCGTTGGTATGCTTTAACATATGTGTTATCTAGTTTAAGAGAAGATGTACAATCAGTTATTGCTAGTTTAAATTCTTTTGTCTTTAGATAGCACAATGCTCTATTGGCATAAAATATTGGATCTTTATCATAATACTCTATTGCTCTTGTGTACCTTAGTATAGCCTCAGCCCATTTTTGCTTTTGAACTAATGCATTACCCAAATTTTTTTCGTATATACCATCAGATTGTCTTGATTGTGTTTCATTATTTAAACAATCGACACTTGGATCATAATTATCATTTTTCTTGAGTTCATCTTGAGTATCTAGTTCTTTGCAAGCATTATCAACATCAAATTTGTCCCAAGCAGAGTAATCCCATGATGAAATTTTGTTTAATTTAGTTTTTGGTGGATTAGTTTTTGATGGATTAATTTTAGGTTTTTTTCTTTTCATTGGGATTCGGTAATGTATGTTCTTTTCGCTTCATTTGTTCTTGCCAAATGTTCATATCACGTAAATAATTTGATAACTCTTCTTCATTCTCTTGTAACTGTTTCTTAAGTAAATATGGATTATTCATGCCAAAACGTTGAATTGAACACAAATCTCAGAAATTTATATCCAAGTGAAAAGTCGTACTGACGGTTATACAATAAATAATACACCACAGGTAATAGACACCATGTATATCCGTCTATGGCAGCAGACCGGACAAGACAACCAAAGGAGTAAGGAACAACACTATGGACGTTAAAAAAAGATATTCATCAATGATGACTGATGTGCGTCGAGTACTGGAGTAGTAAGTTGTCCAGTGATGACCATCGC

>CL5943.Contig1

TGATTTTTTTTTTTTTTTTCTTTTTTGCTAATTTTTTTAGAGCAATGATCAAGCAATCATTTTATCATCAGTGAGATTCATATATTAAACGGTCAATTAATTTACCTTGAACTATATTAAGTATCTTTTTATTTTTTATTTTTCAATTTTTTTATTCGCAACAGACCATTAAACGCCAAGACACCAAACTCACAATCATCATCATCATATTATAGAAAAAAAAAAAGAGAAGAAATTTAGCAATTTAAAATGAATTTTTCGAGCACTATTTGATATTTTTTTTTGGATAATTTATGACATTGAATTTCGCATGTCACTCATAAATTTAGCACCTAATTTATCAGACGTTTTCAATTGTGCTCTAGCAGCATCACGAAGACTAAATATATGAGTTAATTCAATATTAGTACGTGAAAGTGGAATTGCTTTATCAAATATTTTAATGGCATTTAATAAATTTCCTCTTTGTACTTCAACTGTTCCCAATGTTTCATATGCAAATTCACATTTATCATCAATTTCAATAGCTTTATTTATAAATTCAAGACCTTTTTCAATATTAGCTTGCCATTGTAATTGTAATAAACCTCTGTGTACATATAGTGTTGCATTTGTTGGATCAACTTTTGATGCTTTTTCAAAATATTCATCAGCTTTACCAAATGCTTGTGAATCACATAACAATTGTGCATACAATGAAAAACATTCACAACAATTTGGATATAATTCAAATCCTTTTTCAAAATCATCAATAACATTTGCAAGCATTTCTGATGAACGATTAATAGCACCAAAACGATAATTAACATAACATTTTTGTGCATATGCTACACCATTATTTGGATCTAATTCAAATGCTTTATCTGAATCTTTTTTTTGCTTCATTTATTTTTTCGAATAATAAAAGTATCTGTCCACGATGATGATAAATATCAGAACAATTTGGATTTATTTTAACAGCTGTATCAAAATCTTTTAAACATTCATCTCGTTTTTCTTGTTGCATACTCATCAAAGCTCTTTTAACTAATGCATTAACACGTACATCATCACCAGATATTTGATAATTATCAATTACTTTTTCAAGATCATCTAATGCTTTTTTATGATCACCAACAAGAATATAAAATGTTGCACGATGAAGCATTAATTTCATTTTATTTTGTGAATTATCATCAGTATATTTTTCAATTTCTTCAGTACATGCATCAATAACTTCATCATATAATTCGTCTTTCATTAGCTTGATGATTCTCACAAGACATTTGTTTTCTTCTTTTAATAATTCTTCATCATCAAGTGATAATATTGGATCATTTTTAAATGAACGAAAATATGTTTTAATATGATATGTACTTGGTATTGTTATTTTTTTATTTTTTAAATATTCATCAGCATGTTGTTGACCAAGTTGTTTCAATACACGATCAGCAACAACAAGTGATGCTGTATTTTGAAAATGACCAACAATACATGCAGCAGTTGCATCTTCAAGTGCAAGTTCAAGTTCATTTTCAAGTTCGTATGCTTTAGCACGTCTTAAAAATGCTTTTGCATATTTTGGATTGTATTCTAATGCTTTTGTACAATCATCTCTAACTGCACTATGTTTACCAAGTGCTTCATAAGCAGCAGCTCTATTTTGATAATTTTTAGCTAATTCATCTTTATTTTCAATTGGACAAGCTTCAATTGCCTGATTATAAAATGATATTGCTTCGTCATATTTTTTTGCACTATATGCTTCATTACCAAGTGTTTTAAATTTCAATGCTTTTTCTGCTGGTGTCTCGGTTTTTTCGATTGATGTTTTTGATGATTTTTCTGGTAAATCATCATCAATTGATAATTGTTTATCACTACGTTTTTTATCAATATCTTTAGTTTTTTTGTCTTG

>CL6327.Contig1

GTTTATTTTAAATATAAATAATAAAAAACATTAGACATAAATTATATTTAAAATGAATATAGAGCTATTTAAAGCATAATCAGAAGTTAAATGGTGGAATTTTTTTAAATTTTAAACAAATTTGAACGTAACTATTTCCCACTAAATTAATCAGTCGCATTATAATTGTGTAACACATAATTTATTTATAATTACTCTACTTTTGCTAATAATTCTGATTCTTTGAAAATTGTGTATGAATCATCATCGTCCAATTGAATTTTAGTACCACCATATTCAGGTAACAACACTCGATCGCCGACTTTAACATCAATTGGTATTGGTTTTCCATCCTGATTTCTTGCACCAGGTCCTACAGCTACAACAGTTGCCTCAAGAATTTTTTTCGAAGCACTCTCTGGCAACATTATACCTCCTTTACTTTGTTTAACAGCGTCCAAACGTTTGACCAAAACTCGATCAAAAAGTGGGCGGAATTTGGTTGCGATTGATGCCATTTCGATGAGGTAAAGCTTCTGGTAGTTCAAACAGTGAAATGTAATATAAAATTACGATCGAGGAAAAACTGGAACGAAAAGATGTGAGAACGAAAAATGACTAATGAGAATATTAAATTTATTGAATAGGTTAAAGTAAA

>CL7120.Contig1

GTAATAATCTACTAAACACAGTGTATCATGGAGATAATTTTACATTAAAATTACTTTTAAATGTGTTAGATAATTATGTTACACTCAGGGCCTCTGTATACTCCATGATAGTAGGGACAACAATTGATCATTGTCATAACACTACTGAGGTTTGTTCTTAATATAAATACAGTATAGTTTTTTAAATTTTATAAATATTTATTAGTTAAAAATATAATGATATTGAAGAGGTATGTAATCTAATTCAACAGTTCATTCTTCTTCAAAATCAGCTTTTTTTATAAGTTTTAAACTTTCTGCTAGTTTCAAACATTTATTGGCCATTTCTCTAATCTCTTTTTTTCTTCTCGTCTTCCATTATTGAAAGTGCCATTAATACTTCCATTATGTCAGTGGCAAAAATTTTTTCAGCAATTTCTTTGTCTGTACCTATTAAACTATGTGCAATCACCAAAGCTCTATGCTGCATTGCATTATTAGGATGTGCTAATAAAGCCTTCAAAGATTCTTCCCAATTGTTAAGCTTCATTAATTTTTGACAACATTTTGTACTATTTGACACAGCAATTGCAACTGCACCAGCAGCAGCTAATGATGTATCTTCATCCTCTTCTTCAGATAATAGAAACAACATTTTTAATTTGTCATTTTCACCCTCAATTATCTTTATGACGTCTTGAGACATCATAAGATTAGTAAAACATTGGGCAGATGCTCTCCTAAGCATTTGGTGTTCTTCAAATAAATAACTCTCAATAAGCGGTAGACCACCAGTATCTAATATTTTTTTTTCGTACTCTTTCATTCACAGACGCCATGTTACAAAGTGCCATCATTGCTTCAAAGTTTTCTAATGCTGTACAATCCGGATGCAATAAACTTAGGAATGGTCGAACAGATTCTAGGCAACGTTGTTCTTTGAACGAGACTTCTGGATTGATGGAAATTCCTATTCTAGCTAGAGCTTGAGAAGCTTGTCTTTTGCCTTTGGGTGTACCTGAATGAGCAAGTTCCAAAAGAGATTTTATACCACCTAATTTAGCGACTTCACCTCTGATTTCTGGTAGGACACAAATAGCGTTGAACAACCTTGAAATAAGTTCTTTGATGTTTAAACTTTCTGTTTTGGATAGTTGTACAAGGACTGTGATTACACCATGTTTACATAACAATGTAATTCTTTTATTGACAAAATCAACATCATCTAATTCATGGTCTTCGGGTACATGACATTTAGAGAATTTTGCAAGTTCAATCATTTCTGGAATAATCTCTTGTTTTTCATATGCATTACACAAATTAACAAATATTGATACTAGAGCATACATTGCTGATGTTTTCTCTGATTTAGCTAATCCCATAATGGCTACCAATGCTGCTTGATCTTCAACTAGCTTTTCTTTAACCTCTGCATCTAATGTTAAATAAGATAACCCTTCTACAGCCCATCTTTTAGTATCTGGATTGGTATCTGGATGTATTAAAAATCTACGGCAAGCTTCAGCTAATTTAAGAGTAGAGCCTTCTGAGAATGGACGGATAGAAGCATCACTTCCACCACTACTACCAAGTTTACATAAGCCAACCAATGCACGAATACGTATGTCGTCATCACCACTCTTGTACAAATTTTTAAGTATTTGAGTACCTTGTTTTACAATTGGTGTAATCTTACTTTTCTTTGATGTAGCAGCAATAATACACTCACATGCAATTTTTTTGCTGTACTTTATCATTTGTTGTTGCCATTGTAAGAATCATTTCAATTATGCCATCTCTCGATATAATTGAATTTCCAACATCTATTGGGCCCATAAGTAATACGGTTATAGTTGATATAGCTCGGACTTTAGATTCTATATCTGGATCTAATAGCATTCTCTTCAGGAACTCGTCAACCGCACTCACATACTTCTCTCTAGATTTATCACAGGTCATGTTGTCATAAATTTTTGATAAGCAGACTGAAACGTTAGTATGCGAATATTCAGTAATTTCCATTTTGGATTCATATCTGATTTCAGTTAGTTCAGCAGCTACTTCCAACAATCTCTGTAATCCTTTCATATCAATTAAACGTTCAGCCCAATTTATATTGTTAAAATGAACATTTCTTGTTAATATTTCTATAATGGAATCTCTTGCAAACTTATTTATAGAATGTCGGTCCACGGAACAGACTAAGCAAGTTAGAATAGCATCTATTTCTTTAGAGTTCTTTGCACATTTTTCTTCATTTGGTGAACTATCCAATGTGTTAGACATGCCTGTTAAACTATTTAATGCTTCTTGTATAATAAATGTAACACCATTTATTTCATTTTGATTTTCAGTATTTAACGCGTCTATAAACCAAGGAACACCAAGTTCAACTAAAACTTGCTTGGTACGAGTCTCATCATTTTTGCACAGTTGGCCAACTATTCTTAAGGCGTTTATTCGTACTTCAGTATTTTGTTCATTCTTTAAGAGACTTTTAATTTTGTTTACAATACCAAATTTTAACAAACCAACACTTCCACTACTTGATTCTTTAGATAAAACCAATAAATTTTTAATAGCTGTAGTACGCTTTTCTAAATTCTCTTCAATTTTAAAAGCATATTTGAACATGCTTTCAATTTTATTAGATAATTGAGCATTTTCTCTCACTTTCTCTTGTACAATTGGATGTAATCTTGACAAAACAGGCTGTATTTCTTTATTAGTGGGATCAGACGACAAACATTGCTTACCATCTATGTAGGCTTCTTCATATTTTCCAAGCTGTTCGTAAGCACAACAACGACGGAATAATGCTTTAGGATCATTTGCTACTATGTCCAATGATGCACTACAATCACGGATTGCATTTTCAAACTCACCCAACTTGTTGTACACAGCAGCACGGTTTTTGTACAAAATTGATTTATCCCGAGTGTTTTCTTTCAGCAGATCCAACGCAGAAGTGTAACATTGCAAAGCTAATAACCAGTTCCCACTTTTAAATGCTGTATTGCCTTCTTCTTTCAAAAGATTTGAATCTTTAGCAACATCTCCCGACATTTTAAATTATAGACTTTAAACGTACTGATTAAACACGATTTGTTCAGGTAATAAGGTAAAAATCAAATGCATGAGCTTTTTATAACTTATTAAATACTTGGAAGTACGCCAAGAAAGAAACCTTTTATAGAACACTAGAAGATTCTGG

>CL7882.Contig1

GAAGACTTCGACAACCGGCTGGTGTCTCATTTGGCCGAGGAGTTCAAGCGGAAATTCAAGAAGAACGTGCACGCCAACCCGAGATCGTTGAGAAGGCTTAGGACGGCCGCCGAACGGGCCAAGAGAACGCTGTCGTCCAGCTCCGAGGCGGTCATCGAGATCGACGCCCTGATGGACGGCATCGATTTCTACACACGAGTTTCACGAGCCCGTTTCGAGGAATTGTGCGCGGATCTGTTCAGATCGACTCTGCAACCGGTGGAAAAGGCGTTGGCGGACGCCAAGTTGGACAAGGGAGACATACACGACGTGGTGCTCGTGGGTGGTTCGACGAGGATCCCGAAGATTCAGAGTCTGCTGCAAAACTTTTTCTGCGGCAAACCGCTGAACCTGTCCATCAACCCCGACGAAGCGGTAGCCTACGGCGCCGCAGTGCAGGCGGCCATTCTCGGCGGTGACACGAGTTCTGCGATTCAAGACGTGTTGCTCGTGGACGTCACTCCTCTGTCGCTGGGCATCGAGACCGCAGGCGGCGTGATGACCAAAATTGTCGAGCGCAATTCCACTATTCCGTGCAAACAAACCCAAACGTTCACGACGTACGCAGACAACCAACCGGCCGTCACCATCCAGGTGTTCGAAGGTGAAAGGGCCATGACTAAGGACAACAATCTGTTGGGAACGTTTGACCTGACCGGTATACCTCCGGCGCCTCGGGGCGTACCCAAGATCGAGGTGACTTTTGACATGGACGCCAACGGCATTTTGAACGTGTCGGCCAAGGATAATAGCTCCGGCCGCTCCAAGAATATCGTCATCAAGAACGACAAGGGTCGCCTGTCTCAAGCCGAAATCGATCGTATGCTCAGCGAGGCCGAACGGTACAAAGAAGAGGACGAACGACAAAAGGCCAAGATCGCGGCAAAGAACCAGCTGGAGAGCTACGTGTTCAGTGTTAAACAAGCGTTGGAAGAGGCCGGCGACAAGTTGACCGAATCCGAGAAGAACACCGGCAAACAGGAATGCGACGCGGTCGTCCAGTGGTTGGACAACAATCAGTTGGCCGACAAGGAAGAGTACGAGTACAAATTAAAGGAAGTCCAGAAGAGTTGCTCTGCTCTTATGATGAAGATACACGGCGCGGGACAACCCGGGGGCGCGCCTCCCGGTGCGCAAGGTTTCCCTGGCTCTAGAGGACCTACCGTCGAAGAAGTCGATTAATTGTTTTATAAAAAGTAATCTCATGAAACATGTAAATACTATTGTTAACTACTTACATTTTTATTTATTTATTATTATGTTGCGCATTACCTTGCCGTAATAGTTGCAAAATAAGTATTTCTGTGTTAATGTTTCTCATACGCATATGGACATATTTTCTGTTAGCGTAAGAGAAAAATAAAACATTTCTTTTAGTTATGAATGTTGTTTATTAAGCGATGGAGCCCCAAGGTTCGATATATATATAAAAAAAAATTTTTTTGACGAGACTGAGCTAGTATTCAACCAAATTATGTAAATTAAATTTACTATATTTTTAATTAGTTATGTTAGGCAAAAAATTTTTCAACCTGTGACATCCTCTTTTTTTTTAGGTTGAAAAATTGTATACTAATAGTAAAGTGCTAATGGTTATACTTTTAACATGTAAAATCAACCGTGGCATTGTATTAGCTGATAAAATAATTTATTCTGTATAATGTAATTATTTTTAGCACTAGCTGAAATAAATAAAGATGTATTTGTTGACTGAAGCCAAACAAAAAAA

>CL7882.Contig2

GATGTTCTTGGAGCGCCCAGAGCTATTGTCCTTGGCCGAAACGTTCAAAATGCCGTTGGCGTCCATATCGAAAGTCACTTCGATCTTGGGCACTCCTCTGGGTGCTGGAGGTATGCCGGTCAAGTCGAACGTTCCCAAGAGATTGTTGTCCTTTGTCAAAGCCCTTTCACCTTCAAACACCTGCACGGTGACGGCCGGTTGGTTGTCCGCGTACGTGGTGAACGTTTGAGTTTGTTTGCATGGAATGGTAGAGTTGCGTTCGACGATCTTGGTCATCACACCGCCCGCGGTCTCGATTCCGAGCGACAGGGGGGTGACGTCTACGAGTAACACATCTTGAATTGCAGAACTCGTATCGCCGCTCAGTATTGCCGCTTGGACTGCGGCGCCGTAGGCTACAGCTTCATCGGGATTTATGGACAGGTTGAGCGGTTTGCCACAGAAAAAGTTTTGTAGGAGACTCTGAATCTTCGGGATCCTCGTCGAACCACCCACGAGCACCACGTCGTGTATGTCTCCCTTGTCCAACTTGGCGTCCGCCAACGCCTTTTCCACCGGTTGCAGAGTCGATCTGAACAGATCCGCGCACAATTCCTCGAAACGGGCTCGTGAAACTCGTGTGTAGAAATCGATACCTTCCATCAGAGCGTCAATCTCTATGGTGGCCTCCGAGCTGGACGACAACGTTCTCTTGGCCCGTTCGGCTGCAGTCCTCAATCGTCTCAACGCTCTCGGATTGGAATGTACGTCTTTTTTGCATTTCCTCTTGAACTCGTCGGCCAGATGAGCCACCAGCCGGTTGTCGAAGTCTTCGCCGCCCAAGTGCGTGTCGCCCGCCGTCGACTTCACTTCGAATATCGAACCCTCGTCGATCTGCAGAACGGACACGTCGAACGTGCCGCCGCCCAGGTCGAATATCAGCACGTTCTTCTCGCCCTTCAGGTTCTTGTCGAGACCGTAAGCCAGAGCTGCGGCCGTGGGTTCGTTGATTATCCGCAGCACGTTGAGTCCGGCGATGGCTCCAGCGTCTTTGGTGGCTTGTCTTTGGGAATCGTTGAAGTACGCCGGTACAGTGACCACGGCGTCGGTGACCTTTCCGCCAAGGTAGACTTCGGCGATTTCTTTCATTTTCGTTAGCACCATCGAACTAACTTCTTCA

>CL7882.Contig8

CAACGATCAAGGTAACAGGACCACTCCAAGTTATGTGGCGTTTACGGACACCGAACGGTTAATCGGTGATGGAGCCAAAAACCAGGTGGCGATGAATCCCGTCAATACGGTATTCGATGCCAAACGTTTGATCGGCCGTCGTTATGACGATGATAAGACACAAACGGACATTAAGCATTGGCCGTTCAAAGTGATTAACGACGGTGGAAAGCCCAAGATCCAAGTGGAATTCAAAGGTGAGCGTAAAGTGTTCGCGCCGGAAGAGATCAGTTCGATGGTTTTGACGAAAATGAAGGAAATCTCGGAAGCTTACTTAGGCCATAAAGTGACGGACGCTGTCATCACGGTGCCGGCGTACTTCAACGATTCGCAGAGACAAGCGACAAAGGACGCGGGTGCCATAGCCGGGTTGAACGTAATGCGGATTATCAACGAACCGACAGCCGCAGCTCTGGCATACGGTTTAGATAAGAACCTGAAAGGCGAAAGGAACGTGTTGATCTTCGATCTGGGTGGCGGCACCTTCGACGTTTCAGTCTTACAGATTGACGAAGGTTCGATATTCGAAGTTAAGTCCACGGCTGGCGACACACACTTGGGCGGTGAAGACTTCGACAACCGCCTGGTGTCACATTTGGCCGACGAGTTCAAGAGGAAAACCAAAAAGGATGTGCGTGCCAATCCGAAAGCTTTAAGACGGTTGAGGACGGCTGCTGAACGGGCCAAGAGAACTTTGTCGTCCAGCTCAGAGGCGACCTTGGAGATTGACGCTTTGGTAGACGGTATCGATTTCTATACACGAGTGTCCCGGGCACGTTTCGAGGAGCTGTGTGCCGATCTATTCCGGTCGACCTTACAACCAGTGGAGAAAGCATTGGCGGATGCCAAGTTAGACAAAGGAGACATAAATGATGTGGTACTCGTGGGTGGTTCGACGAGAATTCCGAAGATTCAGAATCTCTTGCAAAACTTCTTCTGTGGTAAGCCACTCAACCTGTCCATCAACCCCGACGAGGCAGTAGCATATGGCGCTGCGGTGCAGGCGGCTATCCTTAGCGGTGACAAGAGTTCTGCAATTCAAGATGTATTGCTGGTGGACGTCACACCGTTGTCGCTAGGCATCGAGACCGCTGGCGGTGTTATGACCAAAATCATCGAACGCAACTCTACCATTCCATGCAAACAAACCCAAACTTTCACCACTTACGCGGATAACCAACCGGCCGTCACCATCCAGGTGTTCGAAGGAGAAAGGGCTTTGACAAAGGACAACAATCTGCTAGGAACGTTTGATCTAACAGGTATACCTCCAGCGCCCAGAGGAGTACCCAAAATCGAGGTGACTTTCGATATGGATGCCAATGGAATTTTGAACGTTTCGGCCAAAGACAATAGCTCCGGGCGCTCAAAGAATATTGTCATAAAAAATGACAAGGGCCGTTTGTCTCAAGCCGAAATCGATCGTATGCTCAGCGAGGCCGAAAAGTATAAAGAAGAGGACGAACGACAGAAAGCAAAAATCGCTGCCAGAAACCAATTGGAGAGTTATGTATTTGGAGTTAAGCAGGCATTGGATGAGGCTGGCGACAAGTTGACCGAATCTGAGAAAAATACGGGCAAAAAGGAATGCGACGCGGTCATCGATTGGTTGGATAGAAATCAGTTGGCGGAAAAAGACGAATACGAGGACAAGTTAAAAGAAATCCAAAACAGTTGCTCGACTTTAATGTTGAAGATACATGGCGCAGGACAAGGTGGAGCAGCTAACATGCCTCCTGGTGCTAATGGTTTTACTGGAGCAAGAGGACCAACCGTTGAAGAAGTCGATTAATTCTGCTACAATGTAATCGGATATTCGTAAATACTATTTTGAACTTAATCCTATATTTTGTTTCCTACTATTTATAGAATTCACTATAGATTTAAGTTAAGTAGATATAAGTTTACATTTATCTTTCTATTGTATTTCATAATATTATGTTTTCGGCACA

>CL7897.Contig2

GTGTGCTCTGTTAGCTTTCCGTGCCGACCCGCGTGCATAACATTTGGTTTTTATTCGTTTTTACGATTATTTCCGTCCGCAAGTCCGTGTGACCAGGTTTTCTGTCGTCCGCAACAAGCACACGCACTCCGCACCGGTGCGTTATACCCGTTGATTTTTCAAACGTCGCGAATCCCCGTCCAGCTGTACGTTTTTTAATTTTTTTTTTAAATTTTTTTAATTTTCAAAACCCCAAAACCAAAATGAGTACAAAAGAAGCCAAGAATGACGTCGCCGTAGACAAGATCGCCGAAAACGATGTCGTAGACGCCAAGGCCGACCTTAAGGGAACCAAAAGAACAGCAGACGAAAAATCAGAAGAAGTTAAGAAATTGAAAAAAGAAGAAAATGGTGAGGAAGACCTAGATGAAGAAGACGAACCCGAAGGCGAAGAAGATGAGGAAGAAGAATTCGCCGAAGGTGAAGATGATCTAGATGAAGCTGACGGTTGTTCAGAGGATGAAGATGATGATGTAGATGGTGTAGAAGAAGAAGATGATGTGTAAACTGACATAATGTACAATTGAACAAGTCTCTTAATTTTATATAATTTATACATAGTGCCGCATGTACTCGGTAACTCCGTTCGATAGAATTTGATTCCAAAATGTAATTTTTTTTTTATACATAATTTGTAAAACAAAGCTTTTTTGCTACTGAGTGACTAAGACAAAATTGAATTTTTTTTTAAAATTTTAAACACGATATTCCTACTAATTTGTCTTGTGTACATGTTCTAAGTCTCTAGTCACTCACTATCAGTCAAGTTTTGAGCATGTACTTGTGAAAAAAAAGTTTCTAAACTTTAGGTAACACTTGACTGGAATACTTAAAACTAACTCGACCACCGACTATTACACAATGGTTTTTTTTTTTTTATAACAGATTATAAATGCAATAATTTCCTTTTTTTTTAAATAAATTTAATACTACTTAAGTATAAGTAAATAATATAAATAATATTTAAAAATAAAAAATTGAAATACTGAATTGTAATTTAGATAATAGGTTTGTCTTCTAAAAAATATATATAAATATATATATATTATCGTTTAAAATTTAAGCTACTTTTGTATAAACATTTTGATTAAATGATAAGATAATCAAATAAGTACCTCGTGTTGGTTTTTTTTATTTTGTATTCTCGGTGTGTTTCAAAGTTTAGGAAATTTTTTTTTTTGTTTCTCATTCCAACTAAAGAATTTTTTTAAGAACTTTTTAGCCACTGAAAATATGTTTTAAGTTAACGTTTAACATTAAATTTAAAAAAGAAATTGTAATGTCAATTTATTTTACCCCATTTAAGTAAAATAAGTATATATAATATGATATAATTTAATATAAATATATATATATATATCGCCAAGTCTTATCTAAGTAATACAATTTTAATGATAATGTAAGAAAACTAATTAAGAGCAAAAGTTAAACTGTATTTTTGTTCTTTTTTTACGCTGGCATTTTTATTATTTTTTATTTATACTTTCAAGTAAACGGACAGGATTCATAAATATCTTTAAAAGAAAAAAATCAAAAATTATTTCAGTTATATATATTATACAATTGTTTTTTAAATGCTGTGTGGCATTTTTTTTTTTTTTTGACATATTGGAGGAAACATTTTACATCTGTATTCCTAACAAATAATTTTATAGAATAATTAAAAATATTTACATTCTAATCTAACGTCATAAAAAAAATATATATATATACGTGTAGTTTTTAAACATGATTGTAATCATTTTTCCTGTTGTTTATAAGTTAAGTAAAAAAAAAAAAAATGAAAATTAAATCCAAATCACCTGGCTCACATTTGTGCTGAGAAAATTATGAATTTTCTATTTAAAAATATTTTTAATTTATTGCGTTGATATAGCTGTTTGTATTTTCGGAGCATTTGTGATTAAATTTATCAACATTATCATGGTAAATGAACCACGTCAACGTGATAAACACCCAAACGATTAAGAAAAGAAGAAAAAAAAAAACTTGCTAAATAATTGTCTTTGAACGAACATAAAAAAAAAGAAATCTGATGTTTGTTTTCATATTTTGTTGAAAAGCTGTCAGTCTTTGGATGATGTTGTTTTTTACATTCCCATACCGCCCATACCACCCATACCTCCCATGCCACCCATACCTCCCATACCACCACCACCCATCATTGGTGCATCTTCTTTTGGAAGATCACAAACAACAGCTTCAGCTGTTGTGAGTAATGATGCAACACCAGCAGCATCAGTGAGTGCAGTACGAACAACCTTTGTTGGATCAATTATTCCTTTTGCAATCATATCAACGTATTCATTATTAAGTGCATCATATCCCAATGTACCTTCACATACTTTTGATACAACAACACTAGCATCAACACCAGCATTTGAGGCAATTTGTTGACATGGCATACGAAGAGCATTTTCAACAATAAGAATACCAGTTGCTTGATCAGAATTTGATGGTTTCAATGATTTTAATGCTGGTGCACATCTCAAGAGAGCAGTACCACCTCCTGGAACAATTCCTTCTTCAACAGCAGCTCTTGTTGCATTAAGTGCATCATGTACACGATCTTTTTTTTTCATTAACTTCAACTTCACTACTACCACCAACTCTGAGTACAGCAACACCAGCTGATAAACGTGCAAGACGTTCTTGAAGTTTTTCTTTTTCATAATCAGATGTTGTTTCAGTTATTTGATCTCTCAATTGATCAGAACGTCTGTCAATATCATTTTTGGAACCTTTACCCTTTAATAATAATGTATCTTCTTTTGTTATAATAATTTCACCAACTTGTCCAAGATCAGCAGCAGTAACATCTTCAATTTTAACAAGATTAGCTTCATCACCAAATACAATACCACCAGTTGCAATAGCCATATCTTGTAATGTTGCTTTTCTATTATCACCAAAACCAGGTGCTTTAACAGCAGCAATTTGAAGACCAATTTTTAATCTATTAAATACAAGTGTTGTTAATGCTTCACCATCAATATCTTCAGCAATAATAACAAGTGGTTTTCTCATTGAATTTGCTAATTCAAGTGCTGGTACAATACTTTGAATTGTTGATATTTTTTTTTTCACTATATAAGACAAGTGCATCTTGAAATTCAACTTTAGCACCTTTACTTGAATTAATAAAGTATGGTGATATATAACCACGATCAAATTTCATACCTTCAATAACTTCAAGTTCATCATTTAATGTTTTACCATCTTTAACTGTTATAACACCCTCTTTACCAACTTTTTTCATTGCATCAGATATTAATGTACCAATAGCAGTATCACCATTTGCTGATATTGTTGCAACTTGTGCAATTTCTTCTGGTGTTGTAACTGGTTTACTTAAATTTTTAAGTTCTTCTTTAATTTTATCAACAGCCATCATAACACCACGACGTATTTCAATTGGATTTGCACCTTTACTTATTTTTTCAAAACCTTCTTTAGCAATAGCACGTGCAAGAACAGTTGCTGTTGTTGTACCATCACCAGCTTCTTCATTTGTATTATTAGCAACATCTTGAACAAGTTTTGCACCAATATTTTGAAATTTATCTTTAAGCTCAATGCCTTTAGCAACAGTAACACCGTCTTTTGTTATTTTTGGACTACCCCAGCTTTGTTCGAGAATTACATTACGTCCTTTTGGACCCATTGTAACTGCAACAGCATCAGCAAGAACATCAACACCTTGGAGCATAAGGGCACGTACATCAGCACCAAAACGTACATCCTTTGCGTAGTTACGACATTGAAGTTGAAGTTGACGTGATGCAACACTTCTCAATACAACTGGTAGTCTATACATCTTTGTGATGATTAAGTAGTAATTAAATTAAATAAAAGAAATTTATTAAATATATTTTTGAGTAATATTAATATATAATTGTGATAACACACGTTTTTTACTCTGTACTGAGATCAGAGAAGGAACAAGCGGAAATGATGGTTGAACTCTGG

>CL859.Contig2

CGGTGTTCATTTCTTATTCTCTCAAGTGTCGTGTTTGTGTTCGGTTATATATTCGTTCTGACTACTCGTCTTCGTCATCACTCATCATAGTCGAGATTCGCCGGGTCCTGTTCAACATACAATTTAATTTTTATTGTTTTAAACCTCCCGTTGTAAATCTATTCAATTTGTGCTCATTGTTTTGAAATCAAAACCTGGGTATGGACCACCGAAAGTCCATTTGTGCTTTTTGGGCACTATTATTTTTAGTTAGCCCTATTGTGTCTAAGGAAGGAACAAAATCTAAAGATGAACTTGGTACAGTTATTGGAATTGATTTAGGCACAACGTATTCATGTGTTGGGGTATATAAAAATGGTCGAGTAGAAATAATAGCCAATGATCAAGGTAACAGAATTACACCATCGTATGTTGCATTCACCAAAGAAGGAGAACGTCTTATTGGGGATGCAGCTAAAAATCAGCTTACTACAAATCCTGAGAATACAGTATTTGATGCTAAGCGATTAATTGGTCGTGATTGGTCTGATGTCAATGTTCAACATGATGTTAAGTTCTTCCCCTTCAAGGTGGTTGAAAAAAATACTAAGCCTCACATTGAAGTTGAAACTATTGAAGGTACATCCAAAGTGTTTGCGCCTGAAGAAATTTCTGCTATGGTATTAGCTAAGATGAAGGAAACTGCTGAAGCATACTTAGGCAAAACTGTAACACATGCAGTCGTCACTGTACCCGCTTATTTCAATGATGGCCAACGTCAAGCTACTAAAGATGCAGGAGCTATTGCCGGTCTCACAGTCATGAGAATCATTAATGAGCCAACAGCTGCAGCTATAGCATATGGTTTAGATAAAAGAGAAGGAGAAAAAAATGTTCTTGTATTTGATTTGGGTGGTGGTACCTTTGATGTATCATTGCTGACAATTGATAATGGTGTCTTTGAAGTAGTATCAACAAATGGAGATACTCATTTAGGTGGAGAAGATTTTGATCAAAGAGTTATGGATCATTTCATTAAGTTGTATAAGAAAAAGAAGGGTAAAGACATCAGAAAAGATAACCGTGCAGTTCAAAAATTGAGACGTGAAGTAGAAAAGGCAAAACGTGGTCTTTCTGCCAGTCATCAAGTTCGCATAGAAATTGAAAGTTTCTTTGAAGGAGATGATTTCTCTGAGACTTTGACTCGTGCCAAATTTGAAGAACTTAACATGGATTTGTTTAGATCAACCATGAAACCCGTTCAAAAAGTTATGGAGGATGCTGATATGAATAAAAAGGATATTGATGAAATTGTGTTGGTTGGTGGTAGTACTAGAATACCTAAAGTTCAACAGCTAGTCAAAGAGTATTTCAATGGAAAGGAACCATCACGTGGTATTAATCCAGATGAAGCTGTTGCTTACGGTGCTGCTGTTCAGGCTGGAGTTTTATCAGGCGAACAAGATACAGATGCAATCATTCTTTTAGATGTAAATCCTTTAACTATGGGTATTGAAACTGTTGGTGGAGTTATGACCAAATTGATTCCACGTAACACAGTTATTCCAACCAAGAAAAGTCAAATTTTCTCCACTGCTGCTGATAATCAAAACACTGTTACAATTCAAGTATTTGAAGGTGAACGACCGATGACCAAAGACAATCATCTTTTAGGAAAGTTCGATCTTACCGGAATACCCCCTGCACCTAGAGGAGTTCCACAAATTGAAGTAACATTTGAAATTGATGCTAATGGTATTCTGCAAGTTAGTGCAGAAGACAAGGGAACTGGTAACCGAGAAAAGATTGTCATTACCAATGATCAAAATAGATTGACTCCAGATGACATAGAACGTATGATCAAGGAAGCTGAGAAGTTTGCTGATGATGACAAGAAATTGAAGGAACGTGTTGAATCTAGAAATGATCTGGAATCTTATGCATACTCATTAAAGAATCAAATCGGCGATAAAGAAAAATTAGGTGGAAAGTTGTCAGACGCTGAAAAAACAAAAATGGAAGAAATTATTGATGCTAAGATTAAATGGTTGGATGAGAATCAAGATGCTGATCCAGAACAATACAAAACACAAAAAAACCGAATTGGAGAGTGTTGTCAACCCAATCATATCAAAATTATATGCTAGTACTGGTGGTGTTCCTCCTCCACCTGCTGGAGATGCTGAGAAAGATGAACTTTAAAAATGGAAATATTTATAAATTTATTTATTGTATATAAGACTGCTAAGGTTTTACTTCCTTTTGGATTATGTATGTTAAGCAGTTAGGTTAACAATTAGCTAATATATTTTTTTCTATGTATGTAATTCATCATTTTATAAATGGTTCGAATTATTTGGTTTTTGTACAATAAACATTTAAATCCATTTGATAACACCGTTAATTAAGCAGGATTAATTTTATTATTGAAATTCTGTCAATATTATGTACAAATTATATTAACTATATTAAAAATTCATCAATTTGGAACAATAAATTAACATGTTATTCTGTTTAGAAATACTTATAATTTATGATTAATTGCCTTTCA

>CL966.Contig1

TGTAAAATCACGTTGTGCTCAAAATATTAAGTATCTATTTACTTATATGAAGTAACTAATATCAAAATATTTTTTCTTATTAAGTAGTTTAACTGAACATAAAATGTCTTCAATGGCAGCGGTTAAATTGTTTAGAAATCAACTCATCAGAAATGTTTGGCGTTTTAGGAGCTCCGGACAAATATACAATTTTAGACCGAGTAATGTGTTATTGTCATCGAGTGTGCCCATTAGAAATTTTGCAGCTAATCCTAGTTTGGATGATAGTGTGAATGAAAAAAATCCAACTGCAGAAAAATTGGAATTTCAAGCGGAGACACGTATGCTGTTGGATATTGTTGCCAAATCGTTATATTCTGAAAAAGAAGTTTTTATCAGAGAATTGATTTCAAATGCTAGTGATGCGTTAGAAAAATTACGTTATGTTAGCTTAAAAGCTGGCGAATCGCATGACTTAGGAAGTTTAGATATAAGCATAACTACTGACAAGACAAACCGCAAATTAACAATTCAAGACACAGGAATTGGAATGACTAGGGAAGAGTTGATTTCAAATTTGGGTACTATTGCTAAGTCTGGTTCCAAAGCATTTTTACAAAATATTCAACAAAGTGAATCAACAAATTTATCATCAATTATTGGTCAATTTGGTGTTGGTTTTTATAGTTGTTTTATGGTAGCTGATAGAGTAGAAGTTTTCACTAAATCTATGAACCCTCATGCCACTGGATACTTATGGAAATCAGATGGAACTAATGAATATGAAATAATAGAATCTGATAATATAGAGAGGGGTACAAAAATAGTCATGTATCTTAAACAAGATTGTCGCGAATATGCTTCAGAAGAAACTGTTCGTAATGTAATTAACAAATACAGCAATTTTGTGAATTCTCCAATCAAGTTAAATGGTTCTGAAATTAATACCATACAACCATTATGGTTACTCAATCCCAAATCCATTTCCAAAGAGCAACACGATGAATTTTACAAATTTGTAGCAAACACTTATGATAGACCAAGATTTGTGTTGCATTATTCTGCTGAAGCTCCAATTCAAGTGCGTGCATTGTTATACTTTCCAGAACTAGCACCAGATCAGACTGATTTTTATGATAGTTCAACTAAAGGTGTTTCACTTTATACTAGACGTATATTGATTAAAAAAGAAGCTGAAAATGTATTACCCAAATGGCTTCGTTTTGTAAAAGGTGTTATTGATTCAGAAGATATACCATTAAATTTAAGTCGAGAAATGTTACAGAACTCTTCATTACTTAGAAAATTGAATCAATTATTGACCAATAAAATTATTAAATTTTTACATGATAAATCTGTGAAAGATCTTGATGAATACATGAATTTTTATCAGGATTATGGTGTGTTTATCAAAGAAGGAGTTGTAACAAGTGATGACCCCAAAGAACGAGAAGAGCTTGCTGGTCTTTTAAGATATGAATCTTCATATACTAAACCTGGTGAAACAACATCATTTGAAGATTATATGAAGAGAAGACAAGAAGGTCAAAACGATATTTACTTCTTATCTGCTCCTAACCGCACTTTAGCAGAAAGTTCACCGTATATAGAAGCATTAAAAAAGAAAAACATTGAAGTTATTTATTGTTATGGATCACATGATGAAATTGTTTTATATCAAGTAAAAACATTCAAGAATGTCAAACTCACTTTAGTAGAAAAAGAAATTAATGCTGCTAATAGCCAAAGTTCTCAAGCAGTTGACTTAGGAGCTGATAGTTTAAGTCAGTTACAATTAGATTCTTTATTACCTTGGATTGAATCCGTATTGGGTAATAAAGTGAAAAAAGTAAAAACTACTGGACAATTAGAAAGCCATCCTTGTGTAATCACTGTTGATGACATGACAGCTGCTAGACATTTCATTAAAACTCAATTAAAACAAATGGATGACGAGATGTTATTTTCAGTGCTTCAGCCTCAACTTGAACTAAACCCTAAACATGCAGTCATAAAAAAATTGCATACACTTAAAGATTCAAATCCAGAATTGGCTAAATTAATTGTGGAACAGTTGTACAGTAATTCCATGGTAACAGCGGGCCTCATCAATGATTCCAGAAAATTGGTGTCCAACATGAATCATATTCTAGAATTAGTGGTTGAAAAATACTAAATTGTTGGTTATGTAGTGAAACACCCTTATAATTTTGTAAATATTATTAGAAATAAGTAATATATAATTATTATTTTTTTTTAGTAAAAAA

>U10036

CTAGAATATTCTTCCGATTTCCACACTTCCACAGTACTTTGATTGTTTAGGATATTGAGTTCCTATTCGTTTCAATTTGGATTTTTAATCATTCAAAGTTACAACGCTTCCTCGTGTACTTCGATTAGTCCGATTCAGAATTAGCATTATATTAAATAGTATTTACCAAGCTATTTAATACTTATAGTTTCTTATTCATAATTAATTTTTATATTTTTAAATCAAATCAGCTGAATAAACTACCTTTTAAATTATGGCGTTAGATTTGAAGGAAAAAGGTAATGCCGCCTTGGCAATGGGAAATTATGAAGAAGCTATTTTAGATTACACTCAAGCTATTGCTTTAGACCCCAACAACCATGTACTTTATAGTAATAGATCGGCTGCATATGCAAAGCAAGGCAATTACCAAAATGCGTTGGAAGATGCTGAAAAGACTCTGTCTATTAAACCAGATTGGCCTAAAGGATATTCAAGAAAAGGAACAGCTTTATCATTTTTGGGTCGTAAAGATGATGCTGCCAAAGCATATGAAGAAGGTTTAAAGTTTGACCCTAGTAATCAGCAGTTGCTGGATGGCTTAAAAGAAGTGAAACAAACTCCTCAATCTCCTTTTGGAAGTAATATGTTTCCAGCAGAAGCTTTCTTAAAACTTGCACAGGATCCCAGAACTAAAGATTTGATTAATGATCAACAATTCATGAGTCTACTTATGGAATGCCAAAGAGACCCTCAAAAGCTAATTATGAATATGCAAGATCCTAGAATATCAGCTGCATTAAGTGTGATGATGGGTATTAATTTAATGGGAGATGATGACAAAATGGATACTGAACCGTCACCTCCACCTCCTCCAAAACAAAAAAAGAGAACCAACACCACCGCCCCCTGAAAAATCTGAAGATGAAAGTTTAACAGAAGAACAAAAAGAGGCTAAAAAAGAAAAAGACTTGGGCAATGAAGAATACAAAAAGAAGAATTTTGAATCAGCTCTTGCACATTACAATAAAGCTATTGAGTTAGAACCTACAAATATGACTTTTTACAACAATGTTGCTGCTGTTTATTTTGAACAAAAAGAATATAAGAAATGCATTGAGCAGTGTGAAAAAGCAGTTGAAGTAGGCAGAGAAAATAGAGCAGATTTCAAATTAATTGCCAAAGCTTTTTCTAGAATTGGAAATGCTTATAAAAAACTTGAGGACTACAAAAGTGCAAGAACATATTTTCAAAAATCAATGTCAGAACATAGAACCCCAGAAGTAAAGACTATTATTTCTGAATTAGAGAAAAAAAATAAAAGAAGAAGATCGCAAAGCATATGTAGACCCAGTAAAAGCTGAAGAAGCTAAAGAAAAAGGAAATGAATTTTTCAATAAAGGTCAATTTGCTGATGCTGTCAAATTTTATAGTGAAGCAATTCTGCGTAATCCTGATGAACCTAAATATTATAGTAATCGGGCTGCTTGCTATACTAAATTAGCTGCTTTTGATCTTGGTTTAAAAGACTGTGAAAAATGTGTTGAATTGGATCCTAAATTTTTAAAAGGATGGATAAGAAAAGGTAAAATTTTACAAGGTATGCAACAATCTTCTAAAGCTTTATCAGCATATCAAAAGGCATTGGAAATAGATTCATCTAATACTGAAGCCTTAGAAGGTTACCGGTCATGTTCTATAGCTGCAAATTCAGATCCCGAAGAAATGCGCAAGCGTGCTATGGCTGATCCTGAAGTTCAAGATATTATTAGAGACCCTGCAATGCGTCTTATACTTGAACAAATGCAGAATGACCCTAAAGCTTTAAGCGACCATTTGAAGAATCCAGAAATTGCTAGTAAGATCCAAAAATTAATTGAGTCTGGATTAATCGCCATTCGTTAAATTTAAGAATGTTTGTACATCTTACACTACTTTAAATGCTTTTTTATAAATTTATATTATATTTTTGAATTTTCTTTCCAATTATATAATATACAGTTAGGTATGATAATAATTAAATTACAACATGAAATATATGATATTTTAGGGTTATTTAATACTGTTTAATATTTTTCAACTTTTATATAATGTTCTATATGACGTGTAACTGTGACTTTAGTTTTGAATATTGTTGCAAAGAATAAGATATAGATTACACAATCGTGTTAACTAAAATAAAAATTATGAATGATTAAGTTAAAAAATGAGTACTAAACCCAATTATTATAGGTTATACAACTTATTGATATTTAAAGCTTCTTCGCACACACTAGACTTGAACCAGCTGGACCATCGAACTGAAAGAAATCAAAATACCTATTTTATTTTTATGAGTGTTACACATTTACCAAACTGATCGCTCAACCTGTAATTTGGTTTCAAATATTCAAATTGTTTGAATTTATCAGCTAAAAAAAATGTTTCGTTTTAGTTAGATCTAGCCGGTTTAAGTCTAGAGTATGTGTGACTTTATTATACTATTAAAAGTAAATATGATATAATTACTTTTAAAGTTAGTAAAAAGATGCTGCGTTTTCAAATGAAATTATATTTTATTCACAACTGCTATTTTTAGATATTTAAAATTTGATTTAAGTACA

>U12560

CTTAATCATGATTCCGGGCAACTCACGGTTTGCTCTTTCTCATTTCTAGTTTTTTTATTTCATGAGTTTTCGTGTTTCAATTAGGTACTTTTAATTTTCGTTGCTCTTGTATAGAGTGGCTTGACGAACGAAAACGTCGAAAACGGGATCATGTCTTTCATACCCGGGTCGTTGGGTTTCGATGAATTCGGTACGCCGTTCATCATCATCAAAGACCAACACAAACAGCGTAGGCTGACTGGCATCGATGCGTTGAAGTCTCACATTATGGCCGCACGGTCTATCGCCAAGACGTTGTACACATCATTGGGTCCAAAAGGTTTGGATAAAATGATGGTTTCATCTGATGGTGAAGTGACTGTTACCAATGATGGTGCCACCATTTTGAATATGATGGATGTTGATCATGAGATCGCTAAGCTTATGGTACAGTTGTCTCAATCCCAAGATAATGAAATTGGTGATGGTACTACTGGTGTTGTAGTCATTGCCGGAGCTTTGCTCGAACAAGCTGAACATTTGCTTGATCAAGGTATACATCCTATTAGAATTGCAGATGGTTTTGAATTGGCTGCTCAAAGTGCTTGCAAGCATTTAGACTCTATTGCTGATTCATTTCCAGTTGATATCAACAACTTGGAACCTCTTATTAAAACAGCAATGACAACTCTGGGATCCAAAATTATTAACAAGTGCCATCGTCAAATGGCTGAAATAGCTGTAAATGCAGTTATATCTGTAGCAGATTTTGATAAACGTGATGTCAATTTTGAGTTGATCAAAGTCCAAGGAAAAGAAGGAGGAAGATTAGAAGACACAATGTTAGTGAAAGGAGTTGTAGTTGATAAAGACTTTTCACATCCTCAAATGCCAAAGGAGCTTAAAAATGTTCATCTCGCCATTCTTACTTGTCCGTTTGAGCCACCTAAGCCAAAGACTAAGCATAAGTTGGATGTCGCTTCAGTTGAAGACTATAGAGCATTAAGAAAATATGAACAAGATACATTCAATGAAATGGTTAAAAAAGTGAAAGATGCTGGTGCTACATTAGCAATTTGTCAATGGGGTTTTGATGATGAAGCAAATCATTTGTTATTGAAACATGAATTGCCTGCTGTACGTTGGGTTGGTGGACCTGAGATAGAATTAATTGCAATTGCTACTGGAGGTAGAATCATTCCTCGTTTTGAAGAAGTTTCTAAAGAAAAATTAGGTTACGCTGGTAAAGTTAGCGAACTTTCTTTTGGTACTACTAAAGACAAAATGTTGGTGATTGAAGAATGTAAGAATTCACGTGCTGTTACCATATTTATACGTGGTGGAAATAAAATGATTATTGAAGAAGCCAAACGCAGTATTCATGATGCTTTATGCGTTGTTCGAAATTTGGTGCAAGATAACCGCATAGTTTATGGTGGTGGTGCAGCTGAAATTTCATGCGCTATTGCTGTTTCTACTGAAGCAGACAAGATTTCATCTATTGAGCAATATGCATACCGGGCATTTGCAGATGCTTTAGAAAGTGTTCCATTAGCTTTAGCAGAGAACAGTGGATTGTCACCAATTGATACGTTAACTCAAGTTAGAGCCAGACAAATCAAAGAAAATAATCCAGCTCTTGGTATTGATTGTATGCTTGTTGGAACTAATGATATGAAAGAACAAAATGTTATTGAGACTCTTCATAGCAAGAAACAACAAATCATTTTGGCTGCACAGTTAGTTAAAATGATTTTGAAAATAGATGACGTCAGAGTTCCAGGAGAAGATTTTTAATTTGATTTTAATATCTTAAAAACACTAAACTGATGATCAGCGCTTACTTGTTTTCCTAAAACTTGTTCTTTTTTTATGTTCAAAATTTACATTAATAATTTTATAAGTTATTTATAATTTTTTTCTAATAACAAAATACACTACTAATCAATTAAAATTGGACGTATATTTAACTTCAAACTATAATTCTAACATGATTACAATATTCGCCACAAGAACTATTTTTTCATTGAAGCACACAAAATGGCACTTTTCGTTCTTTCATGTCATACTCGTCATTGTTTTCTTTCATGCCGTCAGCAGACATGCCTGTGCCCCCCAACACACCGGGGGGGCACACTGTTTGTCATCTCATTGCTAGAGTTGATACCATTGGTGGTCGTACTGGCTTATTGTCCAATATACGCTCCAATTCGGCCATTACATTTGAGTTGAGTTTTGGCAACAACTAAATCAGAAAATAATATAAAATGTTTGGTGTATGTGTGTTAAAATAATAAATTATATTTAATTATTATTTTTATTAATTATTATTATTGCAATAACATTTTGAAATAAATACATTTTTTAATATAATAAAAAAAA

>U13800

AGTAGACCCATAACAACCCCAAAACCAGAAAAAAAAATCGTATATTCTGCGCGCCCTCCCCCCCCCTATTTTGTGGCCGTAAAATAAAACATACGCCTATTGCGCGTACGGCGCAAATTACGATGATAGTTGAAGGTGCACGTTTTGGCCGCGAATCAGAAGTTCCAATATTATGTACACTCATCGACCCCATAGATAAAAATTAGGAGTAGCTAAGCGGGACTAAGCAGCTAAAAAAATATTTTGCTTTCTCCACCCTAAAAACATTGATCAGATACACCATACACAGCGTAAACATTATACACCACAAAAAAGATATTTATCGAATTTCCCCAAAATATAACCAGCTACCCCCTCAAAGAAAAAAAATGTGCATCGCATACCATATTTATCACACATCATAACTTGTTATTATGATACAACTCGAGTATTCCTAGTAGTATGCGTGTAAAGATTGAATTCATCATATATGATTTCATTATAGAGAAAAACCTGCAAAAT

>U14240

GTCAGACGAGTGTACACAAACCTTATTACAGTCGTGAAATACCGTGTTATAATAAGAGAGCTGATGTGACACCGTAATACTTGCATGCCTAAACAGTAAACGTACTATAGTTTATTAATTATAGTTCATCCATCGAGTATAATATAAGCACAATTTAATTACATCAATCATTCAACCGTATAATGTCGGTCTATGAAAACGTAGACTACGATGGCTTGTTGGCGAAAATGGACCAAAAATCGTTTGTGATTGATGTCCGTGAACGCCACGAGCTTGCTGCGACTGGATCATTACCCAACAGCATAAACGTTCCACTCGGCGAGTTGGAAAACGATTTAAATTTGTCCGCTGAAGTATTTGAGAAAAAATACCAAGTGCCCAAACCTGACAAAGATAGTAGCGAAATCGTATTCTCCTGTGCCCGGGGAAATAGAAGTCGCCGTGCAGCTGAAATCGCTTTCAAACTAAATTATAAAAAGTTATTTAATTACACAGGCGGTTGGGCCGAATGGTCGGTGAAATATCCAAGTACATAATATAATTTAATACTTGAAAATTGGAATCAACTAGTATGAAATATACTAAATAATTAAAGGTACATATTATTCTCTAAGTACCTTTCCGAGAGGAAGCAATGCGAAAGCGGTCCCCTCCCGGTCGTCGATTTCAACCCGGAAAAAAAAAAAAGTACCTAGTTTAATATCATAATTTATAGCATATTTCTTTGTTTCTCAATGTCTATAGACATCTGTGATTTCATTTTATATAATTTTAATACCTAAAGTAGCTTGAGTTTCATTA

>U14667

GTACCGTTCGGCCTCGCTGAGCATGCGATCGATTTCGGCCTGAGACAGGCGACCCTTGTCGTTTTTGATGACGATGTTCTTGGAGCGTCCGGAGCTGTTGTCCTTGGCCGATACGTTCAAAATCCCGTTGGCGTCCAAATCGAAAGTCACTTCGATTTTGGGCACGCCTCTGGGCGCCGGAGGTATGCCGGTCAGGTCGAACGTTCCCAACAGA

>U16470

TGTATTTTTATTTTTATTATCTTACATTTTAAGAAGGAAATAATTTATGATTTTGATCATTCATTGATAGTATTTGAAATTGTTTCAAAATGATTATTAGCCATTCCTTTACTTTTATTTAAATGAGTCAACTTAAGCTTACCCTACTTGAAATACCCAATAAGCGTTAATAGATTGATTTCAATTTTAATCTATTACAATTCAAA

>U17311

GCAGGGTTTGTGGAAGAAGACTTAAGACCAGAAGAACATGATCCGGAATGGTGTTTACAAAAAGGAAATACTTTTATGACAGAACTAAATTACATTGGAGCCGTAAGTGCTTATTCACATGGAATAAAGTTGAGCCCAAAAATGGCTGTATTATATTTGAACAGATCTAAAGCGCAAGTTCAAATGAAAAATTATCATAAAGCAGTCGAAGATGCAACAACTGCGTTGGAGTTGTTGGTGCCAGCGGTTGAAGGAAACGTGGTTTGGCGAGCCGAGGCGTATTACAACCGAGGCAAGGCGCTGGTCGAACTAAATACTGCACATTTAGCTATCGATGAACTCAGGTTGGCTTTGACATTGGCACCAGACCGGGCTCATCTCTACGGACCGGAGTTGCAG

>U17685

ATTACATTTTGAACATATGTTTAGTTTTTTTAAATAATAATTTAAATGAAGAGTGAAGCTCACAGGTGATTAAACACATATGTAGCATCCTTAAATTGAACACCTACTTACGAATATGTGCTTATCACATTACGATGATAAGTCCATGGACCAATATACAAGTCATTAGTTTTTACACCTTTTTTAATTCCCTCTTCGCCATATTCATCATATATAGCTCTGCGAAATGGGTCAGATAATACCTCATATGCTTCACACAACATAACCAAACAACAATTTTCAACTTGAGTTTTTACTAGTTTTCCAAATCTAAAATCATGGTAA

>U20698

TTGCATCATTACACGTGAAGTCCGCACTTGGATTAAAAAAAATTGTGAATTAATATTTTATAAATTGTTTGAAAAGTTATTAATTAATTAACCAATTAAAATGTTGGCTGCATCAAGATTATTGACACGCAATTGCGTTGAAATTTCTCAAGGAAAGAGATGTTTTAGTGCATTAGTTAAAAATGCCAACTCAACAGCATACTATGCACCCAACAACAACAACGAAGTTACTCAATACAGATATAAATCAGACAAAGTCAGTGGTAATGTTATAGGAATTGACTTGGGTACAACATTCTCATGTGTATCTGTTTGGGAAGGTGGTGCAGCTAAAGTTATTGAAAATTCTCAAGGCTCAAGAACAACACCATCATACGTTGCCTTTGCTGAAGATGGTACTCAACTTGTTGGTATTCCAGCAAAACGTCAAGCCATCACCAACCCACAAAACACATTTTATGCTACCAAAAGATTGATTGGACGTAAATTCAATGACCCAGAGATTCAAAAGGAAATTAAAACATTGTCATATAAAGTTGCTCCAGCATCAAACGGAGATGCCTGGGTTCAAGATAAATCTGGCAAATTACATTCCCCAAGTCAAATTGGAGCTGCTGTATTGAGAAAAATGAAAGAAACTGCTGAATCATATTTACGTACACCAGTTAAAAATGCAGTCATCACAGTACCAGCTTATTTCAATGATTCTCAACGTCAAGCAACCAAAGATGCTGGTCAAATTGCTGGTTTAAATGTTCTCAGAGTTATCAATGAACCAACAGCAGCTGCTCTTGCCTATGGCATGGACAAAACAGAAGACAAAATCATTGCAGTCTATGACTTGGGTGGTGGTACCTTTGATATATCAGTTTTAGAAATTCAAAAGGGTGTCTTTGAGGTCAAATCAACAAATGGTGATACATTCCTTGGTGGTGAAGACTTTGACAATACTCTTGTCAATTATTTGGCCAGTGAATTTAAAAAAGATCAAGGAATTGATGTTACCAAAGATCAACAAGCTATGCAACGTCTCAAAGAAGCTGCTGAAAAAGCAAAGATTGAATTGTCTGATGACTCACAGACTGACATCAACTTGCCATACTTGACCATGGATGCTACTGGCCCAAAACATTTTAATCTTAAACTTACAAGAAGCAAGTTTGAAGCACTTGTTGCTGATTTAATCAAACGTACAATTCAACCTTGCCAAAAAGCTATCAGCGATGCTGAAGTCAGCAAATCTGAAATTGGAGAAGTACTTCTTGTTGGTGGTATGACAAGAGTACCAAAAGTTCAACAAACTGTACAAGAAATCTTTGGTCGTCAACCATCCAGAGCTGTTAATCCTGATGAAGCTGTTGCCATTGGTGCTGCTGTACAAGGTGGTGTTCTTGCTGGTGATGTCACTGACGTTCTTCTTCTTGATGTTACTCCACTCTCACTTGGTATTGAAACACTTGGTGGTGTATTTACAAAATTGATCACACGTAACACAACAATTCCAACAAAAAAAGAGCCAAGTCTTTTCAACAGCTGCTGATGGACAATCACAAGTTGAAATCAAAGTACACCAAGGAGAACGTGAAATGGCTGCTGACAATAAATTGTTAGGTCAATTTACATTGATTGGTATTCCACCAGCACCAAGAGGTGTACCACAAATTGAAGTCACATTTGATATTGATGCCAATGGTATTGTACACGTATCAGCTCGTGACAAGGGTACTGGCAAAGAACAACAAATATCAATTCAATCCAGTGGTGGTTTAAGCAAAGATGAAATTGAAAACATGGTCAGAAATGCTGAAAAGAATGCCAAAGAAGACAAAATCAAGAGAGATCGTGTCGAAGCTCTTAACCAAGCTGAAAGTATTATCAATGATACTGAAAGTAAAATTGAAGAATTTAGACCACAATTACCAGGTGAAGAGTGTGATAAATTAAATGGTCTTGTTAGCAAACTCAAAGAAACACTTGCCAACAAAGACAACGCAGACCCAGAAGAAATTAAAAAAACAAACAAATGAATTACAACAAGCTAGTCTCAAGCTTTTCGAGATGGCATACAAAAAAAATGGCTGCTGAACGTGAAAGCAACCAAGCTAACCAAGGCAACCAAGATCAACAACAACCAGAAGGTGAAAAGAAAGAAGAGAAGAATTAAAATTAACCAAGGCTTTATTAATTCTTCTCACACAAACTTATATTAACTTCTAATTATTATTATTATTTTCTTTGCTAATTTTATTTGGTTACTCATTACAATAATTACCTGAGTCAAGTCTTGTTAAATTAGAAGTTGTTAGGTTTCATCTGCTATCAACATCAGCAATATCAAATGTACTTGTTAGTTTTTAAATTCACTGACAAGTTAAAGTTCCAAATATTTTAATGTCACAAATTTTTAAATTGCAGTTTTAATTTTCAAAATATTGCATAATTAAATAATACAACTTTTTATTTCCTTTCTCAACAAATCATTAC

>U20769

AAAATGTACTTTAGCCAATGGATCCTGAGTATCTTTTGTTATTGCACGATAAAATTCATTGTAATCTTTTTCATCAACATCAATTGGTTTTAATGTCCAAATTGGTTTTGAATCATTAAGAACTTCCCAATCCCAAACTGTTTTATCAACCATTTTCTTTTGTTTTTCTTCATTTTCTTTAGGTGCTTCCTCAACTTCTACTTCTTCATTATCTGCTTTTTCTTCATCATTATCTGTAATATCATCATGATGTTCCTCTTTTTTCGTCTCTGAATTTGATACATCATCTTCAATGTCTTCAACTTGAATTGTTTTGCTGCTCCAAAGATAAATTGGGAAA

>U20815

TCAGTCTGCCTAAAAACAACAATCAATCCACGTTTATATTTTGTTTAAAAATAAATAAATAAATAAAGTAAACAACACTGTGAATAAAAAAACAGTTTAATTGTCAAGTTAGTGTGTAATTAAAAAAGCAAGAATAATAATGGCTTTACTCATACCAGATCCAAGTATCTTATCTCACTTTGATCTTGATGATGACGATGATTTTTTACGTGATATATTTTCAAGGAGAAAAATTCGCAGTCGTCTTGATGCCATTGCTCCATTACGTAAGCGTCCTTATTCACGAGCATTATCACACTCTCGTTTGTGGCCAGAACTTTTATTGACATCTGATGTATCAACACCAACTGTTAGACTTGACAAAAACAAATTTCAAGTCAATCTTGATGTTCATCAATTCAAGCCAGAAGAGATTGAAGTCAAGGTCATTGACAAATTCATAACTGTTGATGCTCATCATGAAGAACAACAGGATGAACATGGTTGGATCTCACGTAAATTTCAACGTAAATATTTAATTCCTGATGATTGTGATCCAGCTTTGATTAAATCAGCATTGTCATCTGATGGTGTACTCACTCTGACTGCACCAAAAAAAAGCTGAATTAACAGATGGAAATGAAAGAAAAATTCCAATTGAAATAACTGGTAAACCAGCTATTCGTGAGACTGAAAAATCAACAGATGCAAGCTCCAGTGAGACCTCAGTTGAAACGTCAACAGGCAACAAAAAAATCATCATCTGTTAAAGTTGTTCGTGATGAACAAGTTGGTCCCAAAGATGTCAAACGTAGAAAGAAGGATGTCGAAGCCTAAATCATCAACTTGATTTATCATTTTTTTTTTTCTTTAAATATTTATTTAATCATTATCTCAATTATTAATAATTCATATAAAAAATCATTGAATGATTTGTTTTTTTTTTTTTCGCTATTTATCTAAACTCGAGTGTTAATTTTATTATCATCTCTTTTGTATTTTTTCTATTAAAACATTATTGTACCTCCATTAATCACATAAAAAAA

>U20929

AAAAAAAAAAAAAAAAAAATTGTAACCATTTATTTTCTTACGGCAATTCATATAACTTGGCGCTGTAGCAGTTTGAATTTGCACGGCTCCGCCATATTGGCGATATCGACGATTTTACTTCGGATGACCTGTATATTGGGAATCATTCTAGGGACCTTGGACATGACGAAAAAAATTATTTTCATTTGCATCACTATTGCGCATATGTGACATTCAGGCATTGCTGTGGTATTTTGAAGTGTAAAATATTCATGCTGTTTAAAAGCAATTAATGTATTAAAATCTTCATCTCCATATACAAATAGTTTGGACGTACTAACATCATGTGAGGAGGATAACTTTTAATACTAAGATTACGTCTCATCGCAGAGTAATTTCAGACCAGCCATGTGGCTCATATTTGAGAAGCTATATATTGTTCGACTCGAAACATGATGGATTATAATTTATAATATTATTATGTTTAAGCAAAGCATTCTTATCTTAGAAATTAATCGTTATAAAATAGTATTGTTTTACAGAAAACAATTTAAAAATCAACGGTAAGACTTAATGCAGACTCCTAAGTACAGAAGAATTTTGCATATTGATAATATTTCATACGTAATATCGTTATACAATTTTTTTTTTATCGTCGAAATATTATCTTTTCTTTCTTATAAGCATTTGACATTTTTGATCATAATCAATATTATATACTGAAAAAACAGAGTTCATTTACATTAAAATCATGACACAAATTAAAAGGCCTCAGGAAAAGTGCGAAACTAAAAATGTTTGATGTGTTGCAAATGCTTGGGAGTATGAATACAGATATGAGATCTGTTTAATTATGGGCAATTGGAATAAGTTTTTCACCAGAACTTATTGCTGGAAGAGGGGCTTCAACAGTCAGAACACCATCCTTGCTGAGTGAAGATTTTATGCTCTCAGGATTAGTACCCTTAGGCAGTAGAAACTCTCGATTGTATTCTCTGTACACAGATTTGCTCTCTGTCTTTTCTTCATGTTGAGCATGAACCAAAAGTTTGTTATCCACAGTCTTGACAACGATTTCCTCGGGTGTGTATTGAGAAACGTCGAATCGCAATTTTAACATTTTGTTATCACCTTCATCTTGAATAAGAGGACTATTTAAACCATCCAACCAGGTGCGTGAATCATGATGTTGTGGACTGAGTGAACTATTCTGAGTAGATTGTTGAGTTGTAGTGCTTGTTGAGGATTTGAAGAAATTGTCGCTTTCACGATGCATAAGCTCACTTCTAAAACGGGACATTTCGTCTTCCATCTTCTTCATTTCAGCATCAAAACGCTCGCGAATATTAGAGAATTCGCTATCAATGACGCTGAAATCACCAAGTTTGATTGGAATGTTACGCTTAATACCGTTGTCAGCCATTTCAGAATCAAAAAATTGTAAATTATACAAAACTTTTAATTGTATATATTTGATTATAAAACACACTGAACGATTTGGTTACTCAGTTAAACTTATATGCATATAATCCGATGAATTTTTATTTCACTTAGGTAGCAACGTGCTTAATAATAAACTGGATCTCAGAATGAACGGCGCACGCAACAATTACTCCTTAAATAGCAACAGATAGGTTTAGTCGCTAGCTTGTGGGTGTTCGTTAAACATCGGTGATCA

>U21435

TACAATAAATGAATAAACAATTTAATTATATTAAATGAATTTTTTTTATTGTAACTTAAATTTACATTACAAATATTCTTTGATATATAATAATAATGTAGCAGTACAATGTTACAATAAAATTTAATAATAATTAATCAACTTTGTCTTTAACAAAATTTGTTGATTTATCAGTACAAGGACATAGTACCTAAATGACAATATATAAATACAAAGAAGTGAATAAGAAATATTAAAGTTGTGTTGTTTTTTTTAAACAATTAAACAACTTATTTTTTGGGACCATTTGTTTGTTCTTTTTGTTGTTTGATGGCTTTTTTATCATCTTCACGAATGGCTGGTTTACCAGTGTGTTCAATCTTGATGCTTCTCTCATTTTCAATGGCTTTTTCTGGTTTTCTTGGTACAGTTATTGTAAGTACACCATCTGATGACAATGATGAGTTGACCTTGGCAAGATCACACTGTTCTGGAATCAAATATTTACGTTGGAATTGACGTGAAATCCAGCCATGCTCATCTTGTTTTTCTTCATGTTTAGCTTCAACAGTGACGAATTTATCTTTGACCTTGACATCAATCTCTTCAGGCTTAAATTGTTGAACATCAAGAATAACTTGGAACTTGTCCTTGTCTGGTTTGACTGTTGATGTTGCTGATTCAGCATTGCGAAGAAGTTCAGCCCATGGACGTGCGTAATTAACAAATGGACGGGCAAGAAGACGTCGTTCAGCTGGTGACATATAAGCCTGGAGCAATTGTGGTGTCAACAATTGATCATGGCTCAATCCAACACCAAAATCTTGGTCAAAAACATGATGAGGATGATCCAAATCTTCCCACCAGTTTGAAAATAACATTGGTACTAATGACATCTTGGTTTGTTTATTAAATTAACTTTTTTTTTTAACAAAATGTATTGAAGATT

>U21550

AGGGTTGCGTGTCCAGATTGGTTTTGTCTTGTTCAAGACCTCTTCATCCAAGTACTTTTCTTTAATAGTCTTCTTCTTCTTTTTGTCTTTGTCTTCATCTTTTTTTGTCTTCGTCTTCATCCTCACCAACATCCTCAATTTTGGGTTTTTTGTCTTCTTCAGTTTCACCTTCAACTTCATCTTTCTTTTCTTCTTCAGCTTCATCATCGCTGACTTCTTTGGTACGTTCATTCTCAACGATTAATTTGATTGGGTAGCCAATGAATTGAGAGTGCTTCTTGATGATGCTGGTAATTTTTTCTTGTTGGAGGAACTCAGCT

>U24104

ATTGCCATATGATATTGCTGTGTTAAATATTGATTCAAGTGTCATAGATCCCACGTTGAGATCATTGACAATTGCCAATGACAATGCTAAAAAAGGTGAAACAGTTATTGCTGCTGGTTTTCCATTTTTTTTCATCAACATTACCAACAATAACACGTGGTAATATATCAAGTGTATCAATAAATATGATACAAACCACCTGTTGTGTACAAAGTGGTGCTAGTGGTGGACCTATTGTTCGTCCATCAACTGGTGAATTACTTGGTATCATTGTTTGTAATGTCATAACATCAACAACACTTTATCCAAAGCTTAATATGGCTATTCCTGCTTCAATTATCAAAGGACCAATCGATGAGTACATCAATACTGGCTGTGTTGAAGCACTCGAGTCACTAACAAGCCAAGAAATATCAACAGAAAAAGCATGGA

>U24336

TAACACAACCATCCCAACTAAACAGACCCAAACTTTCACTACCTACTCTGATAATCAACCTGGAGTCTTAATTCAAGTATATGAAGGAGAACGTGCTATGACCAAAGACAACAATTTGTTGGGAAAATTCGAGCTAACTGCTATTCCTCCTGCACCACGTGGAGTCCCTCAAATTGAAGTCACCTTTGACATTGACGCCAATGGTATTTTGAATGTTAGCGCTATTGAAAAGTCCACCAACAAAGAAAATAAGATCACCATCACTAATGACAAGGGACGTTTGAGCAAGGA

>U29115

AAAAAAAAAAAAAGATGTTTCGAGAATAATTCTCTACAGCCAACCAGAATTAACACAGTGAGGTTACTTCTCAAATAATCGTAGCAACGTGTACGCGTGATTGCCGGTCGATCACTTGATTATCATTACAAATAAATCATTAATTTACATTTAAAAAAATACAATTAAAATGTCTGCAGCAGCATCATTTGCAAAGAAGGTATTCCCACTTCTCGACAGAGTTTTGGTCCAGAGAGCTGAGGCAATCACCAAGACCAAGGGTGGTATTATCATTCCAGAAAATGCACAAGCTAAAGTTTTGAAAGCAACTGTTATTGCTACTGGACCTGGAGCACGTAATCAGCAAGGAGAACATGTTCCTTTGAGTGTCAAAGTTGGTGACACAGTTCTTCTTCCAGAATATGGTGGTACCAAAGTTAACCTTGAAGAAGACAAGGAATTTCATCTTTTCCGTGAATCTGACATTCTTGCCAAAATTGAATAAAATTATCAACCTAGGCTTAAAACAATTTCTTAATTTTTTTTTTTTATTAATTTCATCATAATCTTCAAAAATAATTACACTTGATCGTCGTG

>U29653

GGTGTTGTTCTGTTGCCTTGATCATTTGCAATTATTTCAACTTTTCCTTGTTGCCATACACCAACACAGGAGTATGTTGTACCCAAATCAATTCCAATTGCAGGCATTGTGATTGTTTTTTTTTTTATTTTTAAACACTTTAACTCTTTCACTTGTAACTTGTTCAATGTTTGTTTGTAAACTGATGTTGGATCTGTCAG

>U29920

GTTTATAAGTTATAACCTTTTAAAGTTTAGATGAGCAGAGTGAAGAGATACTGATATGCACTGCAAAATGTTAGTATTCTAATATTCTATTTTTTTTTTTCAGATCAGCTAAATCTTTAATCCGCTACCCATTAAGAGACATATTTCTATTATTCTGGGAGTACAGCAAAAATATTATATATAATGTAATAAGTCAGCCCATTTGAAATATTTAATTGTGGTATTGATTTAAACATTTTAACATAACTGAAATAAAAATCAAGTTTTTTATTTACCATGTGATACCTAATCATAACCTGCTAGTCATACT

>U31500

CTTGTACTTGCGTTTGAACTCTTGCACAAAGTGATTGACCATTCTATTGTCAAAATCTTCTCCACCCAAGTGAGTGTCTCCGGCAGTCGATTTGACTTCAAATATACCGTCTTCAATGGTCAAGATGGATACATCAAAAGTACCACCACCCAGATCAAAGATCAAAACGTTACGCTCTCCAGATGTTTTCTTGTCTAAACCATATGCAATGGCAGCTGCTGTAGGCTCATTGATGATGCGCATTACGTTCAAACCAGCAATGGTACCAGAATCTTTGGTAGCTTGACGTTGGGAATCATTGAAGTATGCAGGAACAGTGATTACAGCATTTGTTACTGTTTTTCCTAAGTAGGCTTCAGCAGTTTCCTTCATCTTGGTTAAAACCATTGACGATACCTCTTCTGGGGAGAAGATTTTGTTTTCTCCTTTGTAGGAAATTCTGATTTTGGGTTTGCCACCATCGCTGATTACCTCAAATGGCCAATGTTTCATGTCAGCTTGTACAGTAGCATCTTCAAAACGTCTACCAATCAATCGCTTGGCATCGAAGATTGTGTTGTTGGGGT

>U33216

AGCTGTTGCTTATGGTGCTGCTGTACAAGCTGGTGTATTATCTGGTGAACAAGATACAGATGCAATTGTACTTCTTGATGTTAATCCACTTACTATGGGTATTGAAACAGTTGGTGGTGTTATGACAAAATTAATTCCAAGAAATACTGTTATTCCAACTAAAAAATCACAAATATTTTCAACTGCATCTGACAATCAACACACTGTTACAATTCAAGTATATGAAGGTGAACGTCCAATGACCAAAGATAATCATCCACTTGGTAAATTTGATTTAACTGGTATTCCACCAGCACCAAGAGGCATTCCACAAATTGAAGTTACATTTGAAATTGATGCCAATGGTATTCTTCAAGTATCTGCTGAAGACAAAGGTACTGGTAATCGTGAAAAAATTGTCATTACCAATGATCAAAATAGATTGACACCAGATGATATTGAAAGAATGATGAAAGATGCTGAGAAATTCGCTGATGAAGATAAAAAACTCAAAGAACGTGTTGAATCACGTATTGAGCTTGAATCATATGCTTACTCATTAAAAAATCAATTGAGTGACAAAGAAAAACTTGGTTCTAAAATAAGTGATGAAGATAAAGCAAAGATGGAAGAAGCTATTGATGAAAAAATTAAATGGCTCGAAGAAAATCAAGATACTGATTCTGAAGAATATAAAAAACAAAAGAAAGAACTTTCAGATATTGTTCAGCCAATTATTTCAAAACTTTACCAAAACAGTGGTGGTCCACCACCAACGGGAGGTGAAGAAGATGATCTCAAAGAAGAATTATAAATTATAGACGATTAAATAATTTTTTTTTTTTTTTTTTTTATATATATATATATATTTTCATATTTGTACGCAGTCGATCGGCCGCTCCTTACGCCACGTGATTTCGACTTTTCTTCCCAACCTTATTTTAGTCAACCCACCAAAAACCTCGTTATTATTCCTCCGTACAATAAATATTATTATTCACACAGTTACACTTCTCAAATTCCACCTGATTGCCAAAGAAGCTTTATATAACATACGATTATCGTTTTATTGTTTATTTGGATAGGACTTAAGTAATCATTTTACGTTTAAAGAAAACCCTAGGTATAAGTATCAATTTTTAATCACGATTTTTGTTATAATTTGTTTTCTTTTTGTTTTTACTTTTTTTTTGTACATATTATAATGAAAAACATCTCTTTTCCCCCA

>U34057

TTTTTATTTAATATAAAAAACATTTATTCAATAAATACATTTGGATGTGTTTTAAAATAAAAAACCATTAAAAAAATTATTTTAATTTTTCTTTTTATTTATTTATTATTACGATGAAATTATAGCAAAAATGATATTGACCTACGTATTATTTGGTATTTTATTTATATAAAAAAAGAAAAAGAAGAAAAAAAAAAAACATGAAGTCTAGGAATGATTTAATCAAGTCATGAAATGGATAAACTTTAACGTATGTTAAAATGAAGAAAAAAAAAAAAAAAACAAGATCACAATGTTAATGACCCAGTTAAAATTTGTTATTGTTGATATATTGTTGTGTAGTTGTTATTTTTTTCAGTCTAAAAGTGATTAAATTAATCAACTTCTTCCATACGTGATGCATCTTCACCATCAGCTTCAACAGCTGGAGCTTCATCAGCAACCTTTGGCTCTTCAGCAATTGGTTCATCATCATCAATACCAAGACCAAGTTTGACCATACGATAAATACGTGCAGCATGTACTTGTGGTTCTTCCAAACTAAAACCAGATGATAATAATGATGTTTCAAAAAGAAGAACAACTAAATCTTTAACTGATTTATCATTTTTATCAGCATCAGCTTTTTGTCTGAGTGTTTCAATGACTGGATGATCTGGATTAATTTCCAAATGTTTTTTAGCAGACATATAACCCATGGTTGATGTATCTCTGAGTGCTTGTGCTTTCATGATTCTTTCCATGTTAGCAGTCCAGCCATATTGTGATGTAACAATACAACATGGTGAATCTACAAGACGATTTGATACAACAACTTTTTCAACTTTGTTGTCAAGAATTGTTTTCATGGTTTTACAAAGTTCTTCAAATTTAACTTTGTCTTCTTCCATTTTTTTCTTTTCTTCTTCATCTTCTGGTAATTCAAGACCTTCTTTTGTAACAGATACAAGTTGTTTACCATCAAATTCTTTAAGTTGTTGTACAACATATTCATCAATTGGTTCAGTCATATAAACAACTTCAAAACCACGTTTCTTAACACGTTCAACAAATGAACTGTTTGCAACTTGATCTTTACTTTCACCAGTGATGTAGTAAATGTGTTTTTGATTTTCTTTCATACGACCAACATATTCTTTCAATGCACAACTTTCTTCACCAGATGATGATGTATGATATCTCAACATATCAGCCAATTTATTACGGTTGGTGCTATCTTCATGAATTCCCAATTTCAAATTTTTACTAAATTGTTCGTAGAATTTTTTGTAATTTTCTTTGTCTTCACTGAGTTCTTCAAAAAGTTCAAGACATTTTTTGACAAGATTTTTACGAATAACTTTAA

>U34058

GAGATATCGTACCAAGCGTGTTTTCTGTGTTTCAATCAAGTTTTAAACGTTTTTTTCTCTATTAGCATTATATCAATTACAATTGAAAATGCCTGAACAAATGGAGACTACCGGTGAAGTTGAGACCTTTGCCTTCCAGGCTGAGATCGCTCAATTGATGAGTTTGATCATCAACACCTTCTACTCAAACAAAGAAATTTTTCTTCGTGAATTGATTTCTAACTCAAGTGATGCTCTTGACAAAATTCGTTATGAATCACTCACTGATCCAACCAAACTTGACAGTGGCAAGGATCTTTCAATCAAGATTATTCCAAACAAAGAAGAAGGTACCTTGACCCTCATTGATACTGGTATTGGTATGA

>U34414

TAAAAAAAAAAAAAAAAAAAGAACAATTATTCATAACGATCAGCTGGTGAACGAACATCGTCAATTTTGAGAATCATTTTAACTAGCTGAGTAGCCAATAATATTTGTTGAGTTTTGCTCTTTAGTGATTCAATGACATTTTGAACTTTCATGTCACTTGTTCCACAATTCATACAATCAATACCAATTGCTGGATTTTTTGTTGCAAGTTGGGCTGCTTTTGCTTGTGCAAGACTATCAACTGAAGATAGACCAGCATTTTCAGCAAGGGCCATTGGAATTGCTTCAAGTGCATCAGAAAAGGCACGGAATGCATACTGTTCTAATGAGCTAATGCTATTTGCATGTGCTGAACATGCAAGTGCACATGATATTTCAGAAGCACCACCACCATAAACAATACGTTCATCAGTAACAAGATTTCTGACAGCACAAAGTGCATCATGAATTGAACGTTTTGCTTCAGCAACAATCATTTGATTACCACCACGAATAAATATTGTAACTGCTCTTGAATTTTTACATTCTTCGATGACTAACATGCGATCTTTGGTGGTACCAAATGTCAACTCTCTAACAATACCAGCATGTCCAAGTTTATCAGGTGTTAATTCTTCAAATCTTGGAACAATTCTTCCTCCAGTTGCAATGGCAATCAACTCAATTTCGGGTCCACCAACCCATCTGACTGCTGGTAATTCTTTTTGAAGTAAAAGATGATTAGCTTCATCATCAAAGCCCCACTGGCAAATAGCCAATGTTGCTCCAGCAGCTTTTACTCTGTCTACCATTTCGGTAAACTTTTCACGTTCGTATTCACGAAGTTTTTTGTAATCTTCAACTGATGTTACATCCAACTTATGTTTGGTTTTTGGCTTTGGTGGTTCAAATGGACAAGTAAGAATTGCCAATTTAACATCACGAAGTTTCTTGGGCATTTGAGGATGACTAAAATCTTTGTCAACGATAACACCACGAACTAGCATAGTGTCCTCCAAGCGACCACCAACTTTGCATTCAAGTTTGATTAACTCAAAGTTAACATCACGTTTTTCAAGATCAGCAACAGCCATAACTGCATTAACTGCAATTTCAGCCATTTGTCTGTGACATTTGTTGATGATTTTTGAGCCAAGAGTAGTCATGGCAGTTTTAATGAGTGGCTCAACATTGTTACGATCAATTGGAAAACCTTCAGCAATGCTATTAAGATGTTTGGTGGCACATTGTGCAGCCAATTCAAAACCATCAGCTATTCTAATAGGGTGAATGCCTTTGTCTAAGAGTCTTTCAGCTTGTTCCAACAAGGCACCAGCCAAAACAACGACACCAGTTGTACCATCACCAATTTCATCATCTTGAGATTGCGACAGTTGGACCATCAGTTTAGCAATTTCATGATCAACATCCATTTTCTGTAGTATTGTTGCTCCATCGTTGGTAACTGTGACATCACCATCTGGACTAACCATTAACTTATCAAGGCCTTTTGGTCCCAAAGATGTTCGGAGAGTGTTGGCGACACTTTTTGCAGCAGCGATGTGTGACTTAATTGCATCAGTGCCGGTTAGGCGTTTTTGATTTTCTTGATCCCGAAGTATAATAAACGGTCTTCCGTACTCGTCGCAATGAAATGATCCTGACATCGGATTCATAGTAAATTTTTTTGATTACAACAATCCGAAAAAAATGATTTAAAAGAAATTAATAAGACAGTTCAAACTATGCTTCCAAAGACTATTAGACCACCATCTTAGGTGTGTACGACAAGCGGCTGAAGCGACAAGGCACACAAGGGCTCGAGACTAGTGCCGAATGGTGAGACTGGTGAGTTCAGAGTTTCAGCAGAGAGAAGACTTCTCTCTGGTTTCAGTGGGTTTCA

>U34627

GTTTTTTTTAAAAAAAAAGTTAATTAAAATAATTGTGGCCAATGGATTTATTATATTTTATATACAATTGGCAATTTTTTTTTCTCTTTAAATTATGAACTATCATGAATTGCAAAACGAAATGATGATTTTTTGCCTGTTGATGATTCAGTTATGTGAATTATGAAATTTGATTCAGTTAAATCAGTATCAACTTTGAATTTTCCTGGTCCATTTATTTTCAATGAAATCAGTCCAATTTTTTTGCATTATTTTATTGTCATTATCTTCACCCTCAAATATTTTAATTTCAATTTTGTCATTTTTAATATTTGGTAATTCAATAATTGTTTGAATTTTAATTGGTACAACAGTATTTGCAGGTATAACAATTGATTGTGCATTATCAATTTTAACAGTAATTGTTTTAGCAAGTGAATGAAGTTCAACAGTTGTTGAATTTAATTCACAATCTTTAATACCACGTGATGTTAATTGTGATGATTGAATAGCAGCACCAACAGCCATTGTTTCATCATGACTCCATTTGCTTGTTAATATTTTACCATTTGGAAATAATGAGCCAATTTTTTCTTGTAATATTGGCAGTTTCATTGTACCACCACAAAGTATAATATTTTTAATATCAGCATGTTTTTTATTTGATGCATTTAATATATCATCAATTGGCTTAAGATAATCTGGTATATGTGGTCCAATAATATTTTCAAATCTTGCTCTTGTTATATTGTGACTAAAATCAACACCTTCACATAATGATTCAACAAAACAATGAGCTGTTGACATTGTTGATAATACATGTTTACATTTTTCAGCATCAAATTTAAGCTTTGATATTGATCTTTTACTTTCATTTGGATCAATTTTATATTTATTTCTAAATTCTTCAGCCAAATATATTGTCATTAAATTTGTTAGCTTTTGTCCACCTAATTGTGGATAATATTTTGATGATTCAATTGTAAATGCACCACCAGATATATTTACAAGTGATATATCACTTGATACACCACCAACTCTGTACACCAATACAAGTTCATTTTTAGTCTTATCTTGACCAAGACCATGAGCAATACAAGTTGCAATTGGTTCACCAATAACTTGTAGCACTTTAAAACCAGCTTTTTCAGCAGTACTACTCCAACCTTTGATACTTTCACTATTGAATCTATTTGGTACACATAATACAACATTATGTTCATCATCATCATGAGCAGCTGATATTGATATATCATACATTGCACCAAATAATAATGTTGATACTGAATCAACTGTAACTTTTTTAGTTTTTTCACCAACTTTTAATGCATAATGAAGTGCATTTTTATCATCATCAATAATTTTACTTGTACTTGTTTTAATTGCATCTTCCAATTCATCACTATCAATTTTTTTTATTCATTAAACGTTTATTATTTAAAACAGTATTTGGTCCATTTCTAAATATTCCAGCTTTTGCTGGTAATCCGGCTATCTGTTCAGATTCGTGAAATGAAATGGCAGCTGGTGTAACACGATCACCAGCATGATTTGCAACAACATCAACATTTTCATCTTTGTATATTGCCAAACATGCTGATGTATTTCCTAAATAAAGACCAAATGAAGCTGTCGCTGCCATGATTTAATTTTAATCTCAATTCTCT

>U36486

ACTCGGGGTGGTCCTGTTACCTTGGTCGTTGGCGATGATCTCCACTTTGCCGTGTTGCCAGACACCCACACATGAATATGTGGTGCCCAGATCGATACCAATAGCTGTACGTCCGGTCATCTCGTCAACTGTCGACTTTAGTCACGCACAATATTAAAACACGTAAATACGAGAGTGTAATATTTCGGCACCGCGATTCGATCGCAATAACGATTGAAGATTACTCTTTGACTTGTGTTAGAAAACACGCGCAGTAAAATAACAAATGTGTATCACACCGAAAAAAGTCGCACTTGCAATAGCAATATCGAAATGTCTCGTTGAATGAACCGCACTGTCG

>U39415

GCTGAGCAAGGAGGAGATCGAGCGCATGGTGAACGACGCCGAGAAGTTCCGCAGCGAAGACGAGCAGCAGAAGGAGCGCATCACCGCGAAGAACGCCCTGGAGTCTTACTGCTTCAACATGAAGAGCACGGTCGAGGACGAAAAGATGAAGGACAAGATCTCCGACGCCGAGAAGACCCAGATCCTGGACAAGTGCAACGAGACTGTCAAGTGGCTCGACTCGAACCAGCTGGCCGTG

>U42984

GTTAATGAAATATGGGGAAATGTACCCTCTATCAAATTTTAAGCCCTCGATGACATCCAATTCATCGTCTAAGGTTTTTCCATCTTTGACTGTTATCACGCCATCTTTTCCTACTTTTTCCATAGCAGAAGCAATAAGTTGTCCAATGCTAGTATCGCCATTAGCAGAAATTGTGGCAACTTGTGCAATCTCTTCTGCAGACTGAACTTTCTTTGATAATTCACCTAATTTTACTTTAACTTCGTCAACTGCTAACATAACTCCTCGTCTAATTTCA

>U7520

AATAAATAAGTTTTATTAATGGCTTTATTTATCATTTTGATCCTAATATAAATTATGAAGTATCTATCATTTATTTTTCTGCTGATTCTTGATTAAATGATTTACTACTTTGAATATTGATTGGTTAAGTGGATTTTGTTCCAAAAAATGTAAACTATCCCAATAGTGCTTTTTTTATTTTTGTGAAAATCCCATCATCAGCTGTTTGGGTTGATTGTGTTTGATCAGCTGATGGGCCATCTTTTTTATGATATAAGTCTTTTATTGTTCCAGGTGTCTTTTCTTCAAGCTCTGCATAAGCTCTGATCAATTCTTCTTGCTTCTTTGTTAATGACTTAGGAACATCTATTTTAATATTAACATAATGGTCACCAAGTAGATTGGAATTCACTCTTTTCATTCCTTGTTTGATTAATCTAATTTTGGTATGTGAACTAGTACCAGGAGAAATCTGTACTGTGTGATCATTGTAAATGCCTCTAACTCGAATAGATCCCCCTAATAATGCTTGAGATATAGAAATTTTAGCATCTGTATGAATATCAGAACCATCTCTTCTAAAATAATCAGATTTTTCAACTTTAAAAGTAACAAAAACTTCATTCCTATTGACCAAAATTCTAATAGTTTGTCCATCTTCAATTCCTGCTGGAACCGGTACTGTTACTTTTTTTGTGTTGTACAGTACTTCCTTTCCCGTGACATTCTATACATGGATTTTTTATTATAACTCTACTTCCTTGACACTGTCTGCATGTTGACTTCATTAAAAACGGACCCCTTGATATTGTTTCCATTCCAGTTCCATTACAATGTGTACAGGTTGTAGCTTTATATCCTAATTCGCAACGAGAACCACGGCACTTTGGACAAATATCAACCACATTTAAATTTAAATCTTTATTCACACCTCGAGCAGCTTGTTCAAATGTAATTTTAACAACAACTTCTTCGGCAGCTCCAAATCCAAATTTTGAATCAGCAAAATCTTCATCAAACGAACTATTATTTCCAAACCCACTTTGATTGAATATTTTCCTAAATAATTCTTCAGCATCTACATTAGACTGGTAACTCCATGCATTTTGAAAATTATCATTTCCTGTACCTCCTCCAGGTCCACCCATGTGGTTTGGATTACCAGTAGATCCCCATGTATCATATGTGCTTCTTTTTTTTTCATCACCCAATACTTCATAGGCTTCAGAAACTTCTTGAAATTTTTTACTAGCACTAGGATCTCCCTTGTTGGTATCTGGATGATATTTCTTAGCGAGTTGGTAATAAGCTTTCTTAATTTCTTTTTGAGAAGCGTTTTTTGGTACTCCTAATATATTATAAAAATCCTTTTTATTATTCAAATAACTTGTGGTGTGTATACTTTTATAACTGTAGAAATTCTGTGGTTTGACAAAGTGTTGAAATAAGCGTTTACTACATAATGTGTTATAACTGAGAGACGATGAAAATATGTGATGATGGTTTAATTTTTGGAAATTACACAACAAAGATGGAGAGTAACATTTATAAGAAATCAATTGTTTTATTTTCATACTGTTCATTGTGATAATATTTTAATTGTCTTGCTTCTGTGTTCCAGAAATACAATCAAGATGCATTAATAATGTCACAGATTGCTTAAATATATAGAATTAATAAAAATT

>U9923

GTTCATTTTAAACTTCAAAATTTTGCGTTAATGATTGAGAACTGGAAAATAAATCTTAATTCATTAAATTAATACTACGATCATTGTTCTATAGTTGTATTCATTTCAAAGCAGTCAGTTATAATATCTTATTCATAGTTTTTTCTGATCGGCGATTGTTACAAGCATCTCAATCTTTGTATACAGTATTTAATTAACTTTATATAGGTTCATAGAAGTATAAAATTATACAATATTATCATCCATAAACCATAACTTAATAATACCATTAATTTGAACCGCTATTTTTAATTTACCATTGACTATTTGATATATTAACATCAATAATATTATTTATTTTTTGATTATACTTAACTATGGAGAGCAACCGAGATGAGGCATCTCGCTGCATTGATATAGCTAGGTTATACTTTAAAAAAAAGGATTTGGATAATGCTTGGAAATTTTCAGTTAAAGCTAACAAAATGTATCCATCATCTGAAACAAAACGTTTAATATCAGAATTAAAATCATCAATGAAAAACTCAAACCAACGGTCAATGAATCAAAATCAATCTGATCATTCTGACTCATCTTATAATGGTGATGATTCCAATTCATCTTCCAGTACTGACTATAAAACTAAAAAACAAACAAAAAGATCAAGAAGAAGCCGCAGACCAAGTACTGTTTATTCACATAAGATGTTTAAAGAGATAAATAAAGATGAATCTTTTAAGTGTCTTGAAAGAGCAGAGCAATTCTTGAAAACTAAACAATTTGATTTAGCTGAAAAGTTTGTTCTCAAATCAAAAAAGCTTTTTCCAATACCTAGAGCTGATGAACTCTTACAAGAAATTCAAGAAATTAAAGGTTCCATTCATCCTGAATATACAGAAGAACAGGCAAATATTGTCGAAAAAGTGAAAAATAGTGAAAATCACTATACAATGTTAAACATTAAACGTACTGCAACTGTTCCTGAAATAAAAAAAGCATATAAGAAATTAGCTTTGTTATTACATCCAGATAAGAATTCTGCACCTGGTTCAGGAGAAGTTTTTAAAGATGTTAGTAATGCTGTTGACACATTATGTGATAATACAAAACGTCAAATATATGATCAAACTTTAATAAAACCTAAACCTTCTACTTCTAGAACTACAAATTATCCTCAAAGTTCCTGCCGATATCAATCTCCTTATTACCATACAAGAAAAAGAAATACTTATACTTCTTATACATTCTGTCGAAGTCCATCATTAAAAAGCTCTGACTTAGATGACGATGATTGTGATGTTTATGATGATTATGATGATTATATTAATGCCCACAATGAGTTCAATGAGTTCAATGGTTTTGATTATTATGAAATGTGAATATTATATTGATTTTGAGATTAATTGATAATTTATATTATAATAAATAATTGTACATATATTTTTTATCTTTGAAGCTTAA

>CL1364.Contig2

CGTAGTAGACCATACAAATATTATACTGTTTGGTTGTAAAATCATTCAGTCATTTAACCATACAATAGTATACGTTAATATTTAAACTACGTTAGTGTTATAATACATAATTATTTTATAAAAAAAAGTATAAAGATAATATTATTTAAAATTGAATTAAAAATTTCAATCTGTCTTTGAATTACGGTCAAAGAATGAAGACTAAAATTTGTAAGTTCTAAATATCAATGTAGACATATTATCACGACCGGAGACTGACGTAAGCATTAAATTCTCCAGGTGAAGGAGTTACTCCGTCAACACCTAAAGTGCTTGGTGGTGCTTGGCCTTCTGGTATCGAGAAATCAAAGTTTTGTAATAGACAAGCAGAAAATAAAAATATATTTGATCTAGCGAGTGTTTGTCCCATACAACGATGTTTACCGTATCCGAAAGGTAAGTATTCATCTGGAACAATTAATTTTCCATCGCAACCAATAAATCGTTCTGGCCAAAATCTATCTGGGTTGTCCCATACGTCATCGTCATTAAGTAATGCAGCAAAATTAGCAATCACCATTGTGTCCTTAGGAATATGATAACCCTGCAATGTGGTATCTTTCAGTGCTCTATGTGGGATGCTGAAAGTTCGTCCCATAAACACTCTAACCGACTCCAACACGAGAGCTTCTAGATAAGGCATACTAAAATACAGACGTTATGCGTGTTAATATATTAATGTTTACGAAGAACAAATTTTAACTAGCATAATATTAATTATAATTTTTAGAATATTTTTAAATCTAATTATGTATGTACTTAGGCCTATCATTTAAAGTGGGTAGTCTGTCTCGACCAACCACTCTATCGATTTCTTCTTGGGCCTTCTTCTGAACTTCTGGGTTTAGAAGTAGGTACAGGAAACCGAATCCTAAAGATTTACTGGTAGTCTCAGATCCAGCCATAAACATATCCATACAGATAGCTAAGAGCTGAGATTCTGAATAACTTTCTTTTTTGTCGTCTGAATGTAACATTTGTAAATACACATCCATCAAATCTCTTGGTTGGTTTAAAATAAATGTTTCCTTATGATCATCGAGTTCTGCTTTGAGAAATTTCCATACTTGTTGATGAATATTGACGAAAGATTTATATCCAGATGCTTCAGGAGCAATAAATCTCAAAAACGGGAATTGACTGAACAAAGCGCCTACCATATCAATATTGGCAAAAAGTTCAGTTAACAGAGCTTGTAGATTTTTCAATTCTATGTCATCTTCGTTGTATCGTTTGCTGGCCATCATTGACCATAATGTATTCAAGACTCCTACACTGAAAGCGTCTCTCATTTCAAACACTTCACCGATACCATTTTTAGACATGGCAATTTTTTTTTTGAAATCGTCGACTAACTGAACTGCTTCTTCTTGTACAAGTTCAGCCATTGTTCTTTTTCCAAAACCAAATTCTCGCAAATGACGCAATACAAATCTTCGTTGTTCTACCCAAAATTCTTCATCTGTTAGTAGAAGACCTGAAATAAAGATTACAATTTTACAAGTAATACATTTTTTAGACATAAAATGAATAATCTGTATAGTTACCTCTTCGTGTGCCCCATGTTCTAGTCTCATAGAATGGCCCCTGTGGTCGTCCATCAAATTCTTCTTTGGTTAACATTTCTTTAATTGCATTATATCCACAGCAAACCACTTGTCTATCTTTTCCTACTTTAAGACCAACAATTGGTCCATATGATTCCGCTAGACAAATAGTAGCACGATACAAGTACCCGGTTTGTTTCCTAAGCGAATTTACGGTAAGAGCACTTCCCAAAACTGGCAACCATTTTGGACCTGGTGGATAGTTTTTTGGTTTTCTCATGTCCAAGTATGACAAAAGCGCTACTACGACGCTGAATAGTACTAGCACGAGTATCCACATCCTGGTACTTGGAGTTCACTTGTTAACG

>CL1623.Contig1

CTTTTATACATTTTTCACAACAATATAAATAAATTAAAACTAAAAAAACAGATAAGAAGCCCACATCTCTTCCATTTTTGTTGTTACTATTTTTTTTTTTTTTAGTAAATGAATTATTTTTAAAATGTTCCTTAACAAAATTGTTTAACATTGTACATTTATATAAAAACAAAGTCTGTAACAGTATTATAAATCAGCTCCACACATCAGCAGAATATCTTTTCTGTTGTTCCATTTTTTTAACGTAATTGAGTGCTTGACTATTGGTCATTTGACCTTTTTCCATGACAACTTTTACAATAATATTGTGCACATCCTTTGCCATACTCCTCGCATCACCACAAACATATAAATGACCATTTTTTTCACCGATTATATTCCATAATTCGTCAGCATTTTGTTCTAACAAATGTGTTACATATATCTTTTCAGGTTGATCACGTGAAAATGCCAAATGTAGCTTTGTCAAGGTTCCATTAGCAACATATTCTTGTAGTTCATTTTCATAAATGAAATCTTCATCCTTCTTTCGACATCCAAAGTATAGAACCATTTCACCAATTTCTCTTCCTTCTTTACGTGCATAATCTCTTTCTTGTATAAAACCTCGGAATGGTGCTAGTCCTGTACCAGGACCAATCATTATAATTGGAGTTTGACTTTTAGGAGGCAATCTGAATTGAGATCTTCTAATATAAATAGGTATAGTAGGTTGTGGAAGTTCATCATTGGTTGGTTTCAGTTGGGCTAACAAGTTAGTGGCAACACCTTTATTGACACGATTAGTAGGAGTAGTATAATTTACTAGAACTGCTGTAATATGGATAGATTTTGGGTACACTTTTGGTGACGAGGATATAGAATAATAACGACACTGCAATCGTGGAAGTAGTTCACATAAATGGTCCAAATCGGGCTTAACACTCGGTAAATCTTCCAAAATATGAACAATATTACGGTTGTCACGTAAAATCCAATCGTGGAATTCTTTTTTTGCCCTCTGGGGTTGAGCTTGCCATAAGCTTTAATTTTTCTTGATCCTTTGGATCACTTGCATATTCTATTAATTCTTTCATAATATGTGTACGTGGATTTGAAGTTATATCCAAATAATAAGTTAAAGCTGTTCGATAAGTACAAGGACAAGGGAATGGATGTTTCTTACTCGATTCCTCATCAGTGTTCAACAGTGAAAATACAGTATCTAGATCGGCATTTAATAATTCTCCAATTTTTTCTACTAATTCAGAAGAATTTTTAGGATAGACAGCCACATGATCACCAGTATCATATCTCATTTTTGATCCATCAATATCAAACTCAATATGCATACAAGAGCGGTCGCCAGATTTGTGCAGCTCACGATTAACTGCAATTTTGGATAAATATGGGTTCTTAACATCAAATGGGAATCTTTGGTTTTCATAAGATTTAAGTCGTGATATTTCGCCCGAAAATATTCGTTCTGGTAAAACTTCGGAACAGTCAACTAACTTGTATTGTCTAATATTACTTTCTTCACCAGTTTCTTCAATACCATAATGGGAGCAAACACTTTCCCATAATTTTTCTTTCCAAGAAACAAAGTCATCTTCAATATTAGCATCATCGTCTCCCAAACCAATTTCATGAACTCTAGTAGCCCCTAATTCTTCCAAACGTTGATCAATGTAAATAGCAATTTCATTATAGTGTTCATAAGTTTTATTTCCCAATCCAAATACTGCATAATTTAATCCTTCTAGGTCAGCGTCACCATTTTGAAGCCATTCGTAGAAATCCATAGCATTATCAGTTGGGTCTCCCTCACCATATGTTGCTATACAAAAAATTGCCATTGACTTTTGTATTTCTTTCATTTCTACCAAGTCAGCCATATCACATTCTTCAGGATCGGCTACCATCCCTTTCATTTTATATCTAGCTCCTTCTTTGGCTATTCTTCCAGCAAATTCTTCTGCAGTTCCTGTTTGACTTCCATAAAATACTACTAAACTACGGCCGGTTGATTTAAGTTTTTTTATGAAGGAACTATTGGAGGTCTGTTCAATAGAACTCAATGAAGATGGTTGAATAGTGTAGGGTTTTTTTTCTGAAGAACTAGACTGTTTATCTCTTTTGAAGTACCAAAAATATGCTACTGTTATAATAACAACGAAAAGAGCAATATCTAAAGCACTAATCAATGGTTCTTCTGTAACAACAGTTGATTCGATTTTCACTCCTTCAGAATTCTCCATTTTAATTTTTAATTTAAAAAAATTGGCAAATTGTTATTGACTACCAAACGGCTGCGACTTTTTGTAAATCCAAAGAGAGATAACGGCGCGAGTACAGATGATATCGCCGACGGTACGTTACGAATTTACAATACTATTATTTTTATACTGTCAACACGATGATAATAATAATAAAAAAAAAT

>CL1721.Contig1

GAAATTGATAGTCAGAAAAACAAAAGATTACATCGTTAATTTGATCATTAGCAATGTGTTTTCCAACGTTGATGAAAGGAAATTATTTACTGTATTTAATGACGAGGATTTAGCTTAATCCATGCTCTTGGATTCTGTACAATTATCCCAGGTATTTTGTCATCCAGACTCATGGGTTCTCCATCGACAAAAGAAAAATCATAATGTTGAACAAGAGTGGCAAACATACCAAATACCATAAATCGTGCAAAAGTCTCACCAGCACAAAGACGTTTTCCATTTCCAAAAGGCATACTGTAGTCTTTTCCCAGCTGACCTTGATCATTTAAAAATCGTTCAGGTCGAAAATTTTCTGGATCACCCCAGGTATCTGGGTCATTGTGCATGGCAGTAAGATTTGTCATCATTATTGTGTCCACTGGTATGTCATAGCCAAATAATTTTGTATTTTGAATAGCTCTGTGTGCAACACTCAATGGTGTGATAGTTGCAATTCGCATGACTTCACGAATTGTTGCTTCAGTATAAATCAAATCTTTTCTATCATCCCATTTTGGAACACGTCCACGACCAACAACATGATCAATTTCAGCTTGTACTTTTTTCATGACTTCTGGATAATGCATCATGTATTTGATGCAATTGGTAATGACAGTTGGAACTGCACTCGCTGCAGGAAACATAAAGTCCTCAATAACCAATAGCATTTGATCTACAGAAAATCCAGCTTCATTGCTGCTTTTTAAATTTTTCATTTTACATAAATACGTTGACATAAATCCACGTGATTCTTTTTCATCTTCGGTAAGACTTTCTCCATGCTTCTCGAGATACACTCGAAAAAAATTAATCATTCCATTGTGAGCATTGTAAAAACTTTTCCAGCTAAAAATATCTCCAAAGTGTCGTAGCCAAGCAGTCATACTGGTAGCACCACCAGTTGTATCACCTCCAGCTTGAAATATAGATGCATCAATACAAAATTGACGAAGAATATCATGCTCTGATCTATCAAATCTTTCTCCTGACATAATTGACCACAACATGTTGACACATGTTGGATATAATATATTTGGAAAATATACTAAATCACCATTAACCACAGATTTTTCTGAATTATTAATTGGTCCATTGTCAATTGTATCAAGCATCACTGTTAATTCTTCAACGAAATTATTTTCCACTTCTTGATGACGACGACCAAAACCAAAATCTCTCATATTTCGTAATGCAAATCGTCGCTGTTCATGCCACTCAACAGCATCATTAAAAAATATTCCCAATGTTTTTCCAAATGCACGTATTTTCATAACATCACTAATTGCTCTTCCATCAAATTCTTTTCTATTAAGAACTTCTTTAATTCCAGCATAATCATTAACAATTACAACAAAAGCATTACCCAAATAACAGCTTATTATTTTTGATTTAAATTTTTTTACATAATAGGCTATTGTTTCGTGAGGAAATTTGTAATTGCCCCAAAGAAGAAACCAATATGATCCCCATAGTGGAAGTCTCAAAAGTCCTGGTGGAGTGTTATTTGGCTTGCCAGTGACAAAGGAATAACACATATATAATATTAATATTGTTATTATAAAAATAAATATAATTGACATGTTAAAGATGTGTTTTTTCTTGCAAAG

>CL1839.Contig1

ATCAATGTTAAAACCTTATTAGAATATCTTAAAGGAATTTCTGTTTTTGCACATGGAAAAACTTCAAATTTTATTAACATTTCAACAATAGCCAATTTCATTTCAATTCTTGCAAAACGTTTTCCTATACATATTCGAGGTCCATCACCAAATGGAAGATAAATACCACTTGGACGTTTAGCTTTCTCTTCAGGTAAAAATCTTTCTGGAATAAACTTTTCCGGGTCCGTATAATACTGTTTATCGTAATGCAATGCATAAACTGGAATTATTATTTTTTGTCCCTTTTCAATTTCTAATGAATCGTTGAGTACTTGATATGTTTTAGATGCTTTTCTGAACAAAGCAACCAGTGGAGGATACTTACGAAGTGTTTCAGCAATAACCATATCCAAATAATTAAGATCCATCAATAACGCGTTGTCAATTTGTCCGTCATTTTGGGACAGTTTTAGTTGAATTTCTTCACGAACTTTGTCTTGAATAGATTTATTCAATGCTAATTCATATAAACAAAAACTTACAGTAGTTGATACAGTTTCAAACCCAGCAACAAACATTACAAAAGCATTTGCTACAATTTGTGTTTCAGTAAACTTTTCATGTTTAGGTAAATTGGGGTTCAACACTAAATCTTTTCTTGCTTGCAGTAAATAATCGACGTAATCATTTCTGACTATTTTATTTTCTAGTCTATATGCTATTGTTTCTTTAAACACTCTGTGGAAAAATTCAGTCGCCTCCGTTGAAAAATCTTTCACTCTTATAACTCTCAAAAGTGAAGGGTTGATCATCAGACACAATTCTCTAAATAGCGCTCTTATTGAAGGTGTAAATATCGATTTTCCGTACTTACGAAATTTGGATTGATCATCGTTTATGGAGTTTAATTTGAGCCCGAAAG

>CL1839.Contig2

GGTTTGTGTTTATTGAACATAATATTATATTATACTTGGTTAATTAATTTTTTTAAATTTTAGCCAAATGCCATTTTTTGGTACTAATGTAAAAACATTATTAAAATAGTTTACAGGAATTACGGTTTTTTCACTTGGTAATACTTCAAATTTAGTTAATATTTCAACAATTGCTAATTTCATTTCCAACTCTGCGAAACGTTTTCCTATACACATACGAGGCCCATCACCAAATGGTAGGTAAACACCGCTTGGCCGTTTAGCTTTTTCTTCTGTGGAAAATCTTTCAGGAATAAACTTATTGGGATCCGGATAATACTTACTGTCAAAATGCAATGCATGTACAGGAATTATAATTTTTTGTCCATTTTCAATGATTAATGAATCGTTGGGCACTCGATATGACTGTATAGCTTTTCTGAACAAAGCGATTATTGGAGGATACATACGTAATGTTTCAGCTATGACCATATCCAAGTAATTAAGATCCATCAAAAGCTCGTAGTTGATTTGTCCGTTATTATTGGACAATTTTAAGTGAATCTCTTGGCGTAATTTGTCTTGAATAAATTTATTTAATGCTAATTCATATATACAATGACTTATAGTAGATGAAACTGTTTGAAATCCAGCTTCAAATATACCAATAGCATTCGCTATAATTTGTTCTTCAGTAAATTTTTCGTGTTCAGGCAAATTTGTATTCAAAACTAGATCGTTTCTAGCTTGTATAAAAAAATAAACCCAATCTTTTCCGTTTATTTTATTTTCTTCTCTATACAATACAGTTTCTTTAAAGACGTTATAAAAAAAGTCAGTTACCTCCTTTGGAAAATCCTTTAATTTTAAAGCTTTTACTAATACTGGGTTGATCATTAAACATACTTGTCCAAAAACCTTTTTTAATGAAGGTTTGAATATTTCTTTGCTGTATTTACGAAATTCGCATTTATCATCGTTTATGGTGTCCAACTTGAGTCCAAAAGCACAAATGCCGATTACGTTAGTCGAATATTTTTCTATAATATCCGCTACATCTACTTCATTATTATTTTTAATCAAATCATCATTA

>CL1839.Contig3

GACTTTGAATGCAAATTTACCCAAAGAAGAAAAATTTTCCGAATCACAAATTGTAGCAAATGCTTTTGTAATGTTTGCTGCTGGGTTCGAAACAACATCAACTACTTTAAGTTACATCTTATATGAATTAGCGTTGAATACGTCTATTCAGGACAAAGTACGTCAAGAGTTTCAGTTGAAATTATCCAATAGTGATGGACAAATTGACAACGAATTTTTGATGAGTCTTAATTACATGGATATGGTTATTGCGGAAACCCTCCGTAAGTATCCTCCTTTAATTGCTTTATTCAGAAAAGCATCACAAACATACCGTTTACCTGACAACCTAATACTGGAAAAAGGCCAAAAAATAGTAATTCCAATTTACTCACTCCATTTCGATGATAAATATTTCGAGGATCCTCAAAAATTCGATCCTGAAAGATTTTCACCCGAAAACAAAGATAAACGTCCTAATGGTGTTTATCTTCCATTTGGTGATGGACCTAGAATGTGTATAGGAAAACGTTTTGCTGAGATGGAAATGAGATTGGCTTTACTCGAAATGTTGAGCAAATTTGAAGTCCTACCATGTGAAAAAACAGAAGTTCCTCTAAAATATTCTAACAAAGTTTTAACATTGATGCCAAAACATGGAATTTGGCTAACATTTAAAAAAATTAATTAAATGAAGATAATATTACATGGAAT

>CL1839.Contig5

TTTTTACTTAATCGTATAGGAATTTCAGTTTTTTCACATGGTTCTACTTCAAATTTAGTCAGAATTTCAACTAAAGCCAATTTCATTTCCAACTCAGCAAAACGTTTTCCTATACAAATTCGAGGTCCATCGCCAAATGGAAGATAAGTACCACTTATACGTTTAGCTTTTTCTTCGGGTGAAAATCTTTCCGGATCAAATACTTCAGGATCCGTAAAATACTTTGGATCGTAATGCAGTGAATAAATAGGAATTATAATTTTTTGGTCCTTTTCAATAG

>CL2211.Contig1

GGATAACATCTGAGTGCTTCATTAATGACTGCATCAAGATAAACAAGTCCATTTACAGCTTCATATGTAACATCTTCATTTGTTTTTTCAAACATTTCATCAATTTCGTTTTGGAGTTTTTTTTGAACTTCAGGATTAGCAGCAATTTCATGTACAACGAATGACATGAGGCTTGATGATGATTCAAAGCCTCCAAAAAAGAAAATAAAAGCTTGTGATGTCATTTCTTGAATTGAAAGAGTTGTTCCTGTTTTATTACCTCTTGTCTCCATCATCAATTGAATCATATCTGGTCTAGTTATTCCTTTCTCATCTCTTGTTCGAATTGTATCTTCTACTATTTTTTTAAAAAA

>CL2699.Contig1

CGGAAACCGTCGCGCGTCGCGTCGTGATAAATTTATATTATAAACTTTGGTTTAGATTATTTCATCGAGTTTCATACATACAATGGATAAAATGTAAATTACTACGTATTAATAACTAAACAGGTCGCAATATAAATAATAATATGTCCGTGTTGGCCAAACAGTTATGGAATTTAAGGATTGCAACTTGTCGTGCTAAAAAGTCCACAGCAGTCCTTACAAGTATATCACAAGACGATGTAGAATTTGCCAAGGAATATTCGGAGTTACCAGGCCCTAAGTCGTTACCGTTGTTGGGCAATAATTGGAGATTCTTGTCATTTATCGGCGACTACAAAGTAACCGAAATAGACAAACTCTCATTGAGATTATGGAGAGAGTATGGAGACATTGTAAAAATCGAAAAATTGCTCGGGAGACCGGATATGGTATTTCTGTACGATGCGGACGAGATTGAAAAAGTATTTAGAAATGAAGAGCTTATGCCACACCGACCGTCAATGCCATCATTAAATTATTATAAGCACGTGTTACGGAAGGATTTTTTTGGTGATCTGGCCGGAGTTATAGCTGTACACGGTCAAAAATGGTATGAATTTCGAAGTAAAGTACAACAACCTATGCTACAACCGAGAACAGCAAAATTTTATATCGGGACTATTGAAGATACGGCAACAGCGTTTGTAAATAGAATAAAAAAGATAAAAAATAATCATCAAGAAGTTCCTGATGACTTTTTAAATGAAATTCACAAATGGTCGCTTGAATCTATCGCTAGAGTGGCGTTAGATCAAAAACTCGGATGCCTTGAAGATGAACACACAGTAGACTCAGATACACAAAATTTAATAGACGCCATCAATATATTTTTTGCAAATGTCCCAGAATTGGAGTTGAAAATACCATTTTGGAAATTATTTAGTACACCGACTTGGAGAAAGTATATAAAAGCGTTGGATACGATTACCAATGTTACATCCAAACATATAAATCGATCTATGGATCAATTATTATCTCAAAAATCATTTTGTCCAGATAACCAATCATCTTTGTTACAAAGAGTACTTAGTTTGGATCCATCTAATCCAAAATTAGCTCAAATACTGTCCTTAGACATGTTCATCGTTGGCATAGACACGACTTCAGCAGCTCTTGCATCAATTCTTTATCAGTTAAGTCGACATCCAGACAAACAAAAGAAATTAAGGGAAGAAATACGAACTGTGTTGCCCAATGCTGATTCAAAATTAACTTCCAACAAACTTGAACAGTTGCAGTATTTAAAAGCATGCATAAAAGAAACACTGAGAATGTATCCAGTGGTTATTGGGAACGGTCGCTGTATGAGCAAAGAAACAATAATTAGTGGATATAAAATTCCAAAAGGAGTACAAGTCATATTTCAACATTACGCTATTAGTAATAGCAGTAGATACTTTTCACACCCAGAACAGTTTTTACCAGAAAGGTGGTTAAAAGGAAGTGGATATAAACATCATGCGTTTGCTAGTTTACCATTCGGTTATGGAAAAAGAATGTGCTTGGGCCGCAGATTTGCCGATTTGGAACTTCAAACAGTTGTTTCAAAGATTTTTCAGAATTTTGAAGTCAAATACGAGTATGGTGATTTAGAATATACTGTACATCCAATATACATGCCAGATGGCCCATTGAAATTCAAAATGATTGAAGACTAAATATTTTTTAGAATTTGTATGCCAATGCTAAATACTAAATATTATAAGTATGTAATATTGTTTTAATATGTACCTTACGAGTATGAATAAGTAATAATTACACTTTAATTATGCTTCTATTAATTCATACAACCAAAATGTAAAAATGTAAATAATTTAAATATGTTTTTATGTTAAACTTAAATATAC

>CL3295.Contig3

TTTTTTTTAAATTTTGAATATGTATATATTTATATATATTATATAATTTATTAATATTAAGTTACATCAAGTAATTGTTTGGTATTTTACAAGACTACAAGGAACAGGTGAAATAAATACATAGGTCTGGTGCTTTTTATTCAGCTACTAATAAGTGTAGTTAAGTAAATATATAATTTGAGGGCGGACACAAGTCCCATAGGATACACATAATTGAGATTAGAAGCACCCACCCAGTTTTGATTAGGCCTTTTAGTTATAGTTTTATTAACATGAAATAAAAGGTGCTACTATCCATAATAATCTATTGGCAATTTTATACAATTATACACCATTTTCATGATAAATATTATAACTAAAATACATTAACTTGACATGAATATAATATATAAAAAATAAAATTACAAGTCAAAAATATTAATAAAAATAATAACACCTATTTTAGAATTGTATTTAACACACTTCTTTTATAAAACCATTTACAAACTACTTATTAGCTTAATTTTGTTTTTTTTCTTTGATAATATATACTATTATCTATCACAAATTGTTCATCCAGTATTCTTAAATTTTTTTATATGTAGGTCTTCTATCTCTCGGTTGAATAGTCACAGGATAACCATTAGCACTTCTCATTAATAAATCTAGTTTTAATTTAATATCACTTAATTTTATATCTGTGTGCACACTGTAGTTTCGCAAAAATGTTGATACTAAAACTTTCATTGACAGCATAGCATATTTGGATCCTAAACAACCTCTTGGACCACCACTGAACGCTATAAAACTATATTTATGGCGTTTATTAACATTTTCAGCGTCGAAATTATCCGGGTTATACGACCATGGATCTGGATACAGTTCAGGAATATGGTGCGTACATATTGGAGATATAATACATGTTGTTCCTTTTGGCAGCTTGTGATCACCAGAAAAAATTTTAACATCATCTTGGAGTTGTCTGAGTAACAACGGTCCTATAGGATATAATCGAAGCGTTTCCCGTATACATTGCTCGAGATAAACTAATTTAACAGTATCTTCAATAGTTATTGTTTGGTCACCATCGCCTAATACTTCAAAAATTTCATCGTAAACCTTGTCTTGAATTTCTGGATGGATAGCTAACATCAATAGACAAAAGCAGACTGTGATAGCACTAGTTTCACTACCACCAATCATCATTGTCACAACTTCATCTCTAAGTTCTTCATCAGAGAAATTTGCACCAGCTTCATTCAAATCTAATAAAGTGTCTAAAAACACTTTTAAACGCTTTTTATCATCATCGTTGACATCTATGGTGTTAATTTTATTTTCCATTTTTCTCTGTGCGTATGTTTCTTTCATTTCTTTGATCACCTGATTTGGAAGTTTGTGCAACGTTTTGTAGACTTTGTGTAGCCCCGTGAGTTTTCCATACAATGAAAATATAATATCGGGATATAACCATGGTTTGTAAATTCTCATGGAATCTAATTCTGAGGCTTTTGTTAATGCTTCAGCAAATTCAGACTCACTATTTGATTGAGTGTCAAGATTGTAACCCATTGCAGTTTGGCAGATTATATCCAGAGTGGTGTCCGCTATATAGTCCCAAAGGTCAAATGGTTTTGTTTTACCTAATTCCTTCTTTAAATTTCTGGTGAGAATTCGGTTTTTCTCGTTAAAAACGGGGAAAAACTGATCTAGAAGATTAGCGTTAAATACTGGAGTTATAAGACGACGATGTCTTCGCCATTTATGAACTGGGGCTGAAAACAAGCCTTCACCTACGGTATTCTTGAAAAATTTGTAGAACTGGTCTTTCTCCAGGGCCTTGGAACTGTTTAGTATTATTTGTACGTCTTCTGGCTTGCTTATGGTTACACCCAAAGATGTACCTAACCACATTTTGAACGGTTCAGAACCATAGATTTCGAACAGTTTAATGATTCTCTCCATGACCTGTTGGGGTGTACCAATAAACTCAAGCCCTGAGCCAATAATTGGGTATGACGGCGGTCCAGTCATTTTTGATGCTACTCTTTCGAAGTGTCTGCGGTTCCATTTGTAATGACACCATAACACCACAATAAAAGAAATTACTGCGTAAGCAGTCAACTCGGTACTTGTCATTCGTAGCCCACCCGCTGCATGTTTAGAAGTTGCGGGAAATTCTTGAGAAACTTCCATATTTACAACGTCGTAGGAAGGAAATCGTCTCGTCAATGTTCCAACCACAACTC

>CL3439.Contig2

TTTATATATATTTAGTGATACATTTTTATTTCACAAAAGTTGAAGATTGTTTAGAGAAAATTAAAAAAAAAAAACTAAAAAAAATAAATAATGTTTTGCGAAAAAAAAAATGTGTTTATCTATCGTGGAATATTAAATCTATGCAACTGTCAGGTATTCTGTTCATACGATAGATCATGCCAATATTTTTTGATTGTTCATTTGGCATACTGATTTTAAAATTCTTTATTAATTCAGACAGGAGTATTGTCATTTCTTGCATAGCAACGTGTTTTCCTGGACACATACGAGGCCCATGTCCAAAAGGTAACGAAGCGTAAGGATCAAACTTTTGGATGTTAGAATTTCTTAACCATCGACTGGGCTGATACTCTAAAGGAGCTTTAAAGTATTTTTCTTTTGTCGATGTTAAAGAATTGAAAGCCATCACCAATGTATTTGCTGGAATTTCATAGCCTCCAATGACAGTGGTTTTTGGAAGATATCTGGCATTTGCACCTGCAGTAGGAGACAGTCTAAGTGTTTCTTTTATGCAAGCCTTTAGGTATGCTTTTGTACCCTGTTGAACATCCTCTTGACAAGCTTTTTGTATGTTTTCATTTTGAGAAATATAATACAATGTCATTGCTAAAGTAGTAGCGGTAGCATCAATCCCGCCAAAAAATATTTCTAGTACAAGTAAATTGATATCAGTACTCGATAAATTTGTATTTGTATTAATAGCATTAACTAGAGCATTGTGTTCATGAGTCTGTTTCAGGTATTCGTCAATAAAATTTTTACATGTGGAATGTGAGCTTTCTAAATTTTTATAGGCATTTGTTTTATAAAATTGCCATACAGGCGGTTCTTGGAAAGTTTTGTACAATCCGTCCATGAATTTACTTCCTGCTTCTAAAAAACATTCTGACATGACTGTATTTAAACATTTTATTCTTAATCCAGGTGAGACAATAGAAATTGCTTCAATGGCAAATTTAGTTAAGTGATAAAACATATTTTTTAAACATTTGTCTTTATTGCGGTTCAATTTTATATATTCAATAAAATCCATTGCTGCTTCTTTATGCTGAGGAATGTAACTTTCATAAATATTTTTTTGCCATTAATGGAGCAATGGCTTTTCTCAATCTATACCATTCTACACCATTCTCAGATACTAATCCTCCAGTGCCAAATGGTCTTTTTCTGAGTAGGTTTAAAGCCGGAAATATGGGTCTCGCTGGGTACTTACCTTCATGGGCAAACATTGTTTTGGCGTCTTCCACTCTTGTAACAACAATCATTGTCTTCCCACCCAATATCAGTTTAAACACTGGACCCAACTTACATTCTATGTCACCCATAGCTTCTGTCAGGCGTAAAGAGTCGTAAGGACCGATTTTTGGGATGAACAAATGAGTGTGGCCCAGTAGAGGCCATCTTTTAGGCTCAGGTATACTACTAAACGGCTTGCACAGACGGTAAGTCATGACTAGTTATTGTCAGAGATAAACTGGGTACACAAAATCAGATTGGTCCTGTACGTTCAGTGGTATTCAGTTTAGTGATTGATTTATATTCTATATTTCAATAATATGAATAATATGATAATTCATATGTAGTCAGTTGAAGGCAC

>CL3440.Contig1

GTAGATCATATTATATATTCTCATTATTCTCATTATATTACTTTATTTCATATTCATTTGTTTTAACATATCATATTAGTATACGCTTTGTTATATTATAATATATTATATTATATTTTATATATCGATCCGATATATACGTATATATAAATATTTTAATATTTTTACCGTCCGTCGTACAAACGCTGCCCAATACACTATATCTCTTCGCCCGTTCGACCGTCTTGTTCACACCCGGTCCATCGGCTCTCCGCGCGGCGGAGGCGGCGCGAGATGACGGCCGAGATCATGTCCGAAGGCGGCGGATACAGCCGCGAACTGTGGCTGAACGCGGTCGCAGCGGCGCTGGGTCTGACCTACTCGGCGTACCAGCAGCTGCGGGCCGCCAGGACGCTGCCACCCGGGCCGTGGGGCGTACCGTTCCTGGGGTACGCGCCGTTTTTGTCCAACCACTGCACGTACCTCAAGTACAATGAGCTTGCCCGCCGGTACGGGCCCATATGCTCGTTCACGCAGCGCGGAAACACCGTCATACTGCTCAGCGACCACAAGCTCATCAAGACCGCGTTCGACATGAAGCAGATCACCGGTCGTCCCAACGACGGGTACATGGACATCATCGGAGGATACGGAGCGGTAAATAGCACTGGGAAGTTATGGGAGTCGCAAAGGAAGTTTTTGCATTTAGTACTTCGACATATGGGGATGACGTTCACGGGCCACAACAGGTTGAACATGGAAAACAGAATAATGATCGAAGTATCAACGTTAACGGAAACATTTCACAAGGCCTGTGGTAAACCAATCGATCTAAATGCCGGTTCGTTGTGCCTGGCCATCACCAACGTGATTAGTTCATTGACGATGAGTGTTCGATTCGAACCGAACGACCCGCGCTTCGAGCGGTACATGCACATGGTCGACGAAGGTTTCAAGCTGTTCGGCATGTTGAGGCCGGTGAGTTTGTTTTTGCCTAGACGTCATATTAACGACGAACGAAACATACAGGAAAAAATCAAGAATAACCATCGAGAAATCGCCTCATACTTTCAAAATATTATCGAAGAACACAGGAGTACATTCGATCCTAATTGCATTCGAGACCTGGTCGACGCTTATCTGCTGGAGATAAATCGCTCACAAGAAGCCGGCACGATGGACCAATTATTTCAGGGTTTAGATCCGAACAGGCAGGTTCAGCAAATTCTCGGTGACTTGTTCTCGGCCGGCATGGAGACTATTAAGAATACGATTTTATGGGCCATGGTGTACATGCTCCATTATCCGGATGTGATGACCAAGGTACAAGAAGAGATCGATTCGGTTGTGGGTCAATATAAATCGCCGGTGTTGGACGATTACCCCAATTTACCATATACACAGGCCACCTTATACGAAGTACTGCGGAAATCGAGTATCACGCCTTTGGGCACAACTCACGCAACCACCAGTGATGTAACACTCAATGGTTATCATATACCGACTGGCGCTCAGATCATACCTTTACAACACTTTGTACACAACGATCCAAACTTATGGGACGAACCGGAGGCATTCAAGCCGGAGAGATTTATAAATGCCGAAGGCAAAGTGAAAAAAACCCGATTGTTTTTTACCTTTCGGAGTTGGTCGGAGAAAATGTCTGGGTGAAACGTTAGCTCAAATGGAACTATATTTGTTCTTTTCAACCTTGTTACACGAATTCGATGTATGTCTACCAGATGGTGACGAATTGCCTAGCATGGACGGCCAAGTCGGTATCACACTAACTCCACAATCGTTCAAGGTCGTTATGAAAGCACGCAACAAATAGATTTTATCTAATTTTTTTATTCTCAGATCCTCTTATTTAGGTTGTTTATGTATTTTTAATGGAATATTATGTATTTATTAGAGTAATATTCCATAGTAATTGTTTAGTTTAAAAATGGTTTATTATTTATTTACCAATTGTACGTGGTTCCCTATAAATTATATACACATTTTCTAATATTTAATTTAAA

>CL3665.Contig1

GGAAAGCTCACTTGCGTAGACACCGATTAAATTTAATCGTCTTTACGTCAAATTAATATAAAATGCAATTAAAAAAATTAATTTGCAATTTTTTTTTTTTACAAATATAAACTCGTCATTTTGTGTTTCGTTTACAGTTTATTTTTATTTTATTTTGGCAACTAGGCAAGCTCGGTTGTTCACGGAAGGCATCGTATGGCAATTTATTTATTTATTTATTTATTTTAAAGAAAAAATTATATATAATTTATCCCATATAGATATATCCCATTAAGCCTTAGTAGCAAGTTTTCTTGGTTCAAGTGTAACTCTAAAACCTTCTGCTCTCTTAAGAATAATATCAGCTTGAAGTCTAAAATCAGCTTCTTTAATATCTGATTTAACACGGAAATTTCTCAATATTGTTGATAGTAATATTTTAAGTTTTAGCATTGCATATTTACGTCCTACACATGAACGTGGTCCAGCTGAAAATGGTACAAATGCATAGTAATGACGATTCGCTGTTTTTTTCTGGCAAAAAATTATCTGGATTAAAAACATCTGGATTTGGATAAATATACGGTTGACGATGTAATTTAATTGTTCCAATAACAACAGTGCAACCACCAGGTACAATATAATCACCTGATGCTAATTTAAGATCTGTTTTAATTTCACGTGCAATAATTGGTACGGGTGGATACATACGTAATGTTTCCATTAAACAGCGTTCAAGATATTTCATTTCAAGTGTATCTTGAAATGTTACTGGTCTATCTGAATCACCAAATATTTCATCCAACTCTTGGATTACTTTTTCTTGTATATCTGGATGACATCCCATCATTGATAAAAAGAAGCTTGATCCAGCAGCTGTTGTATCATGTCCTTCAAACATAATTGTATCAACTTGTTCTTTGACTTCTTGATCAGTCATAACAACACCATCCTGTCCTGCTTCAATAAGAAGATCTAAGAATGCAAGCCGTTTTTTCTCACCAACATCATTGTCATCAACATCAAGATCATCTTTCAAGCCAGATGATTGACCAAATGATAAACCATCAACACTTGTTACTTTTCCTTCTTTTTCATTGGTCTTTATGAAATTACGCTTACCAGCTTTGTAATCTTCTTTCTTACGTTTAATGACCTTCTTTGTTAAACCGTGAATGATTTCAAGCAATTGAATTTGATCTTTACCATATTTTGTCAAATTAAACAACCAATCGGGTCTTAACCAAACACGAGTATGACGTAAATGAAGAATATCACACATTTTCATAACAGCCATTGCATACTCAAGACCACTACGATCTTGGGTTGTTTTTGAAACACCCATTGCTGTCTCAAGTAGTATTTCAACAGTCGTTTCAGACATGTAATCATGACAATCAAATTCACCAGTTTCTTTGCGTAATCGTTCAACAACTGATCTTGAATTTGCATTGAATAAATCAATGAAACTTTTAAGTACATTTAAATGAAAAGTTGGAGCGATAAGTTTACGATGAGCCCGCCATTTTTGACCAGTTGAAATGAGCAGACCATTGCCCAACCATGGTTGGAAAAATCTGTACTCTGCTGATTTGTCAATGTAAACATGACTGGAGAGAATTACCTCAACATCACGTGGATCAACGAGAAAGACAACAAGTTTTGGACCAATCCAGAGACGAACAACTTGATCAAATTCGAATGAACGTTGGTAAATCTTACTGAAAATGGCATCAGAACTTCCAATTAACTCCAACGCATTTCCAACAAGTGGCCATCCAGCAGGACCTGGTAATTTTTCACCAAGTTCAATCAAATGACGTCGTGATAATCTGAAGTAAACGGCCCACAAAATAAGTGCAGGTATTAAAAGTGTTATAAAAACAGAAGATGCTGTCATTCCAGTTGCTGACGCTGCAGCAACTGTGCTAGCAACCATTTCTGGTGTAGCTGTTGACATGATAATTTAAATATACTTTATTCTTTTTCTAAAAGTAAAATAAAAAATTAAATTAATTTGAATACAATAGCTG

>CL4051.Contig2

GTCATATATTAACAGGGAATGTTTAAAATACAACTATTTTTTAACGGTCTGAATATTCCTCAATAAAACAATATTTATTTAGTGTGTTATAGTATAGTATAATTTTTGACCCACCACACTTTTTTTATTTTAAAGTTTTTAGGCTCAAGTTCTATATGTACTTAATATTTTAATTTCAATAAATGTATAGTTAATTAGCAGTATTTATTGGTTTGAATTTTAACCAAATGCCATTTTCTGGTGCTATTAATAAAGATTTTTGACTGAATCTCATTGGAATATCGGTTTTATCACATGGTTCCACTTCATATTTGGTCAAAAGTTCAGTTAACGCTAATTTCATTTCCATTTCAGCGAATCGTTTTCCTATACATAAGCGAGGTCCATCGCCAAATGGCATATAAGTTCCATTTGGTCTTTTAGCTTTTTCTTCAGGTGAGAACCGTTCAGGATCGAAAGTTTCTGGATCAGGATAATATCTATAGTCGTGATGAATAGCATACGCAGGAATAAGAACTTTTGTTCCTTTTTCAATTACTAAAGTGTCATCGGGTACTTGGTAATCCTTCGTTGCTTCTCTAATTAGTGTTATTAATGGTGGATACTTTCGTAATGTTTCAGCTAATACCATTTCCAAGTAATGTAATTCTTTTAGAAATTCATCGTTTATTTCACCACTATGTTTAATCTTTGTCGCATTCATTTCTTCTCGTACTTTGTCTTGTATTGGTTTTTTCAATGCTAGTTCGTACAAGCAAAAACTCATCGCCGTAGACACTGTTTCAAAACCAGCAGCAAATAGAGCAAATGCATTGGCCACGATATCCGTCTCTAAGAATGTCACTGATGGTTCGGTCTTGTTGACTACGAGGTCGGTCCTGGCTTGGATCAGCGACTGCATGAGGTCGTCCCTGACCAGGCCGTGCTCCTGCCGGTACCGCATCGTATCGGTGAACGCGGCCATGAAGAACTGGACGACTTCCTGCGGGTAGTCGCTGACACGGATCGCACGCCGAAGGGCCGGCGAGATCAGCCAGATAAGGTCCTTGATCAGCATGCGGAACGAAGGCTGGAACACGGCCTTGCCGTTCCGGCGGAACTCGGACTGTTCGTCGCTGATGGCGTTCAGCTGCAGTCCGAACGCGCACGTGCCGATCACGTCGGTGGAATACTTTCCGAGCACGTCGCGCACTTCTATCGGCCCGCCCGTGGCCGGCACGTCCGCGGCCACGTTGCGCATCAGCTGCTCGCTGCAGGCGGCCACCTGGCCACGCATGTGCCTCAGCTTGCCGGCCGTGAACACGGGACTGAGCTTTTGCCGCATCACCTTCCACTTGGCGCCCCTCATTTTGAACAGCCCGTTGGCCAGCGGGTTCTCTTCCGGGCCATCCGTGTGTATCCCGTGATCGGTGAAGTGAGCAAAGTCGCCGATCAGCACGCGGTTCACCAGCGTCCGGTCGCACACCAACAGGTATGGCGTACGCATTTGGTACATGCCGACGTACGGGTGACCGCGAAACGCGTCGTAGATCCGGCCGAACATCCGGATCTGGTGTTCGGTGCCCAGCGCCCTGCTGCCTATGTTGCCGAACAGCGGCACCGTGGGCCGCACGTACGGCACGCCCCGGTCGCGCCAGTAGCCGAACGTGGCCGTGGACCAGTGATATGCGAGGGCCGTGAACGTCGCCAGGAACCCGACCGTCCACCAGCTGGTCGCCAGCTCGAACAGTAGCTGCAGCGCGGCGGACACGGTCGTCATCGCGAGGCGATCCGACGAGTTACAGGGAAGGTAGTCGGGACGATGAGAATTTCAGTGATTTTTTATTTTTTTTTTAAAGTCGTGGTCTTGATACGAACGCTCGGCCACTGCGCTCCGACCGTACCGCAACAGGATAATAAAATACGGTCTTCACGAGCGGCTGCACATGGCTATTGTGTCGTTGTTTTTGAAGTTATTATTGTTCCAAACGGATTTTTTCGATGTCCTCCTGCGTCGCGTCCCTGTCGCCGCGGCGGAATCGCGTGCCACTCGCGATGCGATGATTAGTGACGTCGATAAAACTTTAATATGAATCAAACACAAGTATACCTATATACTTATGATCGACGTATACGAGTCGAC

>CL4213.Contig1

GAATTATTTCCTTCAATTTATGAATTTTTGGGTTCAATTGTTGATGATACTGAAGTTACAAATTTTTTTATCAACACAATGAGAGATACAATGAATTATAGACGTAAAAATAATTTAACACGTAATGATTTTCTTCAGTATCTCATGGAAATTGAAGATAATCCAGATAAATTACCAGAAATTAAATTAACTGATAGTTTTTTGGCATCACAATTGTTTATATTTTTTTTGGCTGGATTTGAGACATCATCTTCAACAATAAGCCATGCTCTTTATGAATTAGCACAGTATGAAAATTATCAAGATCAATTGAGACAAGAAATCAAAGAAACAAAAGAAGCAAATGGTGGAAAATGGACTTATGATTCTGTTAAAAATATGGTGTTTATGGAAAAAGTTTTTAGAGAAACTCTACGTAAATTTTCTCCAATTCCAATGTTGATTCGTGGATCCAATACTGAGTATACATTGTCTGGAACAAATGTAACAATTCCAAAAAATTCAAAAATTGCATTTCCAGTTTATGGAATACATTATGATCCAAAAATTTATCCAAATCCAGAAGTTTTTAATCCAGAAAATTTTAGTCCAGAAGCTATTCAGTCTCGACATCCAATGAGTTTTCTCAGCTTTGGAGAAGGACCAAGAAACTGTGTTGGAATGAGATTCGCTTATCAACAATCAAAAGTTGGGCTGATTAAAATTCTAGAACGTTATCGTGTTGATGTTTGTGATAAAACAGATATACCATATAAATTTAGTACTCAAGGTTTTTCCTTGGAGCCATTAAATGGAATTTACCTGAAGTTTACTAAACTTGATAATTCATCATCAAAATGAATGTTATAAATTTTTGTTTTTCAATCAAATGATATTTAAATCAACACTTTTAATAACAAT

>CL4499.Contig1

CTCATTAGTTTTAGTAAATTTTACTAAAACAAGATTTATGTCTACATCAAATTTAAAAAATATAAGAACTAAATCAAAAAAAGAAATACCTATTGTTAAGGGTTTACCAATAGTTGGTACAATGTTTTCAATTTTGGCTGCTGGTGGTGGTCGTAAATTACATGAATATATTGATAAACGTCATGAACAATATGGTTCAGTATTTAGAGAAAAACTTGGTCCTGTTGATGCAATTTGGATTAGCAACCCGTTAGACATGAAATTATTATTTGCACAAGAAGGAAAATTTCCTAAACATATTTTACCTGAAGCATGGTTACTTTACAATGACACATATGGCCAACAAAGAGGACTCTATTTCATGGATGGAAAAGAATGGTGGAAATATAGACAAATATTTAATAAAATTATGTTAAAAGATTTAAATGTACATTTTATTGAATCTTATAAAATTGTAATTAATGATTTATTAAACGAATGGGAATTGAAAGATGGACAAGTGCTTCCAAATCTTATAGAAGATTTATACAAACTTTCAATATCATTTATGGTGGCTCATTTAGTTGGAGGAGTTTATGATAATTATAAAAATGACATATCAAATGATGTAAATTGTTTAGCACAAAGTATACAAAAAGTATTTCAGTGTACTGTAAAATTTACAGTTATTCCTGCAAACATTTCTAAATCATTAAAACTGAAAATTTGGAATGATTTTGTACTTGCTGTTGATAATTCTATTGAAAGTGCAAATAATTTAGTTTCAAAATTAATGAGCTTTAATGGTGATGGTTTATTAAATTCTATACTAAATGTTCATGACATACCTATTGATATGGTTAAGAGGCTGATAGTTGATTTTATAATTGCAGCTGGTGATACTACTGCTTATTCAACTCAATGGTCTTTATATACTCTAGGGCTTCACAAGAGTATACAAAATAATTTAAGACATAGCTTATTAGAAACAGATTTTTTGGAATGTGATTATTTAAATAATGTTTTAAAAGAAGTTTTAAGAATGTATCCGTTGGCACCTTTTCTTGCAAGATTTTCTCCAAACGATATATATTTAACAGATCACATGATACCTGCAAATAGTTTAGTTGTTATGTCAATGTTTACAAGTGGTAGAAATGGAAAATATTTCAATAACCCTGATGATTTTGAACCAGATCGCTGGAATCGTCTAAAAAATAATAAATATAAAGGTGTAAGTGAGCCATTTGCAACTCTTCCTTATGGATTTGGTGCAAGATCATGCATTGGACAAAAAATGGCACATGTTCAGATGTGTTTGACACTAGCAGAGTGCATCAAAAGATATGAAATACAGACAATGAAACCAGTGAAAATTGCATTAGATTTAATTACAGTTCCAGATAATCACATAAATATAAAACTAAAAAAGATATAATTTTTAAGTTATTAAATTATTTATCTTTATACCTAATTAAATAGGTATTGGCTCATAAAAACAAAGCCGTAACTTAGGGTGAAAGGTCCATGGATCCAGACCCAACGCCCCCTCGAAATTTTTAAAAAGTAGAAATATATAATGTAAATTATAGATAAAAATGACAAG

>CL4758.Contig1

AACGAAAGTCACATGAATATAATTTAACTTGACATGAACAAATTGAATTAATAAAATATAAATTCATCATGATATTAGAATTAACAAGTGGTATTTTTGTTTTGGGTGTTTTAATTTATTGTTGGTCAATTTCAACATATAATTTTTGGCATGAACGTGGTGTACCAGGTCCTAAACCTGTACCACTTCTTGGTAATATAAGTGATCTAATAACTGGAAAAAAATCAGTTGGCCAATTGGTCCAAGATTATTATAATGATTATAAAAATGAAAAAATGGTTGGTTTATTTTTTCGTCGTGAACCAATACTGTTGTTGAGAGATTTGGATCTTATTAAAGATATTTTTATAAGTGATTTTTCAAAATTTTCTGATAGACCAATGAGAGTATTTGAAAAAGCTGAGCCTTTATCACAGACATTACTACATCTTGAGTATGCAAGATGGAGACCATTGAGAAATAAATTATCACCAGTATTTACATCTGGAAAATTAAAAGATATGTTTAATTTAATACTTAATTGTGGTGATAATTTACAAACTGTTATGGATAAAATTATTGATAAAAATAATATTATTGAAGCACGTGATTTAACAGCTAGATTTACAATTGATGTTATTGGTGTTTGTGCATTTGGTCTAGAAATTAATGCAATGAATGATGATGATAATAAATTTCGTAAAATGGGTAAACGAGTATTTCATATGGGTTTTAAAAAAATATTATTATTTCAATTGAGAGAATTATTTCCTTCAATCTATGAA

>CL4758.Contig2

TGGTATTTTAATGTTATTAATACTTGGCTATTGGTGGTCAATAAAAACATTTGAATTTTGGAGTAAACGTGGTGTTGTAGGACCTCAACCTGTGCCACTTTTTGGAAATATTATTGATACAATAATTGGAAAAAAATCAATTGGTCAATTGATGCAAGAATTTTATAAAAATTATGATAATGAAAAAGTTGTTGGTTTATTTGTTTATCGTGAGCCAGTATTGATGTTGAGAGATTTGGATTTAATAAAAGATGTATTTATAAGTGATTTTTCAAAATTTTCAAATAGACCAATTAAAATATTTGAAAAAGCTGAGCCATTATCTCAATCATTATTACATCTTGGCTATAATAAATGGAAA

>CL533.Contig1

TTCTGATGTGTATAAAATTAGGAACGTATAATCATTATACTAATAGTTCGCATGAACACTACAGTCTGATAGTTGTAATTTAAAGAATCCAATGAAATAATATTTATATTTTAACAATGATTTCTATTATTTTACATTATTTGTTTGATAATTTCACCTTAATAAGTACTTCCGTGACTGTTTTACTTTTGTATTATTTTTCAACTTCCACGTACGACAAATGGCAAAAAGCAAATGTACCTCATATAAAACCTATTCCGTTATTTGGAAATTTAGCGAAAACACTTCTCGGTCTTGAAAGCTTATACGAAGGGTTCGATAGAATTTACAGACAGTTTCCGAATGAAAAAATTTGTGGGTTTTACCAGATGAAAACACCATACCTTATGATTCGAGATCCAGAATTAATCAATACAATTCTCATCAAAGACTTTACGCACTTCACTGACCATGGTTTCTACTTGGATCCGTCGATCAATCTTTTATCAAAAAGTTTATTTTTCATGAACGGCCAAAGATGGAAAATTATGCGACAAAAGCTCAGCCCTGGATTCACGTCCGGTAAACTCAAATTGATGTATGGCCAAATCAAAGACTGTAGCAAAGAGCTATTAAACTATATCAGTAAAAAATCTAGTACGTCCGATGAAATTGAAATACACGATTTATTAGGAAAATACACAACCGATGTCGTTGGTACGTGTGCTTTCGGTTTAAAATTAGATAGTATGACTGATGAAGACAATGAATTTCGGAAGCATGGGAAAAAATTATTCAAACCAACATTAAGGCAGCTTATCATCAACATGTTGGGAATGATTTCACCGACAATTCCAAATAAATTGAAAATTCAACAATTTTTACCTGAAGTAATCGATTTCTTTGATTCAACATTTAAAGAAGTCATTACGTATAGGGAAAAAAATAACATAAACAGGAACGATGTGGCACAAACATTGATGCAAGCCAGAAACGAACTTGTATTGAATAACGATTCATTCCCTGAAGAAAAATTTACAGATACAGATATTGTTGCAAATGCGATTCTTCTGTTTGTCGCCGGTGCTGAACCTGTAGCCGATACGCTAGCTTTTTGTTTTTATGAATTAGCGTTGAATAAACCAATTCAAGAAAAACTGCGTCAGCATATTTGTGAGACAAGAGAAAAGCATGGTGGAGAGTTCAATCATAGTTACTTAGCGAATCTACATTACGCCGACATGGTTTTATTGGAAACAATGCGCAAACACAGTGGAGTTATTGTTCTGTTTAGAGAAGCAACTAAGACTTACCCAGTCCCTGGCCAATCATTGGTTATTGAAAAAGGACAAAAGATAATAATACCGACTTACAGCTTTCATCACGATCCCAAATATTATCCTGATCCAGACATTTTCGATCCAGAAAGATTTTCTCCTGAGGAAAAAGCAAAACGGCTTAATGCTACTGAATTATTTTTCGGCGATGGACCTCGTTTTTGTATAGGAAAACGTTTGGCTGAACTAGAATTAAAATTAGGTTTATCAGAAATAATTTATAACTTTGAACTTTTACCGTGCAGCAAAACTGAAAACCCAATACAACTTATACCAAAAGGAATGATTGTAAAACCAAAGAATGGCGTTTGGTTGACTCTGAAACCAATCATTAGACAATAGGTCTACTAATGATGATTATTGATTATATTTAATCTACCTTTGTAATGTATTATAATAGTATATATTTATATTTTTAATTTTAATAAATGAAAAGTGATGTTTTATGAAGACAATTAACGGAAAACTATAATTAAAATAGTATTAAATGTCAAATTAG

>CL554.Contig3

TCACAGTTATCTTTTTTCATAAAATCAATCAAATATAAATAAAATGGTATACATGTTATTATTTTAAATAATTAATATATAACCAATTATAATTTATTATCCACGTAGATAATGGTTTATCTAATTTTATTTAAGTTTAAAAAACTTCATCTTTGGAATTGATTCTATTCTCAAGATTAAATGCGTGATAACTTGAATGTCGTCTTTGCTCCCCAATGATTCCAATTTCATGTTCCGTAATATGGTTGATATAACAGTTTTCATTTGATTCATTGCATACTTTTGCCCAATACAATTTCTGGCACCAGCGCTAAATGGTATGTATCCAAAATGAAATTTTTCCTTATTTTTCTCTTCCAAAAACCTTTCAGGCTTAAATTCTTCAGCATTTGAATATATGTCTTCATTGCGGTGTAGAACATAGGGATATACTATTACTACTGTCTTTGGAGGAATAGTATAATTATTAATCGATAATGGCGTTTCTAGTACTCTACTAAAGCCCGGGACACTTGGATAAAGTCGAAGAGATTCTTTAATGATGGCTTCTAAATATTGCATGGAATTTAAGTCTTCCATCGTAGCATCTCTGTCAGAGTCTCCAAAAATGTCAAATAGCTCGTCCCTAGCTCGATCTTGAATATCCTGATATATCCCCAGCAAAACTAAAGTCATTGTCATTGATATAGAAGATGTGTCGTGACCTTCAAAAAGAAACGTATCTACTTCTTCACGAATATCTTTATCGTTCATCTGATCCGGGTTGTCATTCAAGACGTTAAGAAGTAAGTCTAAGAATGATTTCTTTTGCGGTTTTTCAAATGTACTGTCGGACTGCACTTTTTCAATAATTGTTCTATTCAATAAAGCTCGTTTATTTTTAATCACGTTATCAGTAAACTCATGTAGCACATTGAGTGATTCAAAAAATCTTTGACCACTTTTGCTAATTCTATAAATAAAATCATTCCATAGCCAAACTTTGAAAACACGCTCAATCACTGATTTGCAAGCACATTTGATTGAATGAACATATTCAACTGATTTACCTTCTTGAGCATTCATCTCGGTACCCATTATTGTTTCACACATCATATCCAAAGAACACAAAGTGATGTAGTCAAAGATAGAGATTTCTTGATTGCCATTCTCAGACGCATTCAATAGCTTGTCAACCAATTTACGCGAATGTTTGTTAATTGAAATATTGTACATGCCTAATGTTTTGAAGTGAAACGTATTGGTAAGGAGCTTCCGCCGGTTTTGCCATTTTTGACCTGAACTCAACAAAAGACCGTCGTTCAGCCAAGGTCTCAACATGTCATACTCTGGACCTTTCTTGATATATTGGGTACTGGACAGTAGAATTTTTAAATCATCTTTATTGTTTATCAAAACGTATGATCGACTTGAAAAGTTAAAATGAACTACTGAACCGTAAATTTTTGTCAACTCCAAAAGAAATGGCAGTATATCCTTGGGCTGAAACATTGCCATCTTCCACGATAATTGTATCTCACTCCAAAAGGTTTTCGTAGCGGATGGTACGTGAGACGAGATCTGTCGGTATTCCAACGGCATGCGGAGCCGCGACCATATCGCATACGATATCAAACCCACCAAAAATATCAACACTACACCGTAAAGGTTATGTAGTTCGATCATTTCCGTCACGTCTGCGGTCCGTCAAAATAAATCTTCTTTTTACCTCGTAGCCAAAAGACAATAATTAAATGTTTATTAATTTATTATAAATTACGACGATAGGTATTGTTTTCGTTATCCGTTAACGTATCATACGACAGTCCAGCGAGTGTACCGTCGAACAACTGAG

>CL554.Contig6

CGCCGTCGTCCTCTCCGATTTTCGCGTAAAAACCGTGGCTCGCAAGGCCCGCGAAGTTTGACGCCAGTCCGGAGACGAAAATACGATTTACCATGTTGGAACCCAACCTTGTCAACGTCGTGCTTATCCTGACGGCGATTTTGATATCGTGTGCGATATGGTCGCGACTCCGGAAGCCGCTGGAATACCGGCAGATATCGTCGCATGTGCCGTCCCTGACTAAAAACCTCTGGGACGAGATGCTGTTTTCGTGCACCATGGGGATGAAACACCCTAAAGATTTATTGCCGTTCTTCAAAGAAATATTTGAAAAAAACGGCCCAGTAGTTCATGCCAATATCACTGGACGGTCATATGTGTTACTAAATGATCCGGATGACATAAAAACTCTGTTATCCAGTACTGCGTACATAAACAAGGGTCCAGAGTATGAAATGTTGAAACCGTGGCTGAACGAGGGACTTTTATTAAGCAAAGGTTTAAAATGGCATAACCGTCGAAAGCTCCTGACCAACACTTTTCACTTTAAAACATTAGACATGTACAATCCGGCCGTCAATAAGCATGCAAGAGTATTTGCGAAAAACCTCTTGGACGCGTGTGCGGACAATAAAGAAATTTCAATTTCGGAATACGTGACGTTATGTTCTTTAGACATAATCTGTGAAACAATTATGGGTACTGAAATGAATGCACAAAAAGGGAAATCAGTTCAATACGTTCATTCCATTAAAAGTGCTTGCAGGTCAGTGATTGATCGAGTTTTCAAATTTTGGCTTTGGAATGATCTAATTTATAGAATCAGCAATAGTGGTCGATCGTTTTTTACATCAATCAAAGTGTTGCATGAATTTACAGATAATGTCATCAAACGTAAACAATCGTTGTTAAAAAACTCTGAAAATCAAAAAGTGCAGCCTGAAATCAAACCTGAAAAAAATCGACCAAAATCATTCTTGGATTTACTTCTTGACGTTTTGAACGAAAATCCAGATCAAATGACTATCAAAGACATTCGAGAAGAAGTAGACACGTTTCTTTTTGAAGGACACGACACATCTTCCATAGCGATGACGATGACAATTTTATTGTTGGGGTTGCACCAAGATATTCAAGATCACGCTAGGGAAGAGCTATATAGTATATTTGGCGACTCCGACAGAGACGCTACAATGGATGATTTAAATGCCATGAGATATTTAGACGCAGTCATTAAAGAAACACTCCGACTATATCCGAGTGTACCAAGTTTTACGAGGGAATTGAATACAACTTTACAACTCAAGAACTATACCATACCTCCAATGACAACAATGGCTATATTTCCCTACGTTTTACATCGAAATGAAGACATTTATCCAAAACCTGAAGAATTTATTCCTGAAAGGTTTTTAGACGAAGAAAATAAATCAAAATTTCTATTTAGATACATACCGTTCAGCGCAGGCGCAAGAAATTGCATTGGACAAAAGTATGCTATGAATCAAATGAAAACAGTAATATCGACAGTATTAAGAAATGCAAGGATTGTATCTTCGGGAAGCAAAGAAGATATTAAAATCAGTATGCAATTACTAATCAGAATAGAGTCACTTCCAAAAGTAACATTTCATAAACTATAGCTAATTATATTTTATACAGTATTATATAGTTAATAGTAATTTGACGATATTTGTTTTTTGTGATGAATGTAACGGTCATAGAAAGTTTTTGTAAACCTAAATATATTCAATACAGAATACAGAAACATAATAATTTAAGACCTTCGTCTAAAAATTACCGAGGATTAAATTATTTGTTTAGTTTTTAAGTATTTGATATTTAAT

>CL5992.Contig1

GAAATGACACAACAGCATCAGTAAATTGTTTTGTTATTTTAATGCTAGCAAGTTATCCAGAAATTCAAGAAAAATGTTTTCAAGAACTTTGTGATATTTATGGTGATGATATTCAAGCTGATGTATTAATAAAACATGAAGATTTAAATCGTATGGAATATTTAGAAAGAGTTATCAAAGAAACAATGAGATTATTTCCAGTTGGTCCAATTTTAGTTAGACATGTTACTGATGATCTTGACATTGGTGATTATACATTACCAAAAGGTAGCTCAGTTGTTCTTGGTATCATGAAGTTGCATCGTTCTCCTGAACATTGGATTGATCCACTTAAATTTAATCCAGATAGATTTTTACCAGAAGAGACACGACATCCATTCTGTTATATTCCATTTTCTGCTGGTCCAAGAAATTGTCTTGGTTTTAAATATGCAATGATGGCTATGAAAGTACTCATAGCAACTTTACTTCGTCATTATGTTATTAAAAAAAATAACATTCAAAATATTCAAGATATTAAACTCAAGGTTGATCTCATGTTGAAACCAGTTGTTCCCATTAAAATTAAAATTGAAAAAAGACAAATTATCCATTGTAAATCATAAAATTTATATATATATATTTTTT

>CL6061.Contig1

CGACCGTTTGCGTCGTTCGTGATGATCTGCTTCGGTTGTTGGCGGGACATCTTGCCGGTCGCCGCGGCCGCCACTGTTGTGTCGGTGTTACTGGCGTACGCGTACTGCACGCGCCACTATGGCCACTGGGCGACGCTGGGCGTTCCGCACACCAAACCGGCACCGTTGCTGGGCCACTTCGCCGGGCCAACGTTGGGCCGCGAGTCGGGCACCGTCACCGTGGACACGCTGTACCGGCGGTTCGCCGGTCACCGGTACTTCGGGCTGTACCAGCTCCGACACCCCATGCTGGTGCTGCGCGACCCCGTGCTGGTGCACTCGGTGCTGGCCACAGAGTTCGCGTCGTTCCACGACCGGGTGATGAGTAGGACGTCGTTCGAGCACGACGGGCTGTTCAACAGTCTGGTGAACCTGAGGGGCGAAAAGTGGAAAGCGGTTCGGGCCAAGTTGAGTCCCACGTTTACCGTGGCCAAGCTCAAAGCCATGTTCGCCAGTCTGCACGTGTGCACCGGCCAACTCGTCGACAAACTGTTGCTGCTCACGCCCGGTGGCCAAGGTATTGTTAATGTCAGCGATATATCATCAAAATTTACAATCGATACAATTGGAAGGTGTGCTTTTGGGATAAATTGCAATACACTTTTTGATTCAAACACAGAATACCAACGAGCTGGGCAAGCGGTTTTTACACCTACGCTGAAATCGTCTGTATTAAACTTTATGAGGTTAATTGATCTAGGATGGCTTGTGGACTTATTTAGGTTACGTAATATGCCAGATCTGGTCTATGAATTCTATTCAAATTTATTTCAAGATACAATGGAGCTGCGTAAAAGTGAGAAAGAAGATCGTAACGATTTCATTTCGATTTTAATCAAATTAAGAAACGATGAAAAAAAAAACAACAGCCAAGTTGAATTATTTACTGACGATGTCTTAGCTTCAAATGCATTTATATTTTTTGCGGCTGGCTTTGAAACCACTGCATCTACTATGAGCTATTGTCTATACGAGTTAGCTTTAAATCAAGACATTCAAGTCGAATTGCGCAAACAAATTAAGCACACACTAAATGAAAACAAAGGGATTCTCACTTACGATGTGGTAAAAGATATGAAGTATTTGGATATGGTATTAAATGAAACTTTGAGAATGCATCCACCTGGCCCTGGTCTATTGAGAGTATGTACCAAAAAATTTAAAATACCAGACAGTGACATAACTTTGGACATCGGTATGAAAGTACTTATACCCACATATTCACTACATCACGATCCTGTGTATTATCCAAATCCAGAAGTGTTCGATCCCCTGCGGTTCACAGAGGACAATAAAGCTCTGAGACCGAATGGAACGTTCCTGCCATTTGGCGATGGGCCTAGAATTTGTATCGGTCTACGATTTGCATTGATGGAGGCCAAAACAGGACTAGCAGAAATAATATCAAAATTCGAAATACTTCCGTGTAACGATACAAAAATTCCCATTAAATTAAATCCAAGATCCATTCTACTGACACCAAATGAACCAATATGTTTATTG

>CL6400.Contig1

AGATAAAATTAAAATGATTTTGTTCAGTCTCTGGTCTTGAATATTAAAGATATCGGTCCGTCCGGTTTATTTATCAATAAAGATTTACTGTCCAACTTTGGTCCCGTCCAAGTCATCTGAAAGTTTCTGACGATTTTTATGAGCACGACCTGCAAAAATTGCTCTGACAAACGTCTCGCTATACACGTCCTAGGTCCGTGGCCAAAAGGTAAAACCAAATACGGACTGACCGACTCGTAATGGTCGCTACCTTTTAGCCATCTTTCCGGCACAAATTCGTCGGGTCGCCGGAAGTATTCCTCTTGACGACAGCTCACCTGATTTTGCGTCACCACGACCGTTCCTGCTGGGACTCTGTATCCCGAAAACACGCACTCCTCTGGCAGTATCCGACCGACGCCCACCGATATCGGATTCATCCGGAACATCTCTTTGACGACCGCTTTCGAGTACACGGCCCGCTCTAATATCCTATCGGTGACCGGTGTATTGTTGTCCGAGAGCAGGGCTCGTGCTTCATCGAACATTTTCTCCTGCACGTCCGGGTTTGATGACAAATGGTAAAGTCCGAAACAACAGGAGTACGTCGCCGTGTCGATTCCAGCTAACAAAGTGTCAACAGTCATTCCAACTACGTCTTTGTAATCTGTCTCGTCTGAGCTCAAGTATTCTCCTAATAATGATGTTTTGGAGTCTGTCGTTTTAATACTGGTTAGTTTTTCACTTACAACTTTTAGGGCGATTTTTTCAATGTGTTCGTGTCCTTTTTTTATACTTTTATACATGGGGGTTTCAAATTTTCTCCATAGTTGTGGTCCATTATCAGTCTTCAGTATCGCACTGTTTATATCGTGAGCTGCTTTGATCAATTTCGAACTTTCCGAATTAGAGTCCCATTCTTTGTCTTTGAACTGCTGAAGTCGTTTATCGAATGCGATGTAGTATGTTAGTTCGAGGAAGAGTCTAGATAGTTCAGGGCTGAAATCATCTGTGCGTCCTTCGCGTCTTATTCTCCGATCTATAAATTCTCCTACTACTGTGTTCGTACTGTTCACATACCCCTTGATGCATTGAACCTTACTTAAATGTTTTTGAAATGCTTTTCTAAGTCTCCACCAATCCTTGCCATTTGTAGGCAATAGTCCTCCAGTGTTGTATACGTCTGGTTTACTCAATCTGTACTTTTGTAATGCCAAATGACTACGTCTTTCCGGGTACCTGCCTTCTTTCCGATAAAGCATCTCGATGTCTTCCGGTTTGAATATCCACACTATAGACACGCCAGGTACAATGTCTTCTCGAACAACCGGACCGTATTGACGGAGTTTCGTCAATCCATTGTGATGTAATCGGTCAAATTTGTACTTACCGAACACCGGGAAGTATTGGTAAAGGGTGCCAACTAGCGGTAAAGATATTGGTCCTGGGATTTCGTTAAATTCTTTAAGAATCTTTGTTTCAGTTGACAAAAAACGTAGTACTCGAAATAAAGTCGATTTATTGCAATGATTGACTCGTCGCATAATGTTATTGTAACGTTCTGTACGCATGTACGCACCACGTAATA

>CL7847.Contig2

CTATACGGTGCGCAGTTCTCCGACGACCGACGCTACGTTTCGAGCTCATCAGCAGTCGTTTTAATAGTATAAAATATACCTAGTCAAATTCTGTTCAGCCGCACACCAACCACTTGTGTCTTGCATCATGGTGTATAAAATGCGAGCTCGTGGGTTTTTACCTCCGTATTAAAAACACATAAATAAGTTTATGATAATATAATTGTTTTCTCCAATTTTAATCGTTGAGTTTATAACTTACACATGTTTTATAACATACTACTACAGTGATCAACGGCAAAATCCAAAGACGACTGCTACCGCATAATTACTGCTCAAAATCCAACTCAATTTGGACATAGATACCAACGTACGACATCGAATTAAGTACATCATGTTTCCAGCGGCCGTCATAATCGTATTATGTTGTATAGTAGTGGTACTACTCCTTTATAGCTATTCGACATCCACATACAAATATTGGAAGACGAAGTCGGTGATTTTTGCCACACCCGTACCACTATTCGGAAATATTAAAGACCAAGTGACGCTAAAAATGACCCAGGGCGAGTGCTTGAAAAATATTTACAATGATTTTCCTCGTGAAAAGTATGTGGGTATATACCAGTTACAAACGCCGACATTGTTGCTCCGTGACCCAGAAATCATTCGGTCGTTTTTGGTGAAGAACTTCACACATTTCACGGACAGAGGATTCTCGTACGACGGTCACAGAGAACCGTTGACTAAACATCTTGTCAATTTAGAGGGTGACACGTGGAAAACTCTACGACAAAAGCTCAGCCCTACGTTCAGCTCTGGTAAAATTAAAAATATGTTAGGAATGTTACAAGGATGTGGAGTGCAACTTATTGAATACATGGAATCAACCATCAATTCTGGAAAGACGGAATTCGAAATTCGTGATCTAACGGCCAAATTCACTACGGACGTGATTGGCACATGCGCGTTTGGACTGGAATGCAATTCACTGAAAGACTCGCAATCCGAATTCAGAAGGATGGGATGTGCGGTGCTCAATTCTTCTCCGTCTTTAGCATTGGCTAAAATAGTCCGAGTATTCTTCCCAAAACTTTTCAAGGCGTTGAAACTCCGTACGTTCCCAGCCGAAGTACAACAATTTTTCTTGGGCATCGTAAAGCAGACAATTGACTTCCGAAACACTAACCAAGTGCGCCGGAATGACTTTATACAGCTGTTGCTTGAAATTAAAGATCAAAATCAACATCAAGAAAACGCGATTGGTTCAATTAAACTGACTGAAGAGCTGATCGCCGCTCAAGTATTCGTGTTTTTCTTGGCCGGCTTCGAGACGTCCTCCACGACACTGAGTTTTTGCTTGCACGAGATGGCCGTCAACCAAGACATACAAAACAGAGTATACGAAGAAATAAACGAGACAGCCGAAAAGTATGGACTGCCCTTCTCTTATGAAGCAATCGCGTCGATGAACTACTTAGAACAATGTCTGAAAGAAACGATGAGAAAATATCCACCTGTACAGGCGTTGGTCCGAGTGTGCACCAAACAGTTCCGGGTTCCCGGCACAGAATTAGACCTGGATGTGGGCACGGCCGTACTAATACCAGTGTACGCGATACATCACGATCCACAGTACTATCCGGAACCAGACACGTTCAATCCGGATCGGTTCGCCAAAGACTGCAACGGAGGCGGTGGAGACAACGGGAGACCCGCTGGAGTTTATCTACCGTTCGGCGATGGCCCGCGCATATGCATAGGTATGCGATTTGCCATGTTAGAAATGAAACTGGCATTGGCTCAGTTCTTGCATAGCTACCTGGTGACGTTGAGCGACAAATCCTGTGCGCGCATCGAATTCGAACCTGCGTCATTCCTGTCATGCCCGAAAGGCGGCATATGGTTGAACGTCAATAAACGAACACATTGAGACCGCACCGCATTGACCGAGATTTTTATATTAGATACCTAGATGTCATTTGATTACGTGTAATTTTAAATAAACCTATTATATAATAAATTATATATTTTAAACTTTAACTTTATTTTGT

>CL878.Contig1

GAAAAAAATAAAATAATATAATATTAACTTATATAATTAATAATGATGATGAAAAAATGTCCAATGCATAAAAACTTAAGAAATAATACCAATACAATTAAAAACGTAATACTTAATACAATTAACATTATGCAGTGGAAGTAGTTAATACAAAATGAATGTATTCATTAAAAAGTCAGTTAGCACTGTAGGATAGGTCTACGTCCGTTCTTCAAGTGTCACATTGATGCCGTTGTGTGCATTCAATACGATTTCCCCAACAAGACCTAATTTATCTTCTGGATCTATTGACTTAACTCTGTACGATTTTAACACTGTGGCAACGGCTATTTTGATCATAAGTATTGCAAATTTTTGACCAATGCAGTTCCTCGGTCCTGCACTAAATGGTATAAAGGAAAAAGGATGTTGATCATTTCTTTTTTCCTTTAAAAATCTATCTGGATCAAACTTCTCTGGATTTTCAAAATGTTTTTCATTTCTATGTAAAGCAAACACATTTATGAAAATCGATGTCCCCGGCAAAATAGTGTGATCACCGATTGTGAGTGGTTCGTAAATTTGTCTACCAATAAATGGAACTGATGGGTAAAGTCGTAGCACCTCTTTAATCGTTCTATCCAAATAGTCAAGACTGTGTAAAATTTGTACTGATAATTTTCCATCCCCGAAATTTGGAATTTTTTCGTCTAATTCTTCTAATATACGATCTTGTGCTTCTGGGTGTTTACCCAAGACATACATCATCCAAGAGAGAGTGACGGAACTTGTATCAACTCCCGCAAACATGAAAGTATCAACTTCCTCTTGAATATCTTTGTCACTTAATATTTTTCCATCATCAGATAATTCTAGCAATAAATCCAATAATGCTTTATTATATTTTTTACCATGATGAATTTCATTTTTATTTGCGTCTGACATTTGATTGTTGAATAATTTGTAATTATCTTTCCTATCTTTTATAACCTTTTTTGTAAATGTGTGTATAACGTCTATACACGCATTTTGTCTTTTACTAAGTGATGTTAAATTGAATAAAAAATTTGGTTTCAACCACGGTGTTATAAACCGTTTCTGCATTATTGCTGTAAGCTCGTCTAAAGCTTTAACATATTCCAGTTGAGAATTTTCTTGCGAGTTGAATTCGCATCCCATTGCCGTTATACCGATAGTATCAAGCGCAGCCAATTTAGCGTAAGGCTTTATATCAAATCCAGTAACGTTATTGAGTTCTTTTCGTAATACTTTCACTAAGGTTTTACACTGTTTTTCTATTAATGGTATGAAATCTTCGAGAATATTAAAATGAAATGTTGGTGTCAATAACTTCCTTCTTTTAAACCATTGCGGTCCTGAGTTTGTCACTAGTCCTGTACCAATGAACGGAAGTAGAAGATTATATTCTAAATTCTTGTCAATATGAACAGACGATTTCAAAATTTGCTGAATATGGTCAGCATCAGCCATTGCAATGAATGGTCGAAGACCAATCCACAATTGATAAGTGTCTCCAAATTTTGAATGCCATTCGAGTAAAAGTCTAAAAAAATCGGAAGGCGATCCGTTTAGCTGGAGTGCGTTGCCGAAGAGTGGTAGAGACGGTGGTCCTGCAAAGGAGCGAAACGAGGCAATGAACTCGCGCCTGCGAACCACCGGACCGATGAGTGCTATAGCAATGAGGGCTATGGCTATGCATATCAAAATCAACGTACTGTTGTCCATTGTCGTTGGCGTATAGCGCAGACTACCACAACAGTTCCCGTGTTGATCGAAGACCGCGTGTTAGGTATACCTGGTATATTATGTTATTATTAAACTGCGGTAGTATTCTGGTCCCGCGTGAATCAGGAACCGCCGATGGCGCTGCAACGGATTACGGGGTGTAAGCACACGATACAACACGTTTGAAACGCCGGTGCGGTGATGAGTGCGGACGAGTCGCGAAAACCGATAGGACGAGAAAAAAAATTTTCAATATTTCGAAACGTATAGTATTCACCACCGGTAGGTATACGCCACGTGATACAAAGACGCGACGTCAACGTAATATTGTAGTCGCAGGAGACGTAGTCTGATACGACATGCTATAATATTATTATTACAATGTGCAGTTATTTTGAACTATAATACGAACGCGATTTATAAACGACGGTAGTGCCGGTTTGGACACAGTGCGTACGGAAATTTTAACGAGATCGTAGGTACTTACTCAACGCAGCCGGTATGACAAATTGCCG

>U1003

ACGTGTGCTTTCGGTTTGAAATTAGGTAGTATGACAGACGAAGACAACGAATTTCGAAAGTATACGAAATTGTTATTGAAACCGTCGTTTAGACTAATTTTTACCTACCTGTTGCAATTGATTTCACCAAAAATGTCGAGTATTTTAAAATTGAGTTCAACCCCACCGGAAGTTATGGAATACTTTAACTCGTCATTTAAAAATGTAATCGAGTATAGAGAAAAGAACAATTTGAATAGGAACGATGTGGCACAAACACTTATGCAAGCTAGAAAAGAACTTGTACTAAATAATATCTCGTATCCCGAAGAGAAATTTACAGAAATGGATATAATTTCAAATGCAGTTTTAATGTATTTTGCTGGTGCCGAACCGGTATCTGATACGTTGGCTTTTTGTTTATATGAGTTAGCTATGAATAAACATATACAAGACAAATTACGTGAACATATTAACAGACTAAAAGAAAAACATGGTGGCGAATTTAGTAACGATTATTTGATGGATCTCTATTACGCTGATATGGTTTTAACAGAAACATTGCGTAAGGTCAACGGAACTATTGTTTTATTTAGAGTAGCTACTAAGGCTTACCAAGTTCCGAACTCGTCATTAATAATCGAGAAAGGTCAAAAAATAATAATACCGACTTACAGTATTCATCATGATCCAAAATATTATACTAATCCCGACGTTTTTGATCCGGAAAGGTTTTCTCTAGAAGAAAAATCTAAACGACTTAGTGGCACTGAATTATTGTTTGGGGACGGACCTCGATTTTGTGTTGGTAAACGTTTGGCTGAATTGGAAATGAAATTAGGTCTCTCAGAAATAATTTCGAAATTTGAAGTTTCACCGTGCGTAAAAACTGAAATTCCCATTCAATTTGCGAAGGCTGGAGGTGCTATCAGACCAAAAAAATGGAATTTGGTTAAGTCTAAAGCCAGTCGTTGCGGTTTAATAGTTACCTATTCAATGATTGTACCTATTTTGATTATAGTTTTGTTTGTTTGCATGGTTCAATTTATCAAAATTTTATAAATATTTAATTCATATAGGTACTCAATGATTGAACGATGAATATTTTAATATACTTAATTTTATTGGAATTTATAAGTTAGTAAATTTGAATAAGTGGTTTATGTTAATGATTTTTTAATATTCATATAAATTATTATATGTTTTTTTTTGTCTTGCGATGATTCAAAATTGTTGTGTGAAGTATTTGTTTTATGAAAATATCATACTATGAAAGTATGAATTAATTTAATTTAGCAATTATGTGTTGTAGTATTGAATTTTTTTTAATGATAATGATATTTAAAACTGAAAATAAAAAT

>U10506

TGGATATTAATTCAAATCCATTATCCAATTCAACATTTATTAACATAATAAATCAACAAAAGTTACAAGAAAATAAACATAATAATATAAAGGTAATAAAAATATAGTATAACAAATAAAATAAAAACAAGTACTCTTAGTTTATGTTCAAATGAATTATTTGTGGGTTTGTTGATTTTATTATGACGTAGGCCTCTCAGTCATTTTGAACTTTAATTCACCATCAGGTGCATACATGAATGTTACTTTGTAATCCAATGGTTTATGCAAATACTCAAGTTTGTGCGATCGTATCAACTTCGCCAAGAAAACTTGCATTTCTAAATCGGCAAATCTTCTTCCTAGACACATTCTTGGACCATGTCCGTATGGCAACGAAGCATATGGATGAATATAACCTGATGATTGTTTCAACCATCTTTCAGGTTTAAATTGTTTACAGTCCGTTACATATTCTTCCATATTTCCCGTAACAATTGTTGGAAAAACTAACTGAATACCTTTTGGTATTTTATATCCACAAATCACCATATCCTTTTGAAGGGTTCTACCATTACCAATCACAGTTGAATACATTCTTAATACTTCTTTGATAAACGCTTTTAAATAAACCATTTTTTCTAGTTTACTTGCGTCAAGTTTATCATTTTTATTTGGTAAAATTTTTAGTATTTCTTGATGTATTTTTTCTTGCTCTTCTGGTCGTGTGGCTATTTGATAGAGCATTGAACATACGGCCATCGAAATCGTATCAATTCCAACTAAAATAAGATCCAACGCCAATATGTAAGCAGTTTTCGGATCAGATTCATTTGCCAAAATTCTCTCAACCAAAGACAATTCGCTTTCATTCACCGATTGCGGTTTATTCTTCAATCTCAACATTGCATCGTCAATGTATTTCATACAAATTTCAATGAAATAATCCATATTTGAAACATATTTTTTCCAGAGTGTGGAAGGCAAGTATCGCCAAAACGGATACTTAAGTTCTAATAAAGCCACGTTACGGAGAGCGTATTTTGCAGCATTGATTATTTTTTGTGGTTCTGAATTTTTTTGGTAAATCTGGTCTTAGGCAACCTAGTCTTGCATCTAATGCCACACGACCGATACACTCCAAAGCCCATTTATGAATTTCATTATCAAAGTCTGCTGGCATTTCAGAATTTTCGTTTTTCATTTCTTGCATCCGTTTTATAAAATAGTCAGAAACTTCTTCAATTGGTTGAATATACTTCTTAACAGTTTGTGGTTGTAAAACAGGCTTTTGGACTTTAGTTCTGAATTCTCTCCACGGTTCTCCGTGTACTCCGACAACACCTGGTAGTCTCCCGAAAAATTGTCCTCGAACCTGGCTTTTATATTTCACCAGACATGGCATAGAAGGTCTAAATGGTGTATCACCTTCTTGTCTATACACTTTTTCTATTTCATCAGCATCGTAAACAAATAATAAATCCGGACGACCAACAAGATTTCCCAACTTTGCTATTTTACCATATTGTTTGTACAGTATATAGGAAACATTTGCCAAATCTGATATCTGATACGGACCTATAACTGGTAACATTCTCCAAGTGTTTCCTATAATGGGCCATGGCATGGGTCCTGGCACCTCGCTATAGTGTTTAGCTGTAATAAATTCATTATTCACTTCTAAATCTTCAGGTGATCCCGCGGAGACGTGTGGTGTATGTGCAGTAGCGGTTGATCGCCATCTCATTGTCTGAATTTGAAACTGACGAATATTTTTCATTGTGAATAATTTTCGAACAATATCATTGAAGTCACCGCTGCAGCAGTACAAAATATATTATTCGATGTACACTTACG

>U11141

GTAAAATTATAATCGTAGTACCTATAATTGTCGCGTAAAATCTGTTTACTTAGTTTTTTCTCTTGGTAACATGAAGAAACATACCACCATCAGGATTTGCTGGTATAGTTTTTGGATTCAGTTTTATTGGCAGTTTCATATTTTTGGACAGTGAAATGTTATACGTTGATAATATTCTAAACAACGCCACTTTTACTTGCATCATTCCGAACCGCATACCAATGCATAGTCTAGGTCCAGCGCCAAATGGTAAGTACGCATATTGTGGTCTATTGTGTTTTTCGTCCTCTGTAAATCTTTCCGGAATGAATTTTTCTGGTTCTGGCCAATATTCAGGATCGTTATGAATAGCATATACTGGTATGACCACCTGGAGTCCTTTATTAAGGGTGAAATCTGAGTTGGGTAATTTATATGGTTTTACTGCCTTCCTGGTTAAATTAGGCACTGGTGGATACTTTCGTAAGGCTTCCGATACAATCATATCTAAATAAGTCATTTCTTGCAATATTTCATATGTTAGTTTCCCGTTGTGTTTAATAAGTACTGAATCAATTTCATCTTGAGCTCTCATCTGTACTTCCGGGTTTTGGGCAAGTTCAAAAAATGTGAATGTTAACGTTATTGATGAAGTTTCATAGCCTCCAATAAACCATACAAAACATTGAGCTGCCAATACGTCCATAGTAAATTTAAAATCTTCTTTTCCTTCGTTTTCGTTAAGTACTAATTTACTTTCTTCTTCAGAAGCTAAATTCTGGCCTCTTTTAATTTTCATCAGTAAGTCTAGAAAGTCATTTCGTTGTATATTGTTCTCTTCTCTATATCTAACTGTATCATTCACTACATTATAGAAAAAATCGGCATATTCTTTTTTGATACTTCGTACTTTTAGTAATCCGATTAAACTTGGAGCCAATGCATTCAATGCATTTCTTATGATACTTTCTATACTAGTTGAAAATAGCATTCGACCAACCTTCCGAAATTCTGCATCAGGATTAGCCAACGAATTGGTTTCGAATCCAAATGCCACAATACTAATAACATCGGTTGAAAATCGAGCTAATAGTTCCTTAAATTCTACTTGTTCATCAATTACTGCCGGCGCTTCAAGAATTCTGACCATTTCTTGAGCTCTACCATCTATAAGATCAATCATGTTCTTTATTTTTCCAATGGTAAATGCTGGTGTCAATTTATTTCTTAAAACTTTCCATTTATGGCCTTCTAGTGTTAATAAATTACCAGTAAGCGGTTCTAGCTTGTCATCAAATACTATTCCACGGTTGGGAAAAGTAGAAAACTCCTTTGTAAATACATCTTTAATAATATTTAAATCTCGAAGCATTAGCTTCGGCTTTTGAAACTGATAGAAACCGACGAACTTGTATCCGTTAGTATTAAAATAAATGCTAGCATAATGTTCGCCTACCCATGTTTTCATTTTAGTTACCTCCTTAGTATGACCGTATATAATGTGAGGTTCTAACGGAACAGGACATCCTTGACGTTGCCAAAAAGTATAACGATATCTTAAGTAGAAATAAAATGATGTCGAAATTATGACGGCATAAGTCATCAAGTTTAATAGCAAACAATCAAATAAAACCATTTCTCCTCTTTATAATATCGAAAAATTATTTAATTTACGGCAGATGAAGTCGACAGTTACTCGAACATAACGCAAACATTTATGTTCTTAACGTATGTAA

>U11238

TTAACTTTTGCCATTGCCATGAGAATTAGGAAATCATGTTAGAAAAAATACACAAATAATATTTATTTTCTTTTAGACATTTTTATTTTGTAAGGTTTTGGTGTCATAAGTATACCACAATTTAGTCCATCGGGTAACCGATCTATTTCGATTATAAAATCATGTATTAAGTTGGCAAATACTAAGCTAACAAATCGCTGTGCTAACGCTTCACCAGGACATCGTCTCTTCCCAAATCCAAAAAAGTACATATCTTCATTCGTATTGAGTTTTCCATTTTTATCCAAAAACCTCTCTGGGCGAAAAACTTCAGGATCATCCCACTTTACCCGATCATGATGTACTGAATATAAACTCATCAAAATTGTTGTATTCTTAGGTATACTATATCCATTAAGATTTGTATTTTTTAACACACGTCTAGGACCTATAATTGGTGCTACATGATTAAGCCTCTTCGTCTCTAATAACACAGCTTCCATGTATGGATTTTTAAGAATTTTTTCGGCTGTAAGATACGTTCCCGGAGGTTGTATCTCGTCCAGAATAGATTGAATTTTAGCCTGGACATCTGGCCACCGAGCCATAGCCAGTATAGCAAAATCTAATGTACCACTTGTTGTTGATGACCCAGCAATAAATAAGTCCAAACAAACAGCTATCAATTGCTCTTCAGTAAACGTTGTATTGTTTATTTCTTCTTTTTTCATTTGGTGTAAATATGCGTCAATAAAATCCCTAGTAGAGTCAGTTATCGTCCTTTTGTGCTCGGAAATATTATTAGATATCAAGGAATAAAGTTGCTGATTTATATCAGTTATGAGTGAGTAACCCGTCCTGACAGGAGCCAAATACCGTATCCATGGCATCTGATTAAGCAGCCCACCAGCCATGTCAAATGCTGAACTTCGCTTCCTCATCAATGTTAATAATTTAGGGTCATCAAACTTAGTGCCCGCTGTCAGTTCCCATAACACATTCATTACAGCTGATTGCAAGTATAAGGTCGGCGTCACACTTTCCATCGTATCGATTAGACGTTGTATCATGAGTTGATATTCATCATAAATCAATTCGTCTACCCTTCTTTGTCCTAAACCAAGTAACTTCAAGTGTCTGACCGCAAACGATCTGTGTACTTTCCAAAAATCTCCGTCAGTCATTGTTATACCTCTACGTTTACCCATTGTCCTGAGTCTCATAAAAAAATTATCAGGCCGACCTTGAAATTCGTCTCTATGAAACGTTTCACTTAATAGATCGTTTCCAAACACAATAACGACATATTCCCGTCCCAATTTCAACCCAATGATATCAGAATTATACTTTTGACGGAGCTCCTCAAAAGCTAAGTACTGTCCATCTTTGTTAGACGACAATTTTGCTAATTGATACGTATTTCCTACAAACGGTATCCATTTTGGCCCTGGTGGATAGTTTTTTGGTTTCCTACACGAACGGAGAGCGATCAATAACAGTATAATTGTAACGAAACATACAAAATACCAAGACATTAATAACAGAGACATAATACTATTTATTATGTAGTTAGCGCCGGGACAGGTTAGAAAAACGATAGCACAAACAGTGCACGTGGTAG

>U11293

GGTCATGCTAGCCCTGTCCATGGTCGCCACCAGGCCGCACGTGGCCCAAGCGATCCGAGACGAGGCCGAACGAGTGACCGGCGGACAACGTCTGGTCCGTCTGTACGACAAGCCGGACATGCCGTACACCGAGGCCACTCTGTTCGAGACTTTACGAGTCGTTTCTTCGCCCATCGTTCCACACGTGGCGACCGAGGACACTACGATTAAAGGTTTCAAAATACCAAAAGGAACGTGCATTATACTTAATAATTACGAAATCAACACCAGCCCGGCTTACTGGGACGACCCGGAAGTATTCGATCCCAATCGTTTCATACACCGCAAGTCTGGTGAGAAGCCATGCATCCGGAAACCGGAATACTTTCTGCCGTTCAGCACCGGTAAAAGGACGTGCATTGGACAGCAATTGGTTTCCGGGTTCGGGTTTGTACTTCTCGCCGGTGTTCTGCAGAAGTACGAGGTAACGGCCACTGCTCAAATGGCAATACCCGAGGCGCGTCTTGCTCTACCGCCCGACACTTACCCGTTAATATTGAAACCGCTCGACGGATCTCCATAGAATCGGATGTCGAATATAAATATTAAATTGTTATGCAATCACGTTCACATCAGACTAATACGACAATCATTATCGTTGTATATTAAATTAGATAACTATTGTACATATCCATAAAAACATTTATACAGGTATCGAATCCCAAGACTGTTTGATTTACAATTTATTCGTATGTAAGAACATATAACAATAATTTTAATCGCGTCGACATTTGATTAAACATAGTATTATTGATATGTATACATTTTTATTTTATTT

>U12585

TCTGTAGAGTATTTGCTCATGATCTCTTTGACTTCGAGCTGATCGTTTTCCTTTATTTTGGCCGTGATAAACCGCATCAACTCATCGCTGCACGCCACTATCTGGTCGTGGGTCAGTTTTAATTTACCGGACGTGAATCCCGGGCTTAGTTTCTGCCTCATTATTTTCCATTTGGGACCGCACAAAAAGAACAGCCCATTAGCTAGTATGTTTGTCGACGGTTCCTTGTGAAAGCCGCGATCCGCGAAATGCGAGAAATCTTTGATCAGTACGGCGTTGATGAGCTCGGGATCACGGATCATCAGGAACGGCGTCTTCATCTGATAGAAACCACAATACCTCTCGTCGGCAAACCGTTTGTAAATTCCGTCAAATGTGTCAATTTGGTGTTCGAGCGTTGCTAACATTTTAAACGTGTTACCGACCAACGGCCACGGTGGCTCGTGGGGCACGTTTAGTTTTCGCCATTTTCCGTAAGTGCTAGTTGAATAATAGTAAACGATCGTCAATACTGCAATGGCCACGCCGATAACCGTTGATGAATCAAATAGATAATTGATGATGTCCCTCGAAATCATTATGACGATAAGTAATAATATTAGGTTGATGATAAGTTATTGTAATGTCCGTTGCCGAGTTTACTGCAACGGTGCATGTCACGGTGTGCGTATTCTTCGATTTCTGCCGTGCACAGTATGTAACTCAAACGTATG

>U13063

GTTTAATATCAGGTAATGATTCAGGTCGCAAAATCAATTGCGCACCTATTTTAATGTCATCTTGACTTCCTAATGTTTCAATTTTCATCTTTCTAATAACAGTTGATATTATAGTTTTCATTTGATGCATGGCAAATTTTTGTCCAATACAATTTCTTGGACCAGCACTAAATGGTATGAAGGCATAAGGATGCCTATTGATACTGTTTTCGGGTAAAAATCTATCTGGATCAAATGTCAAAGGGTTTGGAAAATGTTTTTCCTCTCGATGTAGTAAGTATGGTATTACTGCAACTACTGACTGTGGTGGAATTTGGTACTCCCTAATTTGAAGATGTTGTCTAAGCTTTCTAGTAACACCAGGAACACTTGGATATAATCTTATAGTTTCCTTTATTACTCGATCTAAATATGTCATAG

>U13950

ATTGAGCCCAGGCCATGTATCTATCGTGCATCACCGACTGGTGGATAACGCCGTGTTTAATCGTCGTAGTGACGATCGTCTATTATTTTTCTACTTCGACATTTAAGAAATGGGAAAAACTAAATGTTCCTTACAGTAAGCCGTTTCCGTTATTTGGTAACATTTTTAACCTGGCTTTATGTAAATATCATCCATTAGTTTTTTATAAAAATATCTACAACGAGTTCTCTGGTCATAAATACGGAGGACTTTATCAGATGAGAACACCGTATTTAATGGTCCGTGATCCAGAACTAATCAACAACGTGTTAATAAAAGACTTTTCGTCTTTCACTGAACGTGGTCTCTACAAGAATTTGGATGCAAATCCTTTGTCGAATAACATGTTTTACATGAAAAATCCTCAATGGAAAATAATAAGAAGCAAATTGACACCTGGTTTCACATCGGGAAAACTTAAATTAATGTATGATCTGATCAGAGAATGTGGTGATGAATTAATGAAAAATATTAATGATGATTTGATTA

>U13972

ATGTAAATTCATAGTAGTAATTCATTATTCATCCGTACTCCGTAAGCGTTCCCTGCGACAATTCACAGTTACGTTACATTATTTCGTAACAATGATTTCGTACCTGATCAATTTGATGTTTGATAACATCCTTTTGAGTTTAATTATTGTTTGTACTTTCCTTTATTATTATACGACGTCGACGTACGACACATGGAGAAAATTAAATGTGCCGTACGCAAAACCAGTGCCTATTTTCGGGAATATATTCAAAATGTTCACGGGCTTAGAGCATCAAGTGGACGCATTCGGACGAATTTATCAACAATTTCCCAATGAAAAATTTTGCGGATTTTATCAAATGAGTACGCCGTTTTTAATGATTCGTGATCCAGAGTTGATCAATACGATGATCATCAAAGACTTCTCGTATTTCACCGACCACGGTATTGACATGAACCCTTCGGTAAACGTAATGGCGAGAAGCTTGTTTTTTGCGACCGGACAAAAATGGAAAATAATGAGACAAAAACTAAGTCCAGGATTCACGTCTGGTAAGCTGAAGGGTACGCATGAACAAATCCGAGAGTGCAGCGATCAGTTGACAAACTGTATTCATGATAAATCCAAGGAAACCGACGGAATTGAAGTATATGAACTCGTCGGAAATTTAGCCACTGACGTAATTGGAACATGTGCATTTGGTATGAAATTGGATACAATTAATAACGACAACTCAAGTTTTAGACAGAATGTAAAAAAAGTGTTCAAACCTAGTGGTAAAGTAATTTTTCTTCAAATACTTGGAGTTCTATTTCCGAAAATCATTAAGATCTTAAAACTTCAAACTTCTCCAGTGGACGTAAATGCCATTAATTTTTTCCATTCAGTATTTAGAGAAGTCATCGAGTATAGGACCAAAAATGATGTGGTTAGAAACGACCTAACACAAACATTAATGAAAGCAAGACAGGATTTGGTTATAAGCAGTGATTATAAAGGAGAAGAAAAGTATTGTGAATTAGATATAATTGCAAATGCAATGTTGTTGTTTACGGCTGGTTCTGAAACTGTAACTGCCACAGCATCTTTTTGTTTTTATGAGTTGGCATTGCACAAAGATATCCAAGACAGATTACGTGCCGAGATAATTTCGTCGAAAATAAAGTATGGTGGACAGCTTAACAATGAATTTTTAGAAGATCTTCATTACGCCGATATGGTTTTAGATGAAACTCATCGTAAGTACACTATTATTACGGCCCTACTGAGAGGAGCTACACAAGATTATGACGTACCCGGAGAATCATTAACAATTGAAAAAGGACAAAAAATTTTAATACCAATTTATAGTATACATCATGATCCAAAGTATTATCCGAATCCAGATACTTTCGATCCGGAAAGATTTACTGCGGAAGAAAAATCTAAACGACCGAACGGAACATTTTTACCGTTTGGAGATGGACCTCGTCATTGCATAGGAAAACGTTTTGCTGAATTGGAATTAAAAATTATTTTAACAAAAATACTATCGAAATTTGAAATTTCACCTTGTGAAAAAACGGAAGTACCGTTGCAAATGAAAAAAGAACGTGGGATAACTTCACCGAAAAATGGAATTTGGTTAAATTTTAAGCCAATTGTGGATTAATA

>U14100

GCGCCGAACGGGACGACGGGCCGCGCCGCGAGGTGGGCCGCCGGTCCAGCACGGTCAAGATAAACATCACTTGCGTGGACCCGTTCGTGTTCCGCCGGCACTACTCGGAAGTGGACGACGACGATGACGACGAACGATCGGTGTCGTCGGGACACCGATCGCCGCTGGACAGCGGCACGGGCAGCGACCTGGACACGGGCAGCCGCCGGGACGAGAGCGACGACGGGACGTGCAGCAGTTGCGACTCGCTGACGAGCAGCACGGCGGATCCCG

>U15584

GTTTAATTTGAGCCCGAAAGCACATGCACCGATGACATCGGTCGCATACTTTCCCAGAATATCTCTTACTTCTATTTCATTGTTTTTTTCCCTTAAAATATCGATATTTTTCATTAATATATCACCGCACTCTTTCATCTGATTATACATATTCTTGAGTTTTCCAGATGTGAATGCAGGTGACAATTTGCTTCTTATTGTTTTCCATTGAGGATTTTCCATGAAGAACAA

>U16171

GGATCGTTTAACAACACGTATGAATGTCCCAATACATTTATATGCACCAATGGCCCATGATGTTTAAAGATTTTACTAAAAAACGGCAACACATCTTTAGATTTGAGTGTAGATAATTGAAAAGAGAACTTTAAATCAGTCAAGTATCCTCGTTGCATAGATGGTAATTTTGCACACAAGCGGTAATGTTCCTTGGGTCTCCGAGCACAGAAACACGTTGTGAATATCAAAATTAAAATTATAAAAACCAATAACAAACTTACTAACAAAAAAATTGTCA

>U17640

GTCAACATACATAAAGGTTAATACAGGATAGATTAACTAACCTACCCTACATAGGCATAACTTGGACTCTTTTCACATGGAGGCAAAAATATTTTTTGTAGCAAATGAACATAATATTATGTTTAAGTTGGTGGGGGCAATGCCCCTGCCAATTTTTGGCTGTGAGGGCAGTTGCCTCCATTTGCCCCCACCAAGTTACGCCTATGCTAACCTAAGTTAACCTAATGATGTCCTACATAATGGTTTACTATAAG

>U21052

GCTTCATCATTAAATCTTTCTGGATCAAATTCATTTGGATTTGGAAAATATGTTGGATCACGTTGTATCGCATAAATTGGTATCCAAATTCTGGTACCTATTGGAATCGTAACTTTAGTGCCAGGCAATGTGTATAAAGCTGTTGATTTTCTCGTCAACGTTGTAACTGATGGATATTTTCGCAATGTTTCTTTAATAACTTTATCGAGGTATTTCATATTTTTAATTCCTTCATATGTTATTTTGCCATCTGAATTATCTAATTCATTTTTTATTTCTCGTCGTAATTTATCACGTATTTGATGATTCATACCAAGCTCCAATAAACAATTACTCATTGTTGAAGAGCTCGTTTCAAATCCAGCAATAAAAAATACTAACAGCTGAGCAGCTAAAACTGTATCA

>U21285

ATGTGCTCAAAAAGATATGATTTTGATGATGAAAAATATATAAAATTAGTAAGAAATTATGATGAAATATTTTGGGATATTAATCAAGGATATGCTGTTGATTTTTTACCATGGCTCAAACCATTATATGGTAAAACAATGGCAAGTTTACGTAATTGGTCAACTGAAATACGAGCTTTTATACTTAAAGAAATTATAAATGATCGTCTTGCAATATTAAATAATAAAAATGATAATTCACCTCAAGATTTAACAAATGCATTATTACTCCATTTAACAAGTGATGAAACTGAGCTAACATGGGATCATGTATTATTTGAACTTGAAGATTTTTTAGGTGGTCATTCGGCTGTTGGAAATTTAGTTATGTTGATATTAGCATATGCGGCAATGTTACCAGATGTACAAAAAAAAATTCAAGATGAATGTAAATTAATTGGAACTAAAAAAGAACGTTTAAATGGACTTGTTACACTTGATGATAGATCTGATATGCCATATACTGATGCTGTTATTTGGGAAACATTAAGAATATCAAGTTCACCAATTGTACCACATGTTACATCATTAGATACTGAAATTAATGGCTATCATATCACCAAAGACACTGTTGTTTTTATCAATAATTATGAATTAAATTTAGGTGAAAAATATTGGGGTGAAAATTCAAAACAATTTATACCAGAAAGATTTTTTCAAATAACTAATGGTAATATTCGTGTTGTTAAACCAGCTTATTTTTTACCTTTTTCCACTGGTAAAAGAACTTGTGTTGGTCAAAAACTTGTTCAGGGTTTTGCATTTGTTATTGTTACATCATTGCTGACACGTTATGATATTTATCCAGTCGATAAAAATCTTAAACAAGAATTACGTCCAGCATGTGTTGCTGTACCACCTGATTCATTTAATCTTGAATTTAGACTCAGAAATATAACAAATTAAATATATATTTTTTATTATAAAACAAACTAAAAGAAAAATTCAAAAGACTG

>U21773

ATTGTTGGAAATTTTTATAAAGCTGCATTTGGAAATATATCATTGATCGAAAATGTAGATGAAGTTTATAATAATTGGAAAAATGAACAATTGATCATAGAAAAAAAAATAATATTATTCGTGGTGACCTTGTGGATGTACTCAAGGCTATGAAAGATAATAAAAATGAAATTGATATAAATTTGAGTGATAATATTTTAGCTGCTCAAGCAGTAGTTTTTTTTGCAGCAGGATTTGAAACATCATCTACAACACTTTCATTTGCCTGTTGGGAACTTGCACAAGCATTAGATTGTCAAGAAAAACTTAGAGCTGAAATTAAAGAAATTCTTGATAAATCTGGAGGAAAATTGAGCTATGAAATTATTAATGAAATGAAGTATCTTGATATGGTTATAAAAGAAACATTGAGAAAAAATTCACCAGTTGGATCACTCGTAAGAGAATCAGTAGCACCATGGAAAATTCCAGGAACTGATATAACATTGCCAAAAAAAAACAAAAGTTTTTTTTTCCTATATATTCATATCAACGTGATCCAGAATATTTTAGAAATCCCGATGTATTTGATCCACTGAGATTCAGTGAAGAAAGAAAATCAGAAATTATTCCTGGAACTTATAGCCCATTTGGACATGGACCAAAAAACTGTATTGGTGAAAGATTTGCAAATTATCAAACGAAAATAGGATTAATATCAATTGTAAAAAATTTTATAATTGAACCATCGGAATTTACTAAAAAAACATATGTTATTGACAAAGCATCACTCGTTCTTGCTATGAAAGGAGGTGTTCATCTTAAGCTTGTTCCTTGCAATTAAAAATTTTTGTTTTAAAAAATAACTAACAATCAGAATTAATGTGTAAAATAAAATATATATGTTTAGAAAATAATATGCAC

>U22248

AATGATATAGGAAAAAAAAAAAGAAGAGCTTTTTTGGATTCTTTGCTTGAGGCAAGTGAAAAAAGTGAAAGACCTTTGACCATGGATGAAATACGAGAACAAGTCAATACATTTATGTTTGCGGGACATGATACAACTTCAGCATTAATTAGTTGGACACTTTTTTGTCTTGGAAATGACGATGAAATACAAAAAAATGTTCATGAAGAAATTGATGATGTCTTTGGTGATTCTGAAGAATTAACAAATACAAAAGACTTGGCAGAATTGAAATATCTTGATAGAGTAATAAAAGAAGTACTCCGACTTTATCCAAGTGCACCAACAATAAGCAGAAAATTAACTGAAGATTTACAATTTGGTAAATATACTGTTCCTGCTGGTTGTAATTTAAATTTGCATATTTATCAACTACATCTGGATCCTGACATATGGCCAAATCCAAAAAAATTTGATCCAGATCGATTTTTAGATTCAAACAAACGACATCCATATGCCTACGTACCATTCTCAGCTGGACCAAGAAATTGTATTGGGCAAAAATACGCTCAACTTGAGGCTAAAATGGTTTTAACTGAAATACTCAGAAAATGGAAAGTTAAAAGTCGAGATACTCATGAAACAATGAAATCATATACTGCTGTTATTTTACGACCTTGTGAGGG

>U22700

GAATCTTTAAATGCATTTGGACTAATTGATGTTATGAAATCGTCACAAATGATTCGTATTCCTCTAATACGTACCTCGACGAAAACTGCAAAACTTAAAGGTCTAGCAATTTTTCCCTCATATACATTGACAGATGATATAGAATTGTGCTTGAAACAAAGTTTCAGATTATCATGTAATTGACATCTCGTTTCAATTTGAGATTCCATAATATTCCAAGTCATAGTATCTTTTACCAGTTTATTATTATGATCTACATTTAAGTGATCGAGATTTGTCATATTGGCAAATGCACCATTTTCAATCAGAGAAATATTATTGTATGATAAATCTAAATTTTTAATATTTTCGCCTTTGAATGTTTTTTCATGTATTGATT

>U22886

ATCGAGAAACGCGAGGCGTCTTTTTTCCCCAACATCGTTTTCGTCAATCTCATCGAGATCATCTCTTACATAACGCATATTTGAATTTTTGTTCTCTCTATCAATGTTGACAGTAGAAGCCTCAATATTTTTTCCTGTGGCATAAACTTCAGGAAGTGATAGTTCACCTTTCTCAGCAAGCTCTTGCTGTTTTTGTTTGATAACATTCCGAGTTAAACTATGAATGATTGATAATAATTGTTCTTGCTTTGTTGCAAAGCTAGTGAATTTGAAAAATGGATCAATTCGTAGCATGAAGTTGTACTG

>U24187

TAATTGAAATTAAAAATGCTAAATCTGAAGGAAGAGAAACAAAATTATTTCAAGGAAAAAATCATGATCGTCAAATGCAACAAATACTTGGTGATCTTTTTTCGGCTGGCATGGAAACTGTAAAAACAACGTTGGAATGGTGTGTAGTTATGATGCTTCATCATCCAGATGCTGCAAAAGCTGTACAAGAAGAACTTGATCAAATTGTTGGAAGATCAAGAATGCCAAGTTTAGAAGATTTACCATATCTACCAATAACTGAATCAACAATACTTGAAGTTATGAGAAGATCAAGTATTGTACCACTTGGTTCAACTCATGCAACAACTCGTGATGTTAGCTTAAATGGTTTTACGATACCAGCTGGCGCTCAAATTGTACCTCTTCTTCATGCCGTTCATATGGATCCAGAATTGTGGGCGGAGCCAAATGATTTTAGACCAGCCCGATTTATATCCGCTGAAGGAAAAGTAACAAGACCCGAATATTTTATGCCATTTGGTGTTGGTAGAAGAATGTGTCTTGGTGATGTACTTGCTAAAATGGAATTATTTTTATTTTTTTCTTCAATAATGCATACATTTGATATTAAATTACCTGATGGTGATAAATTACCAAATCTTAAAGGTAATGCAGGTATAACAATAACACCAGATTCATTTAAAGTATGTCTTTTACAACGAAAACTTGATATAACATTATGCCCAAATGATTTGCCAAGTCCACTCAGAAATATTGGTAGTCATTAAATTATAATG

>U27971

AATACACACGTTGGGTAATACACTTGTATTTTTATTTCATCTAATTGGAACAGATAAAGAAATTCAAAAAAAACTTTATGATGAAGTTATTAATCTTGCACCAGAAGGATGTGATCTTGTTGCTGAAAATCTTAGAGGTGCAAAATATTTAAGAGCATGTATTACAGAAGGTTTTAGAATGGTTCCAACAGCTCCATGTATTGCTCGGCTTCTTGATGCACCAATTGAACTATCTGGATATCAATTAGAAGAAGGAACTGTTGTATTGTTACACACATGGATAGCTGGTTTAAATGATGATAATTTTTATAATGCAAATGAGTGGATGCCTGATCGTTGGATTAAACCATTATTACCACATTCACCATTGTTGGTGGCACCATTTGGCGCTGGTCGCAGAATATGCCCTGGTAAACGTTTTGTTGAACAGGCACTACAACTCATCGTTGCCAAAATTATTCGAGAGTTTGAAATTGTAGCTGTTAAAGAACTCGAATTACAATTTGAATTTATTATTGCACCAAAAGGTCCAGTACCAATTATTTTTAAAGATCGTTCAGAAAATATTCACGATGATAAATATACATAAAAATTTCATTTCG

>U28800

TCAAGGTGATTTCTAGCCGGTGTTAATTAAAAATGTCAGCTCCAATAAGTACAGCGTTAGATAATTTAAATTCCGCAATAAAATCAAATATAATACCAAATCAAGAGTATCTTCTTCAGGGTTCAATTTTAGATAGTGCTGTTGAAGTATTACTGCATCGTCTACGAGGATTATGTGATAATGTTGACAGTGGTACTGAAACATTTCATGATCATGAAATGTGTTTTAGTATTAGAAAAGGACCACCACCAGAACAACCACTTTCATTACGTGTAAGAAGAGCATTGGATTATCCAGAAATGCCATGGCAATTACGTTATATTGGTCAACATGAACTTGGTGATAAATCAAGACCAACAATTGTTAGAAGTAGTATTGATATTGCAACAAGTAATACTGTTGTTGAATTTTTAAATGAACTTGGTTGTAGACTTGATTATGAATATATTATACGTGGTTATATGTTTCGTAAAGGTAGAATGAAAATAACTGTATCAAAAATATTTAAAATGGCACAAGGTAAAGTACCAGAAAGTGTTGAACCAATGTCACAAAGTTATTTAGTTGAATTATCTGTTCTTGCACCAACTGGACAAGATGCTATTGCTGAAGATATGAGAATATTTGCTGAACAATTAAGACCACTTGTACAACTTG

>U30953

ATTGACACTGCAATTGAAAGATTAAAAACTAAAAAAGCTGTTAATGAATTAGACTTGTCTTTGGTTGAAAGAATACTGGCTAATGAAAAAGATCCAAAAATTGCTTATATTTTAGCTTTAGATTTGATATTAGTTGGCATTGATACTATATCTATGGCTGTTTGTTCAATTTTATATCAACTTGCAACTCGATCAGAAGAGCAAGAAAAAATTTTTAAAGAACTAGAAAAAATTATTCCTGATCCATCAGTGCCACTGACAACGAAGCATCTTGATCAGGCTATTTATACAAAAGCATTTATTCGTGAAGTTTTTAGGGTTTATTCTACAGTTATTGGAAATGGCAGAACACTTCAACATGACACTGTAATTTGTGGCTATCATATACCAAAAGGAGTCCAAGTTGTTTTTCCAACATTGGTAACGGGACAAATGGAAGAATGGGTAGATGATGCAAAAACATTTAAACCAGAAAGATGGCTCAAAGAAAATTCAGATAAAAAATTACATCCATTTGCATCATTGCCTTATGGACATGGTGCTCGTATGTGTCTTGGTAGACGTTTTGCTGATCTTGAAATGCAAGTATTACTTGCAAAGCTCATACGAAATTATAAACTGGAATATCATCACAAGCCATTAAAATACAAAGTTACTTTTATGTATGCTCCTGATGGTGATTTAAAATTCAAG

>U31346

GGTGTTTATGGAACAAAAAAAACCAGCACTAGTTGTCAGTGATCCTGATTTAATACGAAATGTATTAGTTAAAAATTTTTCTAATTTTCATGATCGTGGAATACCTTTTAATGAAAAAATTGATCCAATAACAGCACATATATTTTTTCTTGGAGGTGCACGTTGGAAAAACTTACGTGCTAAATTATCACCTACATTTACTTCCGGCAAGATGAAGCAAATGTTTAATGTAATGTACAGTTGTGCAGATAAAATGACAAAACATCTTGAAAGTATTGCTAAAAATCGAGGAATTATTGGAATAAAAGAATTGTCTGCAAGACTCACTACTGATGTTATTGTTTCAACTGCATTTGGAATTGATAGTAATTGTATAGATAATCCTGACAGTGAATTTCGTTATTGGGGAAAAAAAGCATTTAAACCAACTTTAAGATTAGTTGGTATGCTTGTTGCACCAAACCTTATGGATATATTTAAAATTCCAAGTACCGATCCAGGTGTTTCAAAATTTTTTACAAATGCATTTAAAGATACTGTTAATTATAGAATTAAAAATAATATTGTTAGACCAGATTTTATG

>U31347

TCGATTAAAAGTTCAATACCGCTTTTTGACATTAAGACAATTGAAAATGATTCAAAATCAAGGACATCTGGTGTGCTTGGAGCTTTGCTGAATTTATATTTTGATAATAAACTAATCAATACAATTTTTACTTGAAGAAATCCAAATCTTGAACCAATACAAATTCTTGGACCTTCACCAAAACCCAAATAAGCATAAGGATGTCTTTCTGCAGTATTTTTTTCATTAAATCTTTCAGGATCAAATTTATGTGGATCAGGATAAATATTTGGATCACGATGTAGACCCAATACTGGAATAATAATATCCATACCTTTTGGTATTTTTAAACCAGTATCTCCTAGTTCAATTGCTTCTGTACATTGACGATTCAAAAATGGAAGAGGTGGATATTTTCTTAATGTTTCATTAATAACTTTATCAAGATATGTCATTTCTTGAGTTGATTCATATGTAATTTTTCCATCATGATTTTTCAAAACTTGATTTATTTCAACAGCCAATTTATCTTGAATGTGTTTGTTACATTCTTTTGATAGTTCATAAAGACTGTATGAAATAGTTGTTGAAGTTGTCTCAAATCCAGCTAAAAAGAAGACAAATGCTTGGGCAACAGCTTCATCAATGGTGATATTACCATCAATTGTTTCTGAAATATGAGATGGTTTTCCATCATCACCTTCAACATATCCTTGTTTCATTAATTGTATCAATA

>U33083

ATTTTTTATATGATAAATTTAATAATAATTTAAATAATGATAAAATAACAATTGATTTAAAACTTGCATCACAAAAATATACAACTGATGTTATATCATCATTGGCATTTGGTGTTAGAACAAATTCATTTGATGATTCAACAGCTGAATTTTGGAGACGAAGTATTTATCCAACAACAATGACACTCAAACAAATAATTCGTTTATTTTGTATATTTTTTTTTCCAAAATTAAATAATTTACTTGTACCTGGAACATTGCTGGATCATCATACTGATTATTTTCGTGATATTTTTTGGCGTTCAATGGATATGAGAGAAAAATCAAATATCGAAAGAGGCGATGTTATGGATACATTATTAAAATTAAAAAATGATAAAAAAGATTCAACATTTACTTTTACTAGAGATGAGCTTGTTGCACAGTCAGTTGTATTTTTATTAGCTGGTCAAGAAACAAGTTCAACGACAATTGCATTTGGATTATATGAGTTGGCAAAACAACCAGAACTTCAAGATCGTGTTAGAAATGAAATTCGTGAAAAAATAAATAATAAAGGATTTACATATGAAGCTGTTCAAGATATGAAATATTTGATGCAAGTTATTAATGAGACATTGAGAATGTATCCACCAGCTCCACTTTTAGATCGTGTTGCTGAAAAAAATTACACGATACCAGATACAGACGTGGTCATTGAAAAAGGCACAGCGGTGTATGTAGCTCTACCTGGTATTCAAATGGATCCAAAATATTTTCCAAATCCTGATATTTTTGATCCAGACAGATTCAGCGATGAACGAAAACATGAAATTCAACCCTGCACATTTATGCCGTTTGGCGAGGGTCCACGTACTTGTATTGGAATGCGAGTGGGTATGTTACAAACAGCTGTTGGTTTTATACAAATTTTAAAAAATTATGAAGTTTCGATTAATCCAGCTTATAAAACTGAAATTCATCCTGGAAATATATTTCTTGGTTTTAAAGATGGTTGTAATCTTTATGTTAA

>U34799

TAAATTTCATTAATTATATTTCTATAAACTCAGAAATTTAAACAATAACATGATATGTTTTAAACTCTGAGTATTATTTTAATTAATATTAAAAAAAATTTCATTTATTCAACTCCAAACATCAGTTGAATAACGTTTTTGTGTATCCATTCTTTTGATGTAACTTGTTGCTTCCATTTCTGTCATTTTGCCTTTTTCTTGGACGACTTTTAGTAAAATATTATGAACATCACGTGCCATATTACGTGCATCTCCGCAAATATAAACATGTCCATTTTTAGCTCCAATAACATCCCAAATTTCATCTTTATTTTGTTCTAATAAATGTGTGACATAAATTTTCTTTTCTTGCTCACGACTAAATGCAGTATGTAACTTTAATGTACCATTTTTAACATACTGTTCAAGTTCATCTTTGTATAAATAATCTTCATCACTTTTTCTACATCCAAAATATAATATTGTATCACCAACATCTTTACCTTCTTTTTTAGCTGCATCACGTTCTTGTATAAAACCACGAAATGGTGCAATACCAGTACCAGGACCAACCATAATAATTGGTGTTGTTGGTCTTGTTGGTAATCTAAATTGTGATTTTCTAACAAATATTGGTACACATGTTGGTGGATTACTTGGATGTTTTTCTTTAAGCCATGTTGTTGTAACACCTCTATTAATTCTACCAGTTGGTGTTTTATATTCAACAACAACAGCTGTTATATGAATTGAATTTTGATGTACTTTACCAGATGATGATATTGAATAATAACGTGATTGTAATCTTGGTAATATTTCACAAAGATGATCAAGTGCTGGTTTAAGACTTTTAATATCTTCTAATATATGTACAATATTACGATTTTCTTGTACAATCCATTGTTGATATAATGCTTTACCTTCACTTGTTGTTGATGACATTAATTTTAATTTATCTTTATCATCTTGATCAGTTGCATATTCAGCTAATTCTTTTAAAACATGAGTTCTTGGATTACTTGTTATATCAAGATAACTTGTCAATGCTGTTCTATAACTACATGGACATGGAAATGGATGTTTTTTAGTTGATTCTTCATCAAGATTTGTTAATGTTATTATTTTATCAAGATCTTCTTGGCATATTTCACCAATTTTATTAACAAGCTCATAATTATTAACTGGATAAACAGCAACATGATCACCAGCATCATAACGCATTTTTGAACCTTCAATATCAAATTCAATATGCATACATGAACGTTCTGATTTTTCACTGTGTAACTCACGATTTACTTTAATTGGTGCTAAATATGGATTTTTAGCATCATATGGAGGACGTTGAGTTTTTAATGAATGTAAACGAGCTATTTCACCAGTATAAATACGATCAGCTGGTAAATCAAGATGTTCTGTTAATTTATATTGACGTATACTAACATCTTCAAGTGCACCTTCAATACCAAAAAATTCACAAACTGTTGGCCAAAATTTATCCTTCCATGTTATAAAATCATCTTCAATATTTGCATCATCATCACCAAGACCAAGTTCATAAACTCTTGTTGCTCCTAATTGTTCCAATCGATGATCAATATAAATTGCAACTTCATTGTAATGCTCATAGGTTTTATTACCAAGTCCAAATACAGCATAATTTAAACCAGTTAAATCAATACCCTCGCCATTTTTTAACCAGTCAACAAATTCCATGGCATTATCTGTTGGATCACCTTCACCATAAGTTGCAAGTGCAAATACAGCTAAACTATTTGGTATTGTTTTTAAATTAACCAATTCCTCCATGTCACATTCTTCAGGATCAGCAACCATACCCTTCATTGAATATCTAACACCTTCTTTGGCAAGACGACCAGCAAATTCTTCACCAGTACCAGTTTGACTACCATAAAATACAACAAGACTACGTCCAGATGTTTGAAGTTTTTTAATAAATGAATTTTCAATTGGTGTTGTTGCTGTATATAATGTTGGTTGTATTGAATATGATTTTGTTGATGGTGAATATTCATCTTGTTTTTTATTACGATAATAAAAAAAACCAATATGCAAAAATTGCTAGAGCAAGTGTCAATGCAATATCAAGAAAACTAAGATTAAATATTGATTCATCAATATTATCAATGCCATCACTTGGTTCTTTGTCTGACATTCTTACAGCTTTTGAATTTAATGTGTTAATTGTCTTTTAAATACTCCAAACTGACAAATTCCTGGCCAAAAATAAACAACAGCTTCAAAATTTACTCTTTTTTTTCTTCTAATTATATTCAAATGCAGTTTTCATTTGTAGTATTTTTTTGATAATACAATTTGACACTTGTTTATGATAAATA

>U35202

AAATATTATTATTAAAATTATAATAAACTTCGTCTTTGCATTTTAATCCAATAGCCATTTTTTATCGTAGTTAAAAAACTTCTTGGATCTAATTCAAATGGTTCTTTCATTTTTTTTGATGGTTCCATTACATAATTCATTAATATTGTTGCCATTACAACTTTAATTTGCATTATTCCAAATTTCATTCCTGGACACATACGTGGTCCCTCTCCAAATGGTAAAAATGCATAACGATTACGTTCTTTATCATAATTTTTTTGATCAAATCTTTCTGGTATAAATTCATCTGGATTATCCCAATATTGTGGATCTTTGTGTAAACCAAAAACTGGAATAATAACTTCATCACCTTTATTTAATTTACAAATTAAACCATCTGGTCCTTTTAATGTTATTGGCTCTGAGCAAATTTTTAATAATCTTCCAACTGGTGATATACATCTTAATGATTCTTTTATAATTTGTTCAATATATGTCATATCATTTAATGCATCATATGTTAATTCACCATGTTTTTTAAATATATCATCAATTTCTTTTCTTGCTTTTTCTTGTATTGCTTTATTTTTAGCTAAATAATATGACATTATACTTAATGTCATACTTGATGATTCATAAACTTCAACAAAAAATCCAAAT

>U38029

GACAGTATGAAGTTGGTGATGTTGCTAAAATATCACAAATATTTCATGAAGAATATGGTGAAATAACAAGATTGTCTGGTCTCATTGGACGACCTGATCTTTTATTTGTTTATAATGCTGATGAAATTGAAAGAATGTATAGACAAGAAGGACCAACACCATTTCGTCCATCTATGCCTTGTCTTGTTAGATACAAAGGTGTTGAAAGAAAAAAATTCTTTGGTGAATTAGCTGGTGTTGTTGGAGTACATGGTGAACCATGGAAAGAATTTCGTACAAGAGTTCAGAAACCAGTTCTTCAACCACAAACAGTTAGAAAATACGTTGGACCCATTGAAACTGTCACAAAAGATTTTATAACAAGAATTGAAAAGATTAAAAATAGTGAAGGAGAATTACCAGCTGATTTTGATAATGAAATTCACAAGTGGGCATTAGAATGTATTGGACTTGTTGCTCTTGATGTAAGACTGGGTTGTCTAGCAGATAATTTAACACCAGATTCAGAACCACTAAAA

>U38966

GTTGGTCTCCTGTCTCGTGTTCGAATTGTAACAGGATAACCGTGAACACTTCTCATCAATATACCTAATTTTAACTTAATGTCTGGTAATTTTATGTCTGTGTGTACGCTAAAATGTCTCAAGAATGTTGACAAAGTGACTTTCATCGACAACATGGCATATTTGGATCCTATACAACCCCTTGGACCACCACTAAACGGAATAAAGGCATATTTATGACGTTTTTCGATATTTTCTGGGCTAAATCTTTCAGGATCGAAGTTCCAAGGCTTTGGGTACAAATCAGGGTTATGATGTGTGGCTAGTGGGCTTAAGATACACGTTGTCCCTTTTGGCAATACGTAATCATTTGAAAATATTTTAAGATCGTTTTGAAGTTCTCT

>U40422

ATTACCTTGTTGCATTTATCATGTGCTATTTCAACAGCTTCTTTGAAGGCCTTGCCTCTTGATGTTTTGTAAAAAATAAAATCTGATTTTAACCATAATCGCATTGCTCGTGCAACTGCGTTTGTAGATATTGTATGAACAGCTTTGATGTATTCATTTTCAACGTCATCTTGAGCGTGCGCATTCATTCCCAAAGCAGTTTCATATATAATATCAAGTGCACATTTTCCAATCATATCAAA

>U43512

TTTCGATACAATGAACAAATTTCCATTACAAGTGATCAAGGAAAAGAAAGTAAAATTTGACCAGATAAAAAAAGCTATTAATGCTAAAATAGACATAATTGACAGTGACAATGAAAACCAATCAAAACGGTTTTTGGACATATTGTTCAAACTGAACATTGACGGTGGAAACTTATCAGAGAAAGATATTAGGGATGAGGTTGTAACAATGATGTTTGGGGGTAGTGATACAAG

>U461

ACCGGGACGCAACAATGGAAGACCTCAAAGCGATGACAAATTTAGAAAGAGTGATAAAAGAGACAATGCGACTTTATCCGAGCGTGACGGGTATCACGAGAACCCTCAAAGAGCCGCTCCACCTCGAAAAGTATACAATACCTTCTAAATCGGTGATGGTCGTCGTTCCTCATCTATTACACCGTGACGAAAACATCTATCCCAATCCGGAAAAATTCGATCCCGACCGGTTTTTGCCTGAACAATGCAACGGACGTCATCCTTACGCGTACATCCCGTTCAGCGCCGGGCCAAGAAATTGCATAGGCCAAAAGTTTGCTATGTACCAAATGAAAACAGTATTGTCCACGATCTTGAGGTACACGATTGTAGAGACGTTGGGGACGCAACAAAGCATCGTCATCAGTACACAGCTGATAATGAGAGCTGATTACTTGCCGAGCGTGAAAATAACACCAATCCCCAACAAGAGTATCAAAAGTCATACGTAATTTGTTAACCTTTATTTATAATATCAGCATAATAACTTAATATGAATGTAAATATCATGTACATACTATGCATATAACACAACATATTTTTTTGTTTTCAATTATAATTTTTAGCAAACCATATAAGCCACCCAACCAAGTATACATTAATAAAGGATAACG

>U4699

CGGAACAAGATGGATCTATTGGAAATTCGGTTTGTATAAACTGAATGCCGTACATCTAGCATACAAAGATATGTTTAACCGGTATGGAGATATTATTTGCGAAGAGGCATTATGGAACATACCAATTATAAGTGTCAAAGACAGAGATTTCATCGAAAAAGTACTTAGAGATAGTGGAAAGTACCCTATCCGGCCTCCAAATGAAGTAACGGCGAATTACAGAAAGTCTCGACCGGATCGCTATACAAATACAGGACTTGTGAATGAACAAGGTGAAGTTTGGGCGATGTTAAGAAATAAACTGACACCGGAACTTACTAGTCCTAGAACAATACAACGTTTCTTGCCAGAAGTTAATCAATTAGCAGACGATTTTATCAACTTGATTTCCCAAGCGAGAGACAGCAATAACGTAGTCGAAAAATTTGAATCGTACTGTAACAGAATGGGATTGGAAAGCACATGTACGTTGATTTTGGGCAGGAGATTCGGATTTTTGGACGGAGAAGTTAGCGAGACGGCTACGCGTCTAGCAGATTCCGTAACTAGTCAGTTCCGAGCATCTCAGGAAGCGTTTTATGGGCTTCCCCTCTGGAAATTAATACCAACAAAGGCGTATAAAGAATTTGTAGCGAGCGAAGACGCTTTATACGACATCGTGTCCGAAATCGTAGACACGGCCCTGATTGATGAGGAACAGTCTTGCACAGACGTACGCAGCGTGTTCGTATCTATACTTCAGGTATCAGGGTTGGACAACAGAGACAAAAAAGCCGCGATTATTGATTACATTGCCGCTGGAATAAAAACACTAGGAAACACCCTTGTGTTTTTATTATACTTGGTTGCCAAACATCCGGATGTACAGGAAAAGATATATAATGAGATATCATTATTGGCTCCAGCAGGTACATCTATTACTGCTGAACATTTACACAAAGCCACATATTTGCACGCGTGTATATCTGAAGCTCACAGACTTCTACCCACTGCTCCATGTATTGCAAGAGTTTTGGAATCGGAAGTCGAATATGACAATTACCGATTGCCATCAGGGACTGTTGTTTTGTTGCACACCGGATTAGCGTGTTTGGAAAATAAAAATTTCATAGATGCTACTTCTTACAAACCGGAAAGGTGGTTAGACGATTTGACGAAAAAATCACCGTTCTTGGTCGCACCGTTCGGTTGTGGCAAACGAATGTGTCCTGGGAAGCGGTTTATAGAATTAGAACTTCAAATTGTTTTAGCAAAGATGGTTAAACAATTTCATATCAATTTTGAGGGACAACTAAAAACTGAATTTGAATTTCTTTTGACACCCAGCAACGCAAATTTTATTCTACGAGACAGAAATTGTTGATGTTTTTAGATACTTTATTAAATTAATCCTACAATAATTTAATATACGTAGTACTTTCATAGGTATATCGTATATTAAATAATAATTAATATTATAACATTGAAATATTTATTTTATTGTACTGC

>U5255

TGGATATCTATGGCGTTCCAACAAAAACTTACCCCCAGGTCCTTGGGGTGTTCCAATTCTCGGTTATCTTCCGTGGCTAGATCCTGAAAGACCTTACAAAACTTTGACGGCCCTGGCTTATAAGTATGGACCGATTTACAGTATTCAAATGGGAAAACATTTTGCAGTCGTTATGTCGGAGCCGACACTAGTTAGAATGGCATTAGCACGGAACGAACTGGCAGACCGCACTAACTTTGAAGTTATCAACGAAATCATGCAAGAACATGGTCTGATATTTACACGCGGACCTCTTTGGAAAGAACAGCGAAAATTTGTTTGCAATTGGCTTAAGGTAATTGGCGTGTCGAAGTTCGGGGAGAAGAAAAATAACTTACAACTACTTATTGCCGACGCCGTGTCTACTACAATATCGAAATTACGAGAGTCAAATACATGTCCCATTGATACAGGAACGTTTTTTCTAGTACAAATAGGAGATTTCATAAATCTCATAGTGTTGGGTAAAGCTTGGCCAGAAGATGACCCTAATTGGGTTTATTTACGAAATTTAGCTGAAGATGGATCCAAAAAATTTGCTATAGCTACTCCATTGAGTGTTTTACCAATGTTGAAGATAATACCTAAGTACAAAAATACTGTTTTTGAAGTAATCGAGGGAGTTAAAAATACGCATTGCATTTACAAAACATTGATGGAGAAACGAGGCAATGAAATCAATGAAAATGACGATCTAATGGCCATGTTCATGAAAGAAATGATAAAAAGAACAAACAACAAAGATTCCCATTTCTTCACTGAAAAACAATGTTGTTTTCTTCTGTCTGATCTTTTTGGTGCTGGTGTAGAAACTTCCGTGAATACTCTAAGATGGTTTCTTCTATACATGGCTTTAAATAAAGAAATACAGAATGAATTACAGACATTATTGGATTCTGTGTGCACAAATGGCGGGATAATAGACTTAGAGCAAATTGAAAACATTCCATTGCTAAAAGCTTGTGTATCTGAAACGATGCGATTACGGCCGGTAGCTCCATCTGGAATACCTCGATCGGTCAACAACGAAATTACACTTGAGGGATACCGCATACCAAAAGGGACCATGGTTTTACCACTGCAGTGGGCAATGCACCACGACGAAAAATATTGGACGGACCCTCTAACATTTCAACCGAAAAGATTTTTAGACGACGAGGGAAATATGATAAACAACAAAGCATTTATGCCATTTCAAGCCGGTAAAAGAGCTTGTGTTGGAGATACGCTTTCCTATTGGATACTCTATTTATTCGGTGCAAATATAATTCATAATTTCAATGTTTCCATGGAAGACGGTTTATCTGAAAAAGAAATTAACACCATCATGGACGGGGAGTTTGGCATAACGCTCAGTCCGGCTACGCACAACATCGTTTTTAAATCTCGAATTTAG

>U7491

CTCAGTTACATCACACTTGTTATTGTTTAAATATTACTTTAATACTTACATTTATGACATTTTGTACGTAAAAATTAAATTATTACTTAGAAATTGGAATTAGTTTTAACCAAATTTTTTCATCTCTAGGCATCACTATAAGTGATAAATTACTAAACTGTATAGGAATCTCAGTCTTTTCACAAGGTTCTACTTCAAATTTTGATAAAATATCTACTAAAGCCAATTTCATTTCTATCTCAGCAAAACGTTTTCCAATACAAATTCTGGGTCCATCGCCAAATGGAATATAAACGCCGTTAGGTCGCTTTGCCTTTTCTTCGTTTGAAAATCTTTCGGGATTGAATACTTCAGGATCACTAAAATATCTAGGATCAAAATGCAATGAAAAGGTTGGAATTATAATTTTCTGTCCTTTCTCAATTTTTAACGAATCGTTCGGCACGCAATAAGATTTTGTAGCTACTCTGAATAAAGCAAACACCACAGGATATTTTCTGAGTGTTTCTGCTATGACCATATCAAGATAATTGAGATTAGTCAAAAATTCATTATCAATTACTCCATTGCATTTAATTTTTGTCAATTTTATCTCTTCGCGTACTCGGTCTTGAATGTTTTTTTTTCAGTGCAAGCTCATATAAACAAAAAGAAAGAGTAGTTGATGTAGTTTCAAATCCAGCAGCAAACAAACCAAATGCATTTGACAATACTTGATCTTTCGTTAATGTTACATCGCTAGATGAATTGTTATTAACAACCAAATCTTGATGGGCTTGAATTAAAGCATGAACAATATCATTCCTCACTATATTATTTTTTTTCTCTGTATTCTATTGTTTCTTTAAATATTGATCCGAAAAATTCAGTTGTATTAGTGGGGAAAACAGAAAGCCTTATAATTTTCAGAATAGCTGGTGATATCATCAATAATATTTCTCTCAGAAAATACATAAAAGATGGTTCAAATGCTTTTTTTCCAAACCTACGAAATGAATAAGTATCATCTTTTGCTACATTTAGCTTTAGTCCAAAAGCACAAGTGCCAATAACATCAGTTGAATATTTCCCCAAAATATCTCGTACATCAATTTCATTGGGATTTTTCTTTAACTCAGAATATATACTATCCATCATATTATCGCCACACTCTTCGATTTGACTATACATCTGTTTAAGTTTGCCAGATGTAAAAGTGGGACTCAACTTATTTCTGAGTTTTTTCCATAGTGGGTTTTCCATAAAAAACAGCGTTTCTGACAGTGGTTCATATGAAAAATTCACATAAATACCACGGTCAGTAAAATACGAAAAATCCTTTATTAGTATGCTGTTGATTATTTCAGGATCGCGCACCATTAAGTATGGAGTACGCATCTGAAACATACCTCCGTACTTGTGTCCAGCCAGTTGGTAATAAACTTTCTTGTACAAGTCTATTGGATGCTCTATGCCCAGTGCTACCTTCAAGTAGTTGCCAAACAAAGGAATTGGACGTATATAAGGCACGTTAAGATTTTTCCATACATTAAATGTCGAAGTACAATAATAATAAATAATCGAGAATGATACTGTAACAAGTATGATCAAGTCAAACCAAATATTAGCAAAGCTAAATAGCATTGACATTTTTCTAATTTGATAGGTATACGAAAGAAAATATAAT

>U7586

GAACGGTCGACAGTCGTCCGGCAGACGTTCGTTCGAACAGTAAAATTGATTTCGCATGTCCGTCTTCTACCCAAAACGATAATGTTTGAATTCGTCTACGAACTGTTCGATCTGAAAATGCTGTTGGTCACCGCGTTCCTCGGTGCCGCGTACATGTACTGCACGTGGACCCACAGCCACTGGTCCAAACTGGGAGTATCAAGTCCGTCGCCGCCGGTGCCGTTGTTCGGGCACGCGATGCCCTCCATGCTGGGCCGGATGCACTTCATGGACGTGCTGCACAACCTTTACCGGCAGCTGGGCGACCAAAGGTTCGGTGGCATTTATACGATGCGAACACCGCAACTGCTCGTCAAAGACCCGGAACTAATAGGACACATACTGATCAAAGACTTCAACAACTTCACAGACCGCGGGTTATACGCCGGCACACACACGAACCCGCTCAACAATAATATATTCTTTACGCGGGGCGAACGGTGGAAAACAATGCGGCAAAAGCTCAGTCCCACGTTCACGGCCAATAAACTGAAATACATGAATGAACAGGTGAAGGAGTGCAGCGACGGTCTGCTGTCGACCATCGGAAAGACTCTGGACGAAGACGCCGGTCGAATCGAAATCCGCGAGATGATGGCCAAGTACTCGACCGACGTGATCGGCAGCTGCGCGTTCGGTCTGAAGCTGGACGCCATCAACGATCCGGACTCGGAGTTCCGGAAACACGGGAAAAACGTTTTCCAGCCGTCGCTGAGGTCCAAACTCCGAGTGGCGGTCATTTTTATGCAGCCGTCCCTGCTGAGCATATTTCGCGTGCATCACTATTCGCACCGCACCATCCGATTCTTCCATGACGCGTTTAAGCAGACAATCGAATACCGGGAGAAGCACAACGAAGACCGCAAAGACTTTGTACAGCATCTGATGAAGGCCAGAGAAGATCTGGTGCTGAATCCGAATCTAAAACCCGAAGAAAAATTTACGGAAATGGATATTGTAGCGAATGCATATATTTTATTTATCGCTGGTTTCGAAACAGTGTCTACATCAATGAGCTTTTGCATGTATGAATTAGCATTAAGAAAAGACGTCCAAGACAAGGTTCGAAAAGAAATATTGGAAGTTAAATCGAAGTATAATGGACAAATGAATAGTGAATGTCTTAACGAACTTCATTATATGGGCATGGTGATTAAAGAAACGTTGAGAAAATATCCTCCATTAGTGACATTAAATCGAGTTGTGACAAAGCCTTATGTAATACCGGGGACACAGATTAAATTAAAAACTGGTACTAAAATCGTGATTCCAGTACACGCCATTCACTACGATCCAAAATACTACTCTGATCCAGAGGCTTTTGAACCGGATCGTTTTTCAGACGAAAATATACACAATTTGCAACCCAACACATATATGCCGTTCGGAGATGGTCCTAGATTTTGTATCGGCAAAAGATTTGCTGAATTTGAAATGAAAATGGCCTTGTCCGAAGTGTTAACTAACTACGAAGTGACGTCATGTGATAAAACCCAAATTCCCATAAAATATGTTATCGGAAGTTTTGTAAATATACCTGAAAGCGTTTGGTTAAAATTTAGGAAATTGAATACTTAATTTTAATATAATAGGTATAGCGTGTATAAATAGGTATTTGTTTATTTTGTATATTGTGTAATCTTTTGACAAAATCTTCT

>U7661

GATAAATGATAACAGAATAATTTATTAATACTTTTAATTTTAAATTAACCCTAAATATTGAATAGCAAAATATTTTGATTGCTATATTATTGATCAACACTTTTCAATGATAAGCGAATTGGTTCATTTGGTGTGAGTAAAATTGACCTTGGTTTAGGTTTAATTGGAATTTGTGTTTCTTTACATGGTGATACTTCGAATTTCGACAATATTTCTGCTAGTCCAGTTTTGGCTTCCATCATCGCAAATCGCAAACCGATACATATCCTAGGTCCATCGCCAAAAGGGAGGAATGTGCCGTGTGGTCTTGAACTTATATTTTCTTCAGTAAATCGTTCAGGATCAAATATTTCTGGGTCTGAATAATACTTGGAATCGTGATGTAATGCATATATGGGAATTATTAATTTTTCACCAACATTTAGAGTTATGTTACTGTCCTCAATTGTGTACTTTTGAGTGCAAACTCGATTTAAAACTGGAACTGGTGGGTGAAGTCTAAATGTCTCATTTATCACCATATCCAAATATTTCATGTCTTTTAACGTATCGTATTCAAGCGTTCCATTGTTTGCGTCCAATGTTTGTTTGATTTTTTCTCTTAATTTTACTTGAATTTCAGGATTCATCGCCAACTCGTATAAACAATAACTTATTGTGGAAGCCGTAGTTTCGAAACCAGCAACAAAAAATACGAAAGCATTTGCTGCTAATATGTCATCAGTAAACAATTTTTGTTCATTTTCTTTAAGTTTTTCTTCTTCTTTCAACTTTACTAAAAGTGCTATAAAATCTTTACGTGAATTTTCTCCATGTCTGTGTTGTTTCATTGTTGTTTCAAGTAGATCATCAAAAAAAGTCATATATGTTGCTTGGCATACCACGTATTCTAAATACATCTAAAAGCCGACCAAAACCAAGTAGTCTGGTTAAATTTAGTAAAGTCGATCGAAGTCTCGGCATAAGAATCTCTCGTCCTGTACGACGAAATTCTGAATCTGGATTTGATAATGTATTACAGTCTAGACCAAAAGCACATCGGCCAATAGTGTCAGTTGTAAATTGAGCCGCTGCTTCACTCACATCAACAATTCCATTGTTGTTCGTTATCTGCCCGTTCAAGTTGTCGATCAGACGCGCCGTACACACGTTTATGTCACCTAACATGGACTTGAGTTTGGCCGCGGAAAAAGTCGGACTCAACTTGGCCCTGATGGCTTTCCACTGATCTCCTCGTAAGTTCACCAGGTGATCGAACAGCTTGTCGTGCACGAACGACTCCTTGCTTGCGTTTCGGTCGACGAACGATCGGAAGTCCTTTACCATGACGGTGTGCACTAGCTGCGGGTCGCGAACCACCAACAGCGGTTGGCGTCCCTCGAAGATGCCGAAATACCGTTGGCCGTCGAACTCGCGGTACAGCGAATGGATGACTTCTACGAGCGGTGCGCGGCCCGCGACAACATCGCCCAAGCTGCCGAATGGCGGCGTCGGCACGGCATGCGGTACTCCGAGCTTTGTCCACTTGCCGTAGTGGTACGTAAGGTAAACGTAGCTCAGGAACACGACCAGCAGAACGGCGGCGGCGTACAGTTGGACGAACGGAATCACGTCCGACATGGCGAATCCAACCGCTTGGCCGGATGCAGACGGGATATTGCAGGTAACGCGCGTACGCAACGGCCAATATGCACGTCCGTCCGCACAGCGCAAGATCAACAGTATAAAGAATTATCAATATTGACTTTATCGGTGCACGCGATTCCCCTTCACCGGCATAACTGAAACTACTTGCACGCGACGTGTAGATCGGTCGGTGGTACCGTCGCTGAACTTGAACGGTACAACTGTAGTGATGGTGTAAATTATTATCAATATTATTATCATTAACAAGACGATTTATAATCGAGTGCGACGGAGATCACTGTAATACCGCTCAACTGTCTAGAATATTTGTGTTGTTTTCCGCGCTCGTGTATTTTATACTAAATTATTTTATTAATACCTATAATCTATATTATGTATATTAAGATTAGAAAGAGGAG

>U8071

CGTTATTTTATTGTATCGTTCATTCTACGAAGTTCTTGGTATTATTTTGACATAACAGCCGTCAACAAACTTAAGTGTCGCTAAGAACATTAATCTTAGGTCTTTCGGTGTGGGACATCTGTCCGACGGAAAGAATGTATTTTTTCGTACTAGTGTCGATATAACGGTTTTCATTTGGAGCATGCCATATTTGATTCCGATACAGTTCCTATATCCCGCACTGAATGGTATAAATGTATATGGATGTCGGCTGTGATATGTATCTGGTAAGAAGTTGTCAGGGTTAAACTTTTCTGGATCATTGTAGTACTGCTCACTCGAATGTAAAAATAGTGGACAGACCATCAACGTTGAACCGGCTGGACACAAATATTCTCCGATTTTCATTTCCTCATCTAAGCTTCTACCATACACGGGCAAAGGAGGGAACAATCGTAATGTTTCTTTGATCACTCGTTCTAGGTATTCCATTTGTTGTAAATCTTCATATGTTGGTGATCTATTCTGATCGCCGTTCGAAAAAATCGATTGCAATTCCTCAAACACCTTGTTCTGTACATCTGGATGATGAGCTAACATAAAAATTGCACATGCATTGGCCATAGCAGTAGTTTCTTGGCCTCCAATCATGATGGTCACAAGTTCATCTCGTATTTG

>U8825

TTTTTTGTAGTATTGAATAGTATTATATTTTCTAATTTTAAAAATAGCATAATATATAATATTGGAAAAAATGGACATATCAATATGGATATTGTTGGTATATTATGCTAAATAATGTTAATTTACAACGAGCGGTTTAAATTTTAAATGAATGCCGTTTTTTTGGACTGACGATACTTCCAGGACTTCCTATGATTAGAGGTATTTCCGTTTTTTCGCACGGCAATACTTCATATTTTGATAATATTTCCGTTAGAACAAATTTCATTTCTAATTCCGCCAACCGTTTACCTATGCACAATCTCGGTCCGTCGCCAAACGGTATGTAAGTGCCATTGAGTCGTTTAGATTTTTCTTCCGCGGAAAATCTTTCAGGATCAAAAGTGTTGGGATTGGGATAATATTTTGGATCGTTATGTATGGCGAGTATTGGTATGACGATTTTTTGTCCCTTTTCAATGACTAACGACTGACCGGGTACCTTGTAATTCTTTGTTGCTTGTCTGAGTATGATTAAAGTGATAGAGTATTTACGCGATGCTTCTTCCAAAACCATGTTCATGTAACGGAGATCAGTCAAAAAGTTGTTGTTCAAATTTCCTCCGTATTTCGCTTTTGTCGTCACGATCTCTTCGCGCAGCCTATCTTGGACTTCTTTGTTCACTGCCAGCTCGTACAAACAAAAACATGTCAAGGTAGAAACCGTTTCGGACCCAGCGACAAACATCAGTACCGCGTTTGCGATTATGTCTATTTCGGCCCATTTGTTTTCGGACGTTGAGTCGTTATTTAACACCAAATCATTCCTAGCTTGTATCAGGGTTTGCGTGACGTCGTTCCTGATAACGTTGTTCCGGCTCCTGTATTCGAGGACTTCTTTGAACATAGATCCATAAAATTCAGACACGTCCTCCGGGAACTGTTTTAGCTTTAGAGCAAACACCAATTTTGGACATACCAGCAAAAGAGCCTGAATAAAAAGCTGTTTTAAGTTTCCCTGGAATAATTTCTTGCCGAACTTGCGGAAGTCTGAATTTCCGTTTTTTATCGTGTCCAATTTCAGACCGAAAGCCGATGCCCCGATGACGTCCGTTGAGAAGTTTGCAATTATCTCTCGCAGTTCGAAGTGATCACAGGTTTTCAACTTATCGTCGATGACGTTCATAAGTTGCTCAATGCACCCCTTGATCTGTTCATGCGTGTCCTTAAGTTTACCCGAGGTGAACCCGGGACTGAGCTTCTGTCGCATCGTTCTCCACCGATCGCCGTTCAATAAGAACAGGCTGTTTGCTAAGATGTTGACAGACGGATGAGTATCTACACCATGGTCTGTGAAGTACGAGAAGTCTTTGACCATTATGCTGTTGATTAGCTCTGGATCGCGTAACATCAGGAACGGCGTAGTCATTTGGTAGAAACCACAAAGTTTTTCACCCGAAAATTGGTTGTAAATACGACTGAAAAAGTGTATCGGTTCCTCCAAGGTCAAGACCATTTTCATTGAGTTGCCAAACAGTGGTAAGGGCGGCGGGTAGGGTACATTCAGTTTGCTCCATTTATCGAGAGTGTACGTTGAAAAGTAGTAGGTGATCGATAGAAAGACCAATAATACATACAATACGTACACGGAACTGAACAGTTCGATAATCAAACTCGGAAAAATTTCAATCATATTGTAACGGAAATGTACAATTAATAGGTGTATAAATTTCCGATATTATTACTTTAAAATAGAAAGAGTTTTTAAGAACATCCGTGCGTGACTGCGAATACTTTATGAAGCATAGTTTTG
