# Supplementary material for: Ecological Adaption Analysis of the Cotton Aphid (Aphis gossypii) in Different Phenotypes by Transcriptome Comparison
Source: PLoS One. 2013 Dec 23;8(12):e83180. doi: 10.1371/journal.pone.0083180 (PMC3871566; doi:10.1371/journal.pone.0083180)
Supplement: File S2 — The reaction conditions for first- and second-strand cDNA synthesis, end repair, and adaptor ligation. (DOCX) [file pone.0083180.s013.docx]

**Additional file 2:** The reaction conditions of synthesize first-strand cDNA and second-stand cDNA and end repair and ligation of adaptors.

**First-strand cDNA Reaction conditions:**

1. Assemble the following reaction:

- N6 primer (3ug/µL) 1 µL
- mRNA (100ng/µL) 10.5µL

1. Incubate the tube in a PCR thermocycler at 65°C for 5 minutes, and put the tubes on ice.
2. Mix the following in order, make 10% extra reagent for multiple samples:

- 5× 1^st^ strand buffer 4µL
- 100mM DTT 2µL
- dNTP mix (10mM) 1µL
- RNAseOUT (40U/µL) 0.5µL

1. Add 7.5µL mixture to the tube, mix well, and heat the sample at 25°C in a thermocycler for 2 min.
2. Add 1 µL SuperscriptII (200U/ µL) to the sample, and incubate the sample in a thermocycler with following program:

- Step 1 25°C 10min
- Step 2 42°C 50min
- Step 3 70°C 15min
- Step 4 4 °C Hold

**Second-strand cDNA Reaction conditions:**

- - 1. Put the tubes on ice.
    2. Add 61µL of H_2_O to the first strand cDNA synthesis mix.
    3. Add the following reagents:
- 10 × second strand buffer 10µL
- dNTP mix (10mM) 3µL
  - 1. Mix well, incubate on ice 5 minutes or until well chilled, and add:
  - RNaseH (2U/µL) 1µL

##### DNA pol I (10U/µL) 5µL

- - 1. Mix well, and incubate at 16°C in a thermomixer (spin at 1400rpm for 15sec and stand for 2min) for 2.5 hours.
    2. Purify the DNA with a Qiaquick PCR spin column, and elute in 30µL of EB solution.

**Endrepair and ligation of adaptors Reactions:**

**1. Prepare the following reaction mix:**

- Eluted DNA 30µL
- H_2_O 45µL
- T4 DNA ligase buffer with 10mM ATP 10µL
- dNTP mix (10mM) 4µL
- T4 DNA polymerase (3U/µL) 5µL
- Klenow DNA polymerase (5U/µL) 1µL
- T4 PNK (10U/µL) 5µL

Incubate at 20°C for 30min.

Purify the DNA with a Qiaquick PCR spin column, and elute in 32µL of EB solution.

**2. Addition of A-tailing**

Prepare the following reaction mix:

- - Eluted DNA 32µL
  - Klenow buffer 5µL
  - dATP(1mM) 10µL
  - Klenow 3’ to 5’ exo- (5U/µL) 3µL

Incubate at 37°C in for 30min.

Purity the DNA with a Qiaquick minElute column, and elute in 10µL of EB solution.

**3. Adaptor ligation**

Prepare the following reaction mix:

- - Elute DNA 19µL
  - DNA ligase buffer 25µL
- Adaptor oligo mix 1µL
- DNA ligase (1U/µL) 5µL

Incubate at RT for 15min.

Purify the DNA with a Qiaquick minElute column, and elute in 10µL of EB solution.

**PCR Reaction Conditions:**

Set up PCR mix,

- 5 × cloned Pfu Buffer 10µL
- PCR primer 1.1 1µL
- PCR primer 2.1 1µL
- 25mM dNTP mix 0.5µL
- Pfu polymerase 0.5µL
- H2O 27µL
- ligated-DNA 10ul

Run following PCR cycle:

- 98°C 30 sec
- 98°C 10 sec
- 65°C 30 sec 15×
- 72°C 30 sec
- 72°C 5 min
- 4°C ∞

Purify the DNA with a Qiaquick column, and elute in 50µL of EB solution.
