# Supplementary material for: Ecological Adaption Analysis of the Cotton Aphid (Aphis gossypii) in Different Phenotypes by Transcriptome Comparison
Source: PLoS One. 2013 Dec 23;8(12):e83180. doi: 10.1371/journal.pone.0083180 (PMC3871566; doi:10.1371/journal.pone.0083180)
Supplement: File S3 — Trinity assemble process. (DOCX) [file pone.0083180.s014.docx]

**Additional File 3:** The assemble process of Trinity (version is 20120608).

Transcriptome de novo assembly is carried out with short reads assembling program – Trinity [[1](#_ENREF_1)]. Trinity combines three independent software modules: Inchworm, Chrysalis, and Butterfly, applied sequentially to process large volumes of RNA-seq reads. Trinity partitions the sequence data into many individual de Bruijn graphs, each representing the transcriptional complexity at at a given gene or locus, and then processes each graph independently to extract full-length splicing isoforms and to tease apart transcripts derived from paralogous genes. Briefly, the process works like so:

**Inchworm** Assembles the RNA-seq data into the unique sequences of transcripts, often generating full-length transcripts for a dominant isoform, but then reports just the unique portions of alternatively spliced transcripts.

**Chrysalis** Clusters the Inchworm Contigs into clusters and constructs complete de Bruijn graphs for each cluster. Each cluster represents the full transcriptonal complexity for a given gene (or sets of genes that share sequences in common). Chrysalis then partitions the full read set among these disjoint graphs.

**Butterfly** Then processes the individual graphs in parallel, tracing the paths that reads and pairs of reads take within the graph, ultimately reporting full-length transcripts for alternatively spliced isoforms, and teasing apart transcripts that corresponds to paralogous genes.

The result sequences of trinity is called Transcriptss. When multiple samples from a same species are sequenced, Transcriptss from each sample's assembly can be taken into further process of sequence splicing and redundancy removing with sequence clustering software to acquire non-redundant Transcriptss as long as possible. Then do gene family clustering, the Transcriptss will be divided to two class. One is clusters, which the prefix is CL and the cluster id is behind. In one cluster, there are several Transcriptss which similarity between them is more than 70%. And the other are singletons, which the prefix is Transcripts.

In the final step, blastx alignment (evalue < 0.00001) between Transcriptss and protein databases like NR, Swiss-Prot, KEGG and COG is performed, and the best aligning results are used to decide sequence direction of Transcriptss. If results of different databases conflict with each other, a priority order of NR, Swiss-Prot, KEGG and COG should be followed when deciding sequence direction of Transcriptss. When a Transcripts happens to be unaligned to non of the above databases, a software named ESTScan [[2](#_ENREF_2)] will be introduced to decide its sequence direction. For Transcriptss with sequence directions, we provide their sequences from 5' end to 3' end; for those without any direction we provide their sequences from assembly software.

1. Mortazavi A, Williams BA, McCue K, Schaeffer L, Wold B (2008) Mapping and quantifying mammalian transcriptomes by RNA-Seq. Nat Methods 5: 621-628.

2. Iseli C, Jongeneel CV, Bucher P (1999) ESTScan: a program for detecting, evaluating, and reconstructing potential coding regions in EST sequences. Proc Int Conf Intell Syst Mol Biol: 138-148.
